# Discovery of a small peptide that increases yeast lifespan by enhancing the function of APC^Cdh1^ in nondividing quiescent cells

**DOI:** 10.1093/genetics/iyag249

**Published:** 2026-09-16

**Authors:** Rachel E Harris, Spike D Postnikoff, Nishita K Shukla, Alexandra H Harkness, Hannah M Brooks, Abd-Al-Wahab Khawaja, Mackenzie E Malo, Carlos Zapien-Verdugo, Brandon M Waddell, Cheng-Wei Wu, Troy Anthony Alan Harkness

**Affiliations:** Department of Biochemistry, Microbiology and Immunology, College of Medicine, University of Saskatchewan, Saskatoon, SK S7N 5E5, Canada; Department of Medicine, Division of Geriatric Medicine, University of Alberta, Edmonton, AB T6G 2S2, Canada; Department of Medicine, Division of Geriatric Medicine, University of Alberta, Edmonton, AB T6G 2S2, Canada; Department of Anatomy and Cell Biology, McGill University, Montreal, QC H3A 0C7, Canada; Department of Anatomy and Cell Biology, University of Saskatchewan, Saskatoon, SK S7N 5E5, Canada; Department of Anatomy and Cell Biology, University of Saskatchewan, Saskatoon, SK S7N 5E5, Canada; Department of Anatomy and Cell Biology, University of Saskatchewan, Saskatoon, SK S7N 5E5, Canada; Department of Veterinary Biomedical Sciences, Western College of Veterinary Medicine, University of Saskatchewan, Saskatoon, SK S7N 5B4, Canada; Department of Veterinary Biomedical Sciences, Western College of Veterinary Medicine, University of Saskatchewan, Saskatoon, SK S7N 5B4, Canada; Department of Veterinary Biomedical Sciences, Western College of Veterinary Medicine, University of Saskatchewan, Saskatoon, SK S7N 5B4, Canada; Toxicology Centre, University of Saskatchewan, Saskatoon, SK S7N 5B3, Canada; Department of Biochemistry, Microbiology and Immunology, College of Medicine, University of Saskatchewan, Saskatoon, SK S7N 5E5, Canada; Department of Medicine, Division of Geriatric Medicine, University of Alberta, Edmonton, AB T6G 2S2, Canada; Department of Anatomy and Cell Biology, University of Saskatchewan, Saskatoon, SK S7N 5E5, Canada

**Keywords:** *S. cerevisiae*, anaphase-promoting complex, Cdh1, peptides, genetics, quantitative aging assays, *C. elegans*

## Abstract

Aging is characterized by molecular hallmarks conserved from yeast to humans. One such hallmark, senescence, is an irreversible nondividing state that promotes aging. We previously reported that mutants of the yeast anaphase-promoting complex (APC) shorten the lifespan of both dividing and nondividing cells. Here, we propose that activation of the APC will promote the maintenance of quiescent yeast cells, thereby delaying senescence and aging. We observed that APC activity in aging quiescent cells becomes increasingly impaired, as the APC substrates Clb1 and Mps1 accumulated within aging cells reintroduced back into the cell cycle. To identify peptide activators of the APC, we used a yeast 2-hybrid screen to recover peptides that interacted with the APC subunit Apc10. Recovered peptides were tested for their effects on the replicative lifespan (RLS) of dividing cells and the chronological lifespan (CLS) of nondividing cells. Several peptides increased the RLS of wild-type cells, but only one peptide (C43-4) increased CLS, which requires the APC Cdh1 coactivator but not the Cdc20 coactivator. In cells expressing C43-4, Clb1 and Mps1 levels remained low when aging quiescent cells were reintroduced back into the cell cycle. The addition of a synthetic C43-4 to aging quiescent cells increased CLS even when added at late stages of aging. Mutations to APC subunits and coactivators blocked the ability of C43-4 to extend CLS. Htz1, which shares sequence homology with C43-4, was found to suppress APC mutant phenotypes and stabilize Apc10. We individually mutated the 6 amino acids in C43-4 that were shared with Htz1; one of the mutants disrupted the C43-4-Apc10 2-hybrid interaction and abolished the C43-4-dependent increase in CLS. C43-4 action is evolutionarily conserved, as C43-4 increased *Caenorhabditis elegans* lifespan in a *daf-16*- and *aak-2*-dependent manner. Our results describe the discovery of an evolutionarily conserved translational peptide that is the first of its kind to specifically activate the anti-aging APC^Cdh1^ complex.

## Teaser

We understand what happens when a cell ages, but we do not know why a cell ages, or how to stop it. To gain insight into whether aging can be slowed, Harris et al. discovered a peptide that binds to and activates an anti-aging complex in yeast, called the APC. This peptide increases the activity of the APC, requires the APC for function, and increases lifespan in yeast and worms. They conclude that cellular health and lifespan can be therapeutically enhanced. The conserved nature of the peptide suggests that this may slow human aging, and possibly delay the onset of age-related disease.

## Introduction

Loss of genomic stability and proteostasis are key aging hallmarks, observed from yeast to humans, that push quiescent cells (also referred to as G0 or stationary phase cells) into senescence (López-Otín et al. 2023). As genomic stability and proteostasis erode in aging cells, DNA mutations and aneuploidy, as well as misfolded and aggregated proteins, begin to accumulate (Kennedy et al. 2014; Harkness 2018; Guo et al. 2022; Sanada et al. 2025). The accumulation of cellular damage typically results in the transition from quiescence to senescence, followed by the release of molecules that damage neighboring cells, and finally, death through apoptosis. Senescence is a major driver of aging, with senescent cells accumulating in the aging population (Ovadya et al. 2018; Song et al. 2020). In recent decades, medical improvements and lifestyle changes have driven an increase in the mean human lifespan (Li et al. 2018; Olshansky et al. 2024). However, the extra years of life are often fraught with a progressive increase in disease (Niccoli and Partridge 2012), suggesting that without a means to prolong the life of quiescent cells and slow cellular aging, it is unlikely that the human health span will continue to increase. Therefore, to increase human health span and live longer without disease, it is vital to discern how to protect quiescent cells from the inherent damage that occurs to DNA and proteins during aging.

Proteostasis, the combined processes that work together to maintain protein folding, degradation, and trafficking, is vital for the maintenance of cellular integrity (Morimoto and Cuervo 2014). The ubiquitin-proteasome system orchestrates the selective degradation of key regulatory proteins (Kevei et al. 2017; Sampaio-Marques and Ludovico 2018) and plays a pivotal role in maintaining the appropriate complement of proteins in the cell. As the cell ages, proteostasis erodes, largely due to impaired proteasome- and autophagy-dependent removal of misfolded and aggregated proteins (Kaushik and Cuervo 2015; Akbergenov et al. 2026). Among the critical regulators of proteostasis during the cell cycle is the anaphase-promoting complex (APC), an essential and highly conserved ubiquitin ligase (E3) that targets protein substrates for degradation. The APC ensures proper exit from mitosis, entrance to G1/G0, and protection against chromosomal instability (Greil et al. 2022; Hu et al. 2022; Zhang et al. 2025). The significance of APC function extends beyond cell cycle control, as recent evidence indicates its involvement in stress response regulation during the yeast quiescent stationary phase and its potential role in aging processes (Chen et al. 2012; Postnikoff et al. 2012; Harkness 2018; Hu et al. 2022). Elevated levels of APC substrates have been observed in various cancers, underscoring the impact of APC dysregulation on diseases and cellular aging (VanGenderen et al. 2020, 2026; Arnason et al. 2022; Jeong et al. 2023; Lubachowski et al. 2024).

The APC takes the form of 2 temporally distinct complexes, APC^Cdc20^ and APC^Cdh1^ (APC^Cdc20^ in yeast, APC^CDC20^ in mammalian cells); APC^Cdc20^ is responsible for entrance to and transition through mitosis, while APC^Cdh1^ triggers mitotic exit and maintains the G1/G0 state until cells are ready for DNA replication (Greil et al. 2022; Hu et al. 2022; Zhang et al. 2025). Since APC^Cdh1^ is critical for entrance into G0 and maintenance of quiescent cells (Hu et al. 2011; Chen et al. 2012; Eguren et al. 2013; Lafranchi et al. 2014), we propose that activation of APC^Cdh1^ will prolong the quiescent state, delaying the transition into pathogenic senescence. APC^CDC20^ and APC^CDH1^ activity is tightly regulated by several competing intercellular factors, such as RAS/PKA, MAPK, PLK, and SIRT2 (Yamashita et al. 1996; Kotani et al. 1998; Zhou et al. 2016; Harkness 2018; VanGenderen et al. 2020). A major APC inhibitor is the spindle assembly checkpoint (SAC) that sequesters Cdc20 away from the APC during prometaphase until all spindles are attached to kinetochores at the metaphase plate (Kapanidou et al. 2017). Once all chromosomes are attached to the metaphase plate, the SAC is satisfied and Cdc20 is released, allowing it to interact with the APC. Defects in SAC signaling and function are associated with cancer and aging (Brown and Geiger 2018). Current research indicates that premature activation of APC^Cdc20^ via SAC inhibition in cancer cells promotes rapid entry into mitosis, causing cells with high levels of aneuploidy to undergo mitotic catastrophe and apoptosis (Choi et al. 2017; Szymiczek et al. 2017; VanGenderen et al. 2020; Lubachowski et al. 2024). SAC inhibitors have been developed as anticancer therapeutics and are currently entering clinical trials (Lima et al. 2025). However, SAC inhibitors activate APC^CDC20^, but not APC^CDH1^. Interestingly, mutations in APC subunits have been found to generate resistance to SAC inhibitors *in vitro*, providing evidence that an active APC is required for these inhibitors to work (Thu et al. 2018).

While SAC inhibition is currently considered as therapy against cancer, activation of the proteasome is also considered to have therapeutic potential to delay aging and promote cell health (Upadhyay and Joshi 2024). Inhibition of the proteasome also targets cancer cells for death (Zhou et al. 2023), highlighting the fine line proteostasis plays in cancer cell growth. Based on observations that mammalian APC^CDH1^ contributes to tumor suppression (Garzón et al. 2017; Sansregret et al. 2017; Thu et al. 2018) and yeast APC^Cdh1^ is required for stress response and longevity (Harkness et al. 2004; Postnikoff et al. 2012; Menzel et al. 2014; Malo et al. 2016), we focused on the development of APC^Cdh1^ activators. To do so, we used a yeast 2-hybrid (Y2H) screen (VanGenderen et al. 2026) to discover small peptides that interacted with the APC subunit Apc10. We subcloned yeast-derived APC-binding peptide constructs into a human expression vector and discovered that one in particular, called C43-4, was capable of stalling multiple drug-resistant (MDR) cancer growth *in vitro* and in mice growing breast cancer cells *in vivo*. In the study described here, we characterized Apc10-binding peptides for those that could increase yeast lifespan and reverse impairment of APC activity as cells age. We show that C43-4 is the only peptide tested that could activate APC^Cdh1^.

## Materials and methods

### Yeast strains, media, and basic methods

*Saccharomyces cerevisiae* strains used in this study were all haploid and are listed in Table 1. Yeast cells were cultured at 30 °C in rich medium [YPD—1% yeast extract, 2% peptone, and 2% glucose {dextrose} {Fisher Scientific, BD 242820}] or in synthetically defined (SD) medium [0.17% yeast nitrogen base without amino acids {Fisher Scientific, BD 233520}, 0.5% ammonium sulfate, and 0.13% essential amino acids, with the carbon source maintained at 2%, either with 2% glucose, sucrose, galactose, or mixtures of sucrose and galactose, as indicated]. Omission of specific amino acids provided selection pressure for the maintenance of transformed plasmids containing auxotrophic markers (plasmids used in this study are shown in Table 2). Complete media (CM) was the same as SD except all amino acids were included. Minimal media (MM) was prepared the same as SD, except with only 0.013% supplementation of necessary amino acids, using a 10-fold reduction in available amino acids to increase cellular stress and shorten lifespan. Selection of the KanMX module, used to replace wild-type genes, required 0.2 mg/mL of geneticin (G418 sulfate; [Gibco, 11811023]) added to YPD. XL1-Blue chemically competent cells (Agilent #200249) were cultured in LB broth [0.5% yeast extract {Gibco, 212750}, 1% tryptone {Gibco, 211705}, and 1% NaCl {Fisher, BP358-1}] for basic plasmid extraction from bacterial cells. To make solid media, 2% agar was added to the liquid medium prior to autoclaving. Molten agar was cooled to approximately 55 to 60 °C (2% sugars were added to media after cooling) before pouring into petri dishes (VWR, 25384-302). For long-term storage, cells were grown to log phase in YPD, suspended in 16% glycerol, and stored at −80 °C. Yeast transformations were performed using the LiAc method as previously described (Gietz and Schiestl 2007). Yeast genomic DNA extraction was conducted using a LiAc-SDS method according to a published protocol (Lõoke et al. 2011). Bacterial DNA preps, Western blots, and PCR were performed according to previous methods (Malo et al. 2016). Replicative lifespan was conducted as published previously (Menzel et al. 2014; Postnikoff and Harkness 2014).

**Table 1.** List of yeast strains used in this study.

Strain	Genotype	Genetic background	Source/reference
**YTH1**	MATa *leu2-3,112 trp1-1 can1-100 ura3-1 ade2-1 his3-11,15*	W303	W. Neupert
**YTH2**	MATα *leu2-3,112 trp1-1 can1-100 ura3-1 ade2-1 his3-11,15*	W303	W. Neupert
**YTH3863**	YTH1 with *cdc20-1*	W303	A. Amon
**YTH4667**	YTH1 with *apc10Δ::KanMX*	W303	This study
**YTH4670**	YTH1 with *cdh1Δ::KanMX*	W303	This study
**YTH4940**	YTH1 with *cdh1Δ::KanMX*	W303	This study
**YTH4948**	MATa *ade2 his3 leu2 trp1 ura3 cdh1Δ::KanMX*	W303	This study
**YTH4959**	MATα *ura3 trp1 leu2 his3 ade2 can1 cdc20-1*	W303	This study
**YTH5065**	MAT(?) *leu2-3,112 trp1-1 can1-100 ura3-1 ade2-1 his3-11,15 apc5^CA^::HIS*	W303	This study
**YTH4504**	MATa *his3Δ1 leu2Δ0 met15Δ0 ura3Δ0*	S288c (BY4741)	Invitrogen #95401.H2
**YTH4990**	BY4741 with CLB1-TAP	S288c	Horizon Discovery
**YTH4992**	BY4741 with MPS1-TAP	S288c	Horizon Discovery
**YTH5020**	BY4741 with *apc10Δ::KanMX*	S288c	Invitrogen #95401.H2
**YTH5071**	BY4741 with APC5-TAP	S288c	Horizon Discovery
**YTH5076**	BY4741 with SIC1-TAP	S288c	Horizon Discovery
**YTH5103**	BY4741 with APC10-TAP	S288c	Horizon Discovery
**YTH5113**	BY4741 with *trp1Δ::KanMX*	S288c	Invitrogen #95401.H2
**YTH5115**	BY4741 with *his3Δ::KanMX*	S288c	Invitrogen #95401.H2
**YTH5122**	BY4741 *CLB1-TAP HA-C13-3-TrxA*	S288c	This study
**YTH5123**	BY4741 *CLB1-TAP HA-C43-4-TrxA*	S288c	This study
**YTH5125**	BY4741 with *apc10Δ::KanMX*	S288c	Invitrogen #95401.H2
**YTH5126**	BY4741 with *cdh1Δ::KanMX*	S288c	Invitrogen #95401.H2
**YTH5127**	BY4741 with *apc9Δ::KanMX*	S288c	Invitrogen #95401.H2
**YTH5128**	BY4741 with *cdc26Δ::KanMX*	S288c	Invitrogen #95401.H2
**YTH5129**	BY4741 with *mnd2Δ::KanMX*	S288c	Invitrogen #95401.H2
**YTH5130**	BY4741 with *swm1Δ::KanMX*	S288c	Invitrogen #95401.H2
**YTH5131**	BY4741 with *ama1Δ::KanMX*	S288c	Invitrogen #95401.H2
**YTH2066**	MATa *leu2-3,112 trp1-1 can1-100 ura3-1 ade2-1 his3-11,15*	Derived from W303 x S288c	This study
**YTH2078**	MAT? *leu2-3,112 trp1-1 can1-100 ura3-1 ade2-1 his3-11,15 apc5^CA^::HIS*	Derived from W303 x S288c	This study

**Table 2.** List of plasmids used in this study.

Plasmid	Vector	Insert	Source/reference
**pJG4-5**	*2μ-TRP1*	*GAL1prom*	C.R. Geyer
**pJG4-5-C2-4B**	*2μ-TRP1*	*GAL1prom-HA-C2-4B*	This study
**pJG4-5-C3-1**	*2μ-TRP1*	*GAL1prom-HA-C3-1*	This study
**pJG4-5-C3-1B**	*2μ-TRP1*	*GAL1prom-HA-C3-1B*	This study
**pJG4-5-C4-3**	*2μ-TRP1*	*GAL1prom-HA-C4-3*	This study
**pJG4-5-C9-5**	*2μ-TRP1*	*GAL1prom-HA-C9-5*	This study
**pJG4-5-C13-3**	*2μ-TRP1*	*GAL1prom-HA-C13-3*	This study
**pJG4-5-C43-4**	*2μ-TRP1*	*GAL1prom-HA-C43-4*	This study
**pRS416**	CEN-URA3		ATCC #87521
**pRS416-C43-4**	CEN-URA3	*GAL1prom-C43-4-HA*	M. Patel
**YEplac181**	*2μ-LEU2*		W. Neupert
**YEplac181-C43-4**	*2μ-LEU2*	*GAL1prom-HA-C43-4*	M. Patel
**pYEX-4T-1**	*2μ-URA3*	*CUP1prom*	Clontech #6199-1
**pYEX-4T-1-APC5**	*2μ-URA3*	*CUP1prom-GST-APC5*	Clontech #6199-1
**pYEX-4T-1-APC10**	*2μ-URA3*	*CUP1prom-GST-APC10*	Clontech #6199-1
**pYEX-4T-1-HTZ1**	*2μ-URA3*	*CUP1prom-GST-APC10*	Clontech #6199-1
**pMWU-C43-4-GFP**	pDEST-eft-3p	SV40-HA-TrxA-C43-4-GFP	This study

### Yeast NaOH protein extraction

Prior to sample preparation, 4× sample loading buffer was prepared [0.2 M Tris base {Sigma-Aldrich, 252859}, 0.4 M DTT {Fisher, FERR0861}, 277 mM SDS {BioShop, SDS999.1}, 6 mM Bromophenol blue {Sigma-Aldrich, 114391}, and 4.3 M glycerol {Fisher, BP229-1}], stored at −20 °C, and diluted to 1× as needed. The absorbance at an optical density of 600 nm (OD_600_) of all yeast cultures was measured using a SmartSpec (Bio-Rad SmartSpec Plus Spectrophotometer), and 1 to 5 mL of culture was centrifuged at 5000 × *g* for 3 min. Up to 5 mL was taken if the initial OD_600_ reading was below 1.0 to have enough protein for extraction, depending on the strains grown and absorbance recorded. The supernatant was removed, and pellets were resuspended in 500 μL of ice-cold 0.5 M NaOH (Sigma-Aldrich, SX0607N). Cell suspensions were incubated on ice for 10 min and centrifuged at 13,000 × *g* for 2 min at 4 °C before removing the supernatant. Samples were resuspended in 1× sample loading buffer as follows: $\frac{\left(\mathrm{OD}600\;\mathrm{of}\;\mathrm{culture}\right)}{40\;}\;\times \;(\mathrm{mL}\;\mathrm{of}\;\mathrm{culture}\;\mathrm{taken}\;\times \;1000)\;$ = μL of 1× sample loading buffer to add. Samples were frozen at −20 °C overnight and then thawed, mixed, and heated at 80 °C for 10 min. After heating, samples were chilled on ice for 2 min and centrifuged at 13,000 × *g* for 5 min at 4 °C before transferring the protein-containing supernatant to a new tube. Samples were used for subsequent analysis and stored at −80 °C.

### Western blotting

Western blots were performed according to our published methods (Menzel et al. 2014; Malo et al. 2016), with updates provided. A total of 15 μL of protein lysates were separated through 10% SDS-PAGE gels with a BLUelf prestained protein ladder (FroggaBio, PM008-0500-G) in 1× SDS running buffer. Electrophoresis was performed at 150 V for 60 to 90 min, depending on the thickness of the gel cast. Total protein was visualized by placing the gel cast with 0.5% trichloroethanol (TCE) on a benchtop UV transilluminator (VWR Compact UV Transilluminator) and irradiating for 1 to 2 min to induce covalent modification of tryptophan proteins to shift fluorescent emission to the visible range (Ladner et al. 2004). Gels were imaged with a Bio-Rad VersaDoc MP molecular imager to capture total protein loaded in each lane. Separated proteins on these gels were transferred for 30 to 50 min at 20 V to a nitrocellulose membrane (Bio-Rad, 1620115), which was preactivated by a 10 min incubation in 1× transfer buffer. After transfer, if the transilluminated total protein load was not captured prior to transfer, membranes were submerged for 1 to 2 min in Ponceau S stain to visualize and scan total protein. Ponceau S stain was washed away with phosphate-buffered saline with 0.1% Tween-20 (PBST), and membranes were blocked for 30 to 60 min on an orbital shaker (Corning LSE Platform Rocker) at room temperature (RT) with blocking buffer (5% skim milk in PBST). Blocked membranes were then submerged in primary antibodies against TAP (Thermo Fisher, CAB1001), HA (Abcam, ab9110), GAPDH (Proteintech, 60004-1-Ig), histone H3 (Proteintech, 17168-1-AP), Clb2 (Y180, Santa Cruz, SC-9071), GST (J90, described in Turner et al. 2010), or FITC (Proteintech, 80003-1-RR) diluted 1:1,000 in blocking buffer (except GAPDH, which was diluted 1:10,000) and incubated for 3 h at RT or overnight at 4 °C. Following incubation, membranes were washed with PBST for 3 10 min washes prior to incubation with HRP-conjugated secondary antibodies diluted 1:10,000 with blocking buffer for 30 min at RT (Rabbit HRP-conjugated antibody: Bio-Rad, 1706515; Mouse HRP-conjugated antibody: Bio-Rad, 1706516). Another 3 10 min PBST washes were performed before membranes were imaged with a Bio-Rad VersaDoc MP molecular imager, utilizing Western ECL substrate (Bio-Rad, 170-5060) to detect chemiluminescence. Images were analyzed and quantified using ImageJ software, performed by measuring the signal in a region of interest, with the background signal subtracted. Quantified protein abundance was normalized to total protein using either TCE or Ponceau S images, or using GAPDH as a loading control. See Supplementary Figs. 2, 4, 6, 11, and 12 for all uncropped blots.

### Chronological lifespan using methylene blue

All aging stationary phase cultures were initiated by seeding a single colony of yeast into 5 mL of YPD or SD medium for auxotrophic selection with overnight shaking at 30 °C. Cultures were resuspended the next day into fresh media (typically 5 to 15 mL) in flasks with a liquid-air ratio of 1:4, with incubation at 30 °C and vigorous shaking. When a smaller volume was needed, when using the C43-4 synthetic peptide, 2 to 5 mL cultures were maintained in 15 mL conical tubes or 14 mL snap tubes. Cells continued dividing until reaching peak cell density, resulting in a post-diauxic shift, typically after 1 to 2 d. Flasks were left shaking to maintain this condition for 1 to 2 wk, with aliquots removed every second day to measure cell viability using methylene blue (Bapat et al. 2006; Kwolek-Mirek and Zadrag-Tecza 2014). A total of 10 to 20 μL of cells were removed each time and mixed 1:1 with 0.1% (w/v) methylene blue solution [0.7 g sodium acetate trihydrate, 0.3 g glacial acetic acid, 50 mg methylene blue {Fisher Scientific; M291-25}, add water to 50 mL] before plating on a hemocytometer. Yeast cells contain an enzyme that reduces methylene blue; viable cells remain colorless, while dead yeast cells stain dark blue. Viability was calculated as:


$$\mathrm{Viability}\;[\text{\%}]=\frac{\frac{}{}(\mathrm{Total}\;\mathrm{counted}\;\mathrm{cells}-\mathrm{total}\;\mathrm{counted}\;\mathrm{dead}\;\mathrm{cells})}{\;\;\mathrm{Total}\;\mathrm{counted}\;\mathrm{cells}\;\times \;100}.$$

### Aging arrest assays

A total of 50 mL cultures were set up, with 6 mL collected every second day from the chronologically aging cells. Cells were resuspended in fresh YPD to allow cells to return to the cell cycle, in the presence of 15 μg/mL of nocodazole (Fisher Scientific; 48792810MG) to arrest cells in their first mitosis after being released from stationary phase. Cell cycle arrest was verified via microscopy, ensuring that budding cells were visible before proceeding (see Supplementary Fig. 3 for the budding index). The budding index was also determined. A total of 2 mL of this culture was collected for the M phase time point, and the remaining 4 mL was washed with Ultrapure water (18.2 MΩ-cm) before resuspending in fresh YPD medium, with the addition of new arresting agents. A total of 2 mL was aliquoted with 60 μM α-factor (GenScript; RP01002) to arrest cells in the first “G1” phase after mitosis or with 300 mM hydroxyurea (Fisher Scientific; AAA1083106) to arrest cells in the first “S” phase after mitosis. Cultures were left shaking for 2 h at 30 °C and spun down to pellet for lysate preparation. Lysates were prepared and separated by SDS-PAGE for Western blot analysis of protein abundance in each phase of the cell cycle for 8 d.

### Cycloheximide chase assay

Cycloheximide (CHX) inhibits ribosome function to observe intracellular protein stability or degradation (Schneider-Poetsch et al. 2010). Cells were inoculated in 5 mL of SD medium and grown overnight at 30 °C shaking. Overnight cultures were diluted to an OD_600_ of 0.5 in 6 mL of fresh SD medium with 15 μg/mL of nocodazole to arrest cells in mitosis (approximately 2 h). At the 2 h time point, the *t* = 0 sample was collected (samples arrested in mitosis). Samples were spun down and rinsed twice with Ultrapure water (18.2 MΩ-cm) before resuspending in fresh SD media with 10 μg/mL of CHX. Aliquots were collected every 30 min for 90 min, freezing each pellet immediately after harvesting. Lysates were prepared and separated by SDS-PAGE for Western blot analysis of protein abundance at each time point.

### Integration of peptide constructs into the genome

To incorporate the HA-TrxA-C13-3 or HA-TrxA-C43-4 peptide constructs directly into the genome, 2 sets of PCR reactions were performed using Phusion High–Fidelity DNA Polymerase (Thermo, F530S). Two primers (listed in Table 3) were used on the Trp+ pJG4-5 vectors, which have the HA-TrxA-C13-3 or HA-TrxA-C43-4 constructs cloned into the multiple cloning site. The first integration primer (#1) was designed with an 18 bp sequence upstream of the TrxA peptide sequence and included a 60 bp overhang with homology to the TRP1 promoter. The downstream primer (#2) contained 18 bp of the final TrxA gene including the stop codon, with another 60 bp overhang of homology 100 bp upstream of the LEU2 promoter. The next set of primers was designed for use with genomic DNA harboring the LEU2 gene. The first primer in this set (#3) shared 18 bp of homology 100 bp upstream of the LEU2 gene, with a 60 bp overhang of the final TrxA gene including the stop codon. The final primer (#4) shared 18 bp of homology to LEU2 from the stop codon and had a 60 bp overhang downstream of TRP1. The schematic shown in Supplementary Fig. 1 outlines the plan for the integration of these PCR fragments into the Clb1-TAP (YTH4990) strain that lacks a functional LEU2 gene (*leu2Δ0*). The integration fragments were used to incorporate a functional LEU2 gene with the peptide construct into the TRP1 locus, disrupting the TRP1 gene.

**Table 3.** List of primers used in this study.

Primer name	Sequence (5′-3′)	Function
**#1 TRP1 60 bp upstream with TrxA 18 bp start FWD**	5**′** CATTGGTGACTATTGAGCACGTGA	Integrate the HA-C43-4::TRP1 or HA-C13-3::TRP1 construct into LEU2 strain
	GTATACGTGATTAAGCACACAAAGGCAGCTTGGAGTACGTTAACTGCTCATTGC	
**#2 LEU2 100 bp upstream with TrxA 18 bp stop REV**	5**′** GAAATGTAAAAGGTAAGAAAAGTTA	Integrate the HA-C43-4::TRP1 or HA-C13-3::TRP1 construct into LEU2 strain
	GAAAGTAAGACGATTGCTAACCACCTATTGGAAAATTAGATGCAGCTAGCCAG	
**#3 LEU2 100 bp upstream with TrxA 60 bp stop FWD**	5**′** AGTGTTGCACTGGCTAAAGTCAGTG	Integrate the HA-C43-4::TRP1 or HA-C13-3::TRP1 construct into LEU2 strain
	AGAGTTCTCGAACGCTACTGGCTAGCTGCATCTAAAGGTGGTTAGCAATCGTC	
**#4 TRP1 60 bp downstream with LEU2 18 bp stop REV**	5**′** TCAAAAGGCCTGCAGGCAAGTGCAC	Integrate the HA-C43-4::TRP1 or HA-C13-3::TRP1 construct into LEU2 strain
	AAACAATACTTAAATAAATACTACTCAGTAATAACTTAAGCAAGGATTTTCTT	
**APC10 500 bp upstream FWD**	5**′** TGCCTCTCCTATTTGGAC	Incorporate APC10-TAP::HIS3 or *apc10Δ::KanMX6* allele
**APC10 500 bp downstream REV**	5**′** TGATACCATACGAAGGGG	Incorporate APC10-TAP::HIS3 or *apc10Δ::KanMX6* allele
**APC10 100 bp upstream FWD**	5**′** ATGTGCCACTGTAAGGAC	Incorporate APC10-TAP::HIS3 allele
**APC10 100 bp downstream REV**	5**′** ATGCGGCAAAATAAGCGC	Incorporate APC10-TAP::HIS3 allele

### Complex formation between FITC-labeled peptides and TAT2

To allow complex formation between C43-4^FITC^ and the membrane-permeable peptide TAT2 (peptides obtained from BioMatik), we first diluted 10 μL of a 10 μg/μL TAT2 solution in 90 μL water. A total of 5 mg of the powdered C43-4 peptide was then resuspended in 5 mL water, with 10 μL of that solution diluted with 90 μL of water. We then mixed 10 μL of the diluted TAT2 and peptide solutions, with 80 μL water added. Next, we incubated the mixture at room temperature for 15 min for complex formation. A total of 100,000 yeast cells were then pelleted, leaving 100 μL of supernatant. A total of 100 μL of the peptide/TAT solution was added, gently mixed, and then incubated for 15 min. Cells were then used for experiments.

### Nuclear fractionation in yeast

Nuclear fractionation was conducted to visualize the localization of the HA-TrxA-C43-4 plasmid-expressed peptide and the synthesized C43-4^FITC^ peptide. Yeast cultures were grown overnight at 30 °C in YPD, then pelleted and washed twice using spheroplasting buffer [1.2 M sorbitol {Sigma-Aldrich; S1876-500G}, 0.1 M KH_2_PO_4_ {Sigma-Aldrich; P5379-100G}]. The cells were resuspended in spheroplasting buffer with 0.2 mg/mL zymolyase (Amsbio, 120491-1) and incubated at 30 °C for 1 h to rupture the cell wall. Cells were monitored for spheroplasting using a 100× lens (Olympus CX41). The spheroplasted cells were then pelleted, washed, and resuspended in 1 M sorbitol, with the addition of NP-40 lysis buffer (20 mM Tris-HCl, pH 7.4, 20 mM NaCl, 10 mM MgCl_2_, 1 mM EDTA, 1% NP-40) containing protease and phosphatase inhibitor tablets (Sigma-Aldrich, 04693159001, PHOSS-RO). The cells were then left to lyse on ice for 15 min. The culture was centrifuged at 500 × *g* for 5 min at 4 °C. The protein-containing supernatant was then centrifuged at 13,000 × *g* for 15 min at 4 °C. The subsequent supernatant consisted of the cytoplasmic fraction, while the final pellet was resuspended in lysis buffer as the nuclear fraction. All samples were then sonicated (Branson Sonifier 250) at 30% duty pulse cycle for 5 pulses for additional lysis, with protein concentrations determined using a Bradford assay (Bio-Rad, 5000006). Samples were then heated at 80 °C for 5 min with 1× sample loading buffer [5× loading buffer {250 mM Tris-HCl, pH 6.8, 10% w/v SDS}, 25% v/v 2-mercaptoethanol, 50% v/v glycerol, and 0.5% w/v bromophenol blue].

### *Caenorhabditis elegans* strains

The following strains were used: N2 bristol wildtype, MWU96 *cwwIs3*[*eft-3p-TrxA(APC)::GFP; myo-2p::tdTomato*], MWU128 *cwwIs3*; *daf-16(mu86),* CF1038 *daf-16(mu86)*, MWU 143 *cwwIs3; aak-(ok524)*, RB755 *aak-2(ok524)*, MWU146 *cwwIs3; akt-1(ok525)*, and RB759 *akt-1(ok525)*. CF1038, RB755, and RB759 were outcrossed with N2 6 times prior to generating MWU128 and MWU143 for the lifespan assay. Worms were cultured at 20 °C using standard methods as described (Brenner 1974).

### *C. elegans* transgenic construction

To generate the C43.4::GFP strain, a synthetic DNA fragment containing the SV40 NLS:HA:TrxA:C43.4 sequence was first synthesized by GeneStrands (Eurofin Genomics) and fused with GFP using the PCR methods as described by Hobert (2002). The GFP fusion fragment was amplified with *attB1* and *attB2* overhangs and cloned using the Gateway method into the pDEST-eft-3p plasmid that drives ubiquitous gene expression, flanked by the *unc-54* 3′ UTR. The constructed plasmid was microinjected into *C. elegans* at 25 ng/µL along with the *myo-*2::tdTomato co-injection marker (2 ng/µL). Transgenic strains expressing the extrachromosomal array were then integrated to generate stable expression using the UV irradiation technique, followed by 6 rounds of outcrossing with the N2 wildtype prior to use.

### *C. elegans* lifespan assays

Lifespan assays were conducted as previously described (Tabarraei et al. 2023). *C. elegans* were synchronized using the hypochlorite treatment and grown on HT115 empty vector *Escherichia coli* as the food source at 20 °C. Worms were picked manually to segregate from the offspring during the reproductive period. Day 1 is considered the first day of adulthood. Three independent trials were performed for all lifespan assays, with statistics for each trial listed in Supplementary Tables 7 to 10. Lifespan data were analyzed using the log-rank test in OASIS2 software (Han et al. 2016, 2024).

### Statistical analyses

Statistical data calculations for RLS experiments were determined using OASIS2, as described previously (Gutierrez et al. 2026). Log-rank tests were applied. Area under the curve (AUC) calculations were performed for CLS experiments for each strain ± SEM and analyzed with a one-way ANOVA and Dunnett's multiple comparisons post hoc test using GraphPad Prism. Spot dilution normalization of the 100-fold dilution (third column) for each strain ± SEM was determined using a one-way ANOVA and Dunnett's multiple comparisons post hoc test, as described previously (Petropavlovskiy et al. 2020). One- or two-way ANOVA tests were used to compare means of unmatched groups. Multiple comparisons tests were performed post hoc, as indicated, to calculate *P*-values and assess significance between strains or treatments. Tukey's post hoc testing was used after one- or two-way ANOVA tests to identify which pairs of group means were different. Dunnett's post hoc testing was used to compare multiple treatment groups to a single control group. Šídák's post hoc testing was used to compare specific and independent group means. Significance is denoted as “ns” for nonsignificant, *P* < 0.05*, *P* < 0.01**, *P* < 0.001***, and *P* < 0.0001****. Values represent ±standard error of the mean (SEM).

## Results

### The APC loses function in aging quiescent yeast cells

Mutations to subunits of the APC exhibit short replicative (APC^Cdc20^- and APC^Cdh1^-dependent) and chronological (APC^Cdc20^-dependent only) lifespans (Harkness et al. 2004). Therefore, we tested whether wild-type (WT) APC^Cdh1^ loses function in aging quiescent yeast cells. This is important as once cells enter quiescence, they can accumulate damage that eventually leads to senescence, a major hallmark of aging. Therefore, if APC^Cdh1^ activation can prolong the health of quiescent yeast cells via maintenance of repair pathways (APC^CDH1^ is associated with DNA repair and maintenance of genome stability; García-Higuera et al. 2008; Wäsch et al. 2010; Greil et al. 2016; Ha et al. 2017; Harkness 2018), it is expected that APC^Cdh1^ activation may extend the metabolic activity of quiescent cells, thereby delaying senescence and aging. To test this idea, we grew WT yeast cells expressing endogenous TAP-tagged APC substrates (Clb1-TAP and Mps1-TAP) to stationary phase, a nondividing quiescent state, and then aged them chronologically. Clb1 and Mps1 are targeted by APC^Cdc20^ during mitosis and by APC^Cdh1^ during G1 (Baumer et al. 2000; Cui et al. 2010). As a control, we used TAP-tagged Sic1, which is targeted by the Skp, Cullin, F-box (SCF) ubiquitin ligase at the G1/S junction for degradation (Fig. 1a) (Orlicky et al. 2003). We ensured that the APC functioning in these replicating cells was aged by arresting chronologically aging cells in the first M and G1 phases they encountered after being released into fresh YPD media. If the APC loses function in aging cells, then the APC^Cdc20^ substrates would not be as effectively targeted and accumulate in the subsequent G1. On the other hand, Sic1, targeted by the SCF at the end of G1, should remain low during S phase in aging cells (Fig. 1b). We show that in our hands, Clb1-TAP and Mps1-TAP accumulate in the M phase in log phase cells and are degraded by G1, whereas Sic1-TAP is highest in G1 and removed at S, as expected (Fig. 1c). We grew cells expressing the 3 TAP-tagged proteins to stationary phase, which relies on APC^Cdh1^ (Skaar and Pagano 2008; Cappell et al. 2016), removed samples every other day for 8 d, and then allowed them to reenter the cell cycle in YPD liquid media supplemented with nocodazole. Once a mitotically arrested sample was removed, nocodazole was washed away with α-factor or hydroxyurea added to arrest the cells in G1 or S, respectively. The APC substrates Clb1 and Mps1 accumulated at mitosis in the aging cells, while Sic1 accumulated at G1 (Fig. 1d to f). We observed that both Clb1-TAP and Mps1-TAP accumulated over the 8-d time course in G1 as the quiescent cells chronologically aged (Fig. 1d and e), while protein levels in mitotically arrested cells remained stable. The G1:M ratio increased over time in aging cells (Fig. 1d and e), supporting our hypothesis that the APC loses function with age. Opposed to this, our results show that the Sic1-TAP levels remained low during S phase in the chronologically aging cells (Fig. 1f). This indicates that while the APC appears to lose function in aging cells, targeting of Sic1 by the SCF is not impaired with aging.

**Fig. 1.**
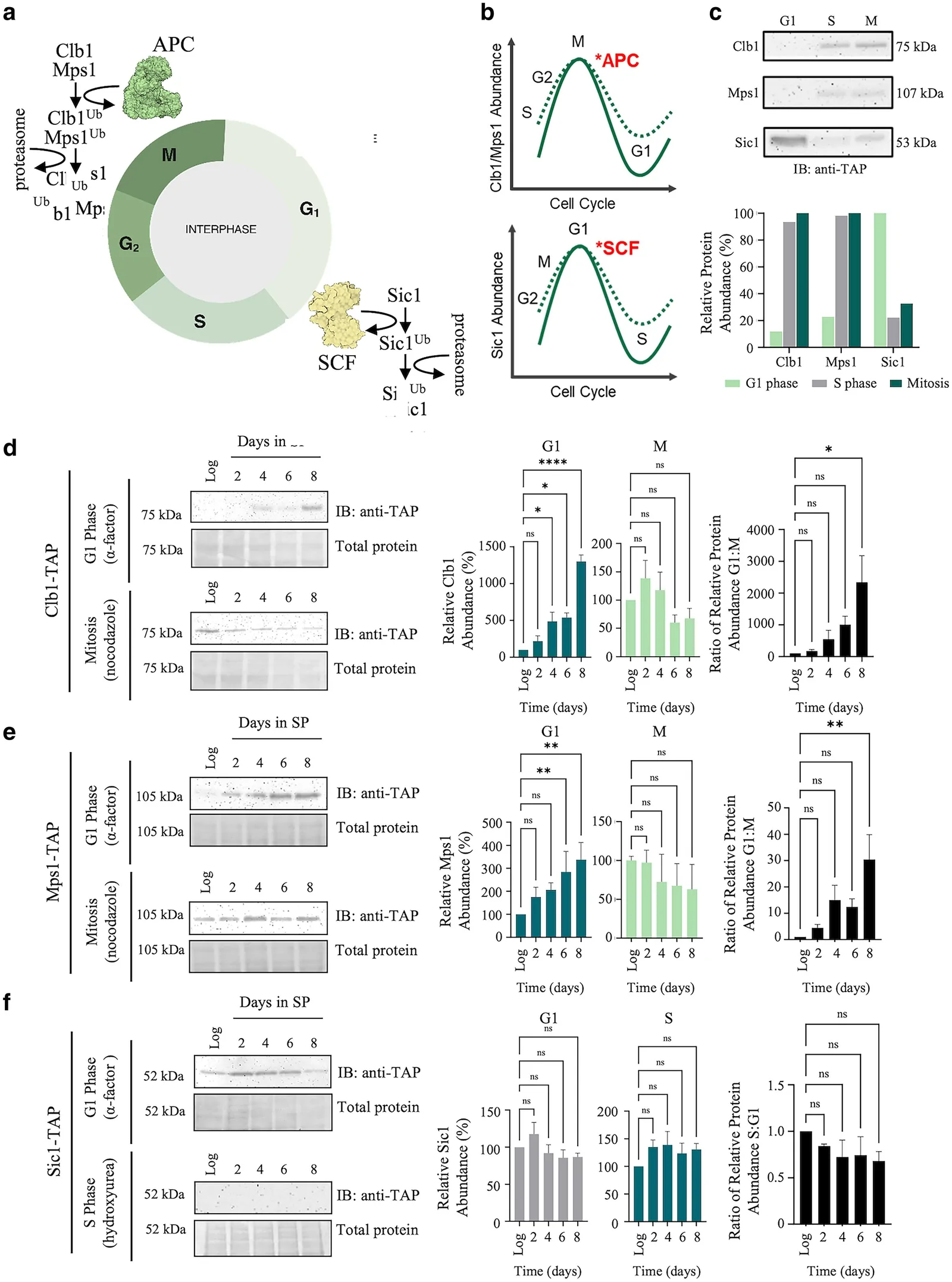
APC function decreases in aging cells. a) Schematic of APC and SCF activity during the cell cycle, with examples of substrates targeted for ubiquitination at these time points. b) Typical abundance of selected mitotic APC (top) or G1 SCF (bottom) substrates during the cell cycle in young, healthy cells when effectively targeted for ubiquitination. Dotted lines are expected changes in abundance of G1 APC (top) or S phase SCF (bottom) substrates during the cell cycle in chronologically aged cells when ineffectively targeted for ubiquitination. c) Representative western blot analyses on overnight samples of young, log-phase cells arrested with α-factor (G1), hydroxyurea (S), or nocodazole (M) to determine normal accumulation of proteins during the cell cycle. *n* = 1. Western blots were normalized to total protein load (Ponceau S-stained membranes) before each strain was normalized to M phase (100%), excluding Sic1-TAP, which was normalized to G1 (100%). Uncropped blots are shown in Supplementary Fig. 2. Western blot analyses were performed on lysates harvested from Clb1-TAP (d), Mps1-TAP (e), or Sic1-TAP (f) cells in log and stationary phase. The budding index is shown in Supplementary Fig. 3, and full uncropped blots are shown in Supplementary Fig. 4. Chronologically aging cells in stationary phase were resuspended in nutrient-rich media, supplemented with α-factor, hydroxyurea, or nocodazole to arrest cells in G1, S, or M phase, respectively, to determine trends in protein accumulation. Densitometry was used to normalize proteins to the Ponceau S-stained membranes to determine relative abundance in the indicated cell cycle stages and to determine G1:M ratios for Clb1 and Mps1 and S/G1 ratios for Sic1. Values are presented as mean ± SEM for *n* = 3 and were analyzed with a one-way ANOVA and Dunnett's multiple comparisons post hoc test, where *P* < 0.05*, *P* < 0.001***, and *P* < 0.0001**** between log phase and each day of stationary phase. Normalized graphs were used to compare relative ratios between cell cycle phases over time (black bar graphs, right), analyzed with a one-way ANOVA and Dunnett's multiple comparisons post hoc test, where *P* < 0.05*.

### Treatment of aging dividing and nondividing cells with the small chemical APC activator M2I-1 increases RLS but does not increase CLS

To further validate whether the APC is required for lifespan, we performed CLS assays using a methylene blue method to compare how long APC mutants can remain metabolically active during stationary phase. In all cases, the APC mutants *apc5^CA^*, *apc10Δ*, *cdc20-1*, and *cdh1Δ* had a significantly shorter CLS (Fig. 2a; stats shown in Supplementary Fig. 5a). We previously showed that APC subunit mutants had a short CLS and RLS (*apc5^CA^*, *apc9Δ*, *apc10Δ,* and *cdc26Δ*; see Table 1 for the strain list; Harkness et al. 2004). APC9, APC10, CDC26, and CDH1 are not essential genes and can be deleted (denoted by Δ), whereas APC5 and CDC20 are essential. The *apc5^CA^* mutant was isolated in a screen for chromatin assembly (CA) mutants (Harkness et al. 2002). The *apc5^CA^* allele contains a deletion of nucleotides 36 and 37, generating a novel amino acid sequence after amino acid 12, with a stop codon 18 amino acids later, producing a temperature-sensitive phenotype at 37 °C. When the *apc5^CA^* allele was TAP-tagged on the C-terminus, it resulted in a shorter, weakly expressed protein (Menzel, J. MSc thesis, 2014), indicating that the *apc5^CA^* phenotype was likely due to translational slippage. The *cdc20-1* allele was isolated by the Amon lab (Visintin et al. 1997). Here, we show that both APC coactivators, Cdc20 and Cdh1, are required for normal CLS (Fig. 2a).

**Fig. 2.**
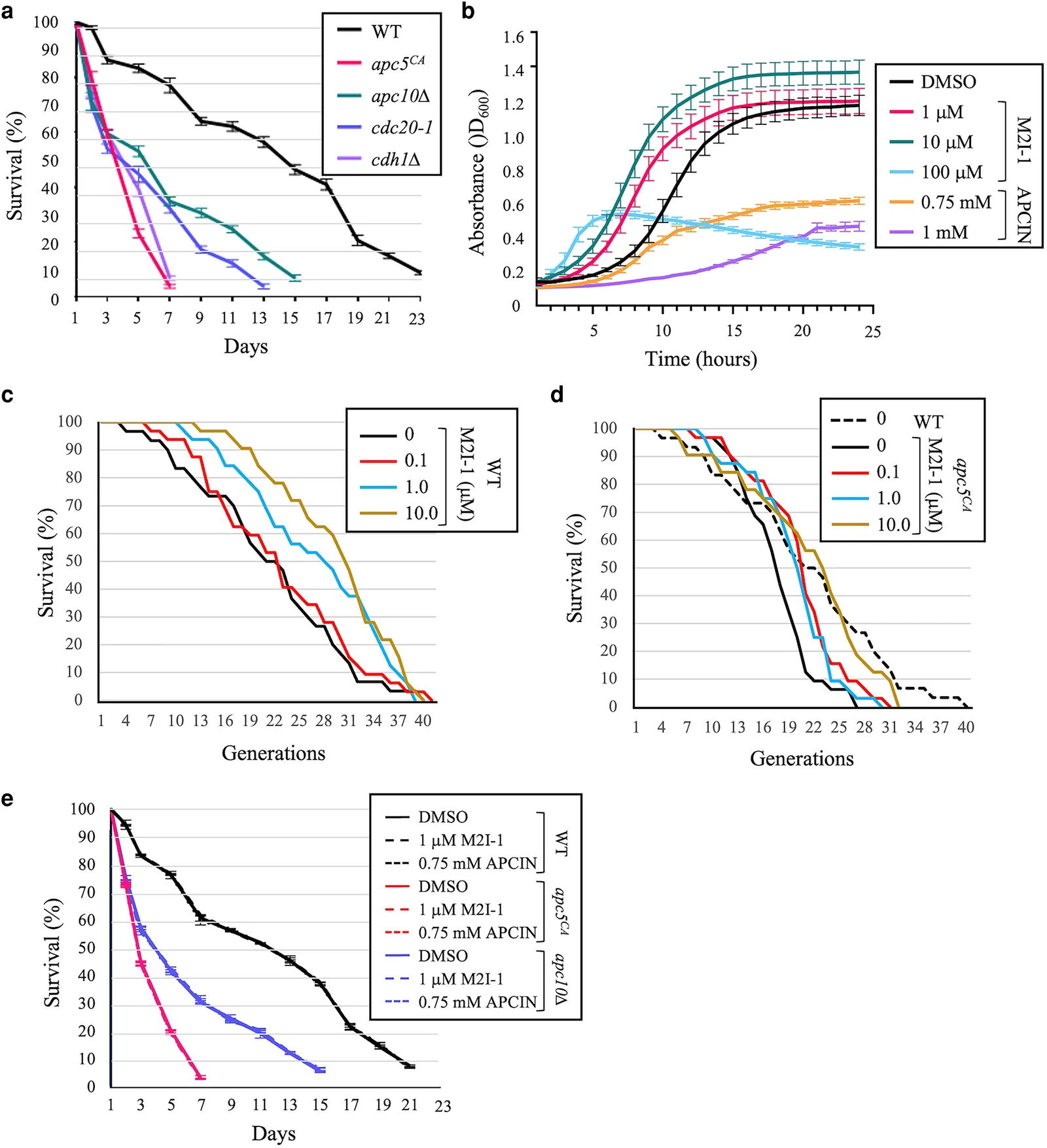
APC activation increases growth rate and RLS but not CLS. a) Chronological lifespan assays of WT (YTH2) and W303-based strains harboring APC mutants *apc5^CA^* (YTH5065), *apc10Δ* (4667), *cdc20-1* (YTH3863), and *cdh1Δ* (4670), compared in SD media. Survival was assessed using methylene blue cell counts every second day starting on Day 1 until survival reached <10%. Values are presented as mean ± SEM for *n* = 3. The quantitation is shown in Supplementary Fig. 5a, with statistical analyses presented in Supplementary Fig. 5b. The CLS for all APC mutants was significantly different from WT, with *P* < 0.0001****. b) 96-well growth assays were performed using YTH1 W303-based cells treated with the doses of the APC activator M2I-1 and the APC inhibitor APCIN shown, in SD media supplemented with 2% sucrose. 1% DMSO was used as the vehicle control, which corresponded to the concentration of DMSO in the 100 μM M2I-1 sample. The OD_600_ reading was measured every hour for 24 h. Values are presented as mean ± SEM for *n* = 4. Quantitation and significance are shown in Supplementary Fig. 5c. The growth differences in the presence of M2I-1 and APCIN were statistically significant, with *P* < 0.0001****. c) Replicative lifespan of WT (YTH2066) and d) *apc5^CA^* (YTH2078) cells exposed to the doses of M2I-1 shown. Cells were maintained on YPD plates supplemented with the appropriate M2I-1 doses. Statistical data are shown in Supplementary Tables 1 and 2. e) W303-based WT (YTH1), *apc5^CA^* (YTH5065), and *apc10Δ* (YTH4667) strains were treated with the indicated doses of M2I-1 or APCIN, grown in SD medium with 2% sucrose. 1% DMSO was used as the vehicle control. Cells were grown to stationary phase and then chronologically aged. Survival was assayed by methylene blue cell counts every second day starting on Day 1 until survival reached <10%. Values are presented as mean ± SEM for *n* = 3 for each strain and condition. The treatments had no effect on CLS; therefore, the overlap of the 3 CLS experiments for each strain looked like a single CLS experiment. Quantitation and significance are shown in Supplementary Fig. 5d. The M2I-1 and APCIN CLS curves were not significantly different from the controls.

To test if the lifespan defects in APC mutants are due to a loss of APC function, we treated cells with increasing doses of M2I-1, thereby prematurely activating the APC (Kastl et al. 2015). First, we determined if chemical APC activation or inhibition impacted the growth rate of cells. We observed that 1 and 10 μM M2I-1 increased the growth rate of cells, while the higher dose of 100 μM impaired cell growth (Fig. 2b; stats shown in Supplementary Fig. 5c). We predicted that if APC activation increased cell growth, then APC inhibition, using APCIN (APC inhibitor) (Sackton et al. 2014), a chemical that blocks the ability of Cdc20 to interact with the APC, should decrease the growth rate of cells. We observed that 0.75 and 1 μM APCIN impaired cell growth (Fig. 2b; stats shown in Supplementary Fig. 5c). These observations suggest that increasing APC activity using M2I-1 at 1 and 10 μM increases yeast growth rate.

To test the effects of M2I-1 on RLS, we maintained mother cells on plates with increasing doses of M2I-1 (0.1, 1.0, and 10.0 μM). We found that treatment of WT cells with M2I-1 increased the mean RLS by roughly 50% at the highest dose used (Fig. 2c; statistical analyses and *P*-values shown in Supplementary Table 1). Similarly, when the RLS of *apc5^CA^* cells was determined following increased exposure to M2I-1, it was observed that M2I-1 restored *apc5^CA^* short lifespan back to WT levels (Fig. 2d; statistical analyses and *P*-values shown in Supplementary Table 2). Next, we assessed whether M2I-1 could also increase the CLS of quiescent WT and APC mutants. Only APC^Cdh1^ is present in stationary phase cells (Skaar and Pagano 2008; Cappell et al. 2016), whereas both APC^Cdc20^ and APC^Cdh1^ are required in cycling cells. We observed that 1 μM M2I-1 had no effect on the CLS of the cells (Fig. 2e; quantitation shown in Supplementary Fig. 5d). APCIN also had no impact on the CLS of WT and mutant cells. This is consistent with both M2I-1 and APCIN only impacting cycling cells, through interactions with Cdc20, and not Cdh1 (Sackton et al. 2014; Li et al. 2019).

### Characterization of peptides that bind to Apc10

To identify a method to activate APC^Cdh1^, we characterized the phenotypes of peptides expressed from the *E. coli* Thioredoxin A (TrxA) backbone that bound to the APC subunit Apc10 using a 2-hybrid screen (VanGenderen et al. 2026; Fig. 3a; see Table 2 for plasmids used). One of these peptides, C43-3, resensitized drug-resistant breast cancer cells to therapy, both *in vitro* and *in vivo*. First, a select group of peptides was expressed in W303-based WT cells (Fig. 3b). The transformed cells were spot diluted onto SD -Trp media supplemented with either 2% sucrose or 1% galactose/1% sucrose to induce the TrxA-peptide constructs that were under the control of the GAL1 promoter and grown at 30 °C for 3 d. We chose to use sucrose as a noninducing agent, as this sugar has less impact on galactose promoter repression than glucose (Neumann and Lampen 1967; Johnston et al. 1994). We observed that when induced with 1% galactose, C9-5, C13-3, and C43-4 slowed the growth of cells (Fig. 3b; quantified in Fig. 3c). Next, we used a plate reader with cells growing in 96-well plates, where the OD_600_ was measured every hour for 24 h to assess whether the peptides shown above continued to slow growth in liquid media (Fig. 3d). Peptide C9-5 slowed the growth to a greater extent than C13-3 and C43-3 when expressed with 1% galactose. We prepared lysates from the cells and performed Western blots against the HA motif cloned into the TrxA-peptide construct. The peptides were all expressed, as expected (Fig. 3e). Peptides C9-5, C13-3, and C43-4 were cloned in-frame with TrxA, generating larger bands than C2-4B, which was not cloned in-frame, creating an early stop codon rather than the C-terminal half of TrxA.

**Fig. 3.**
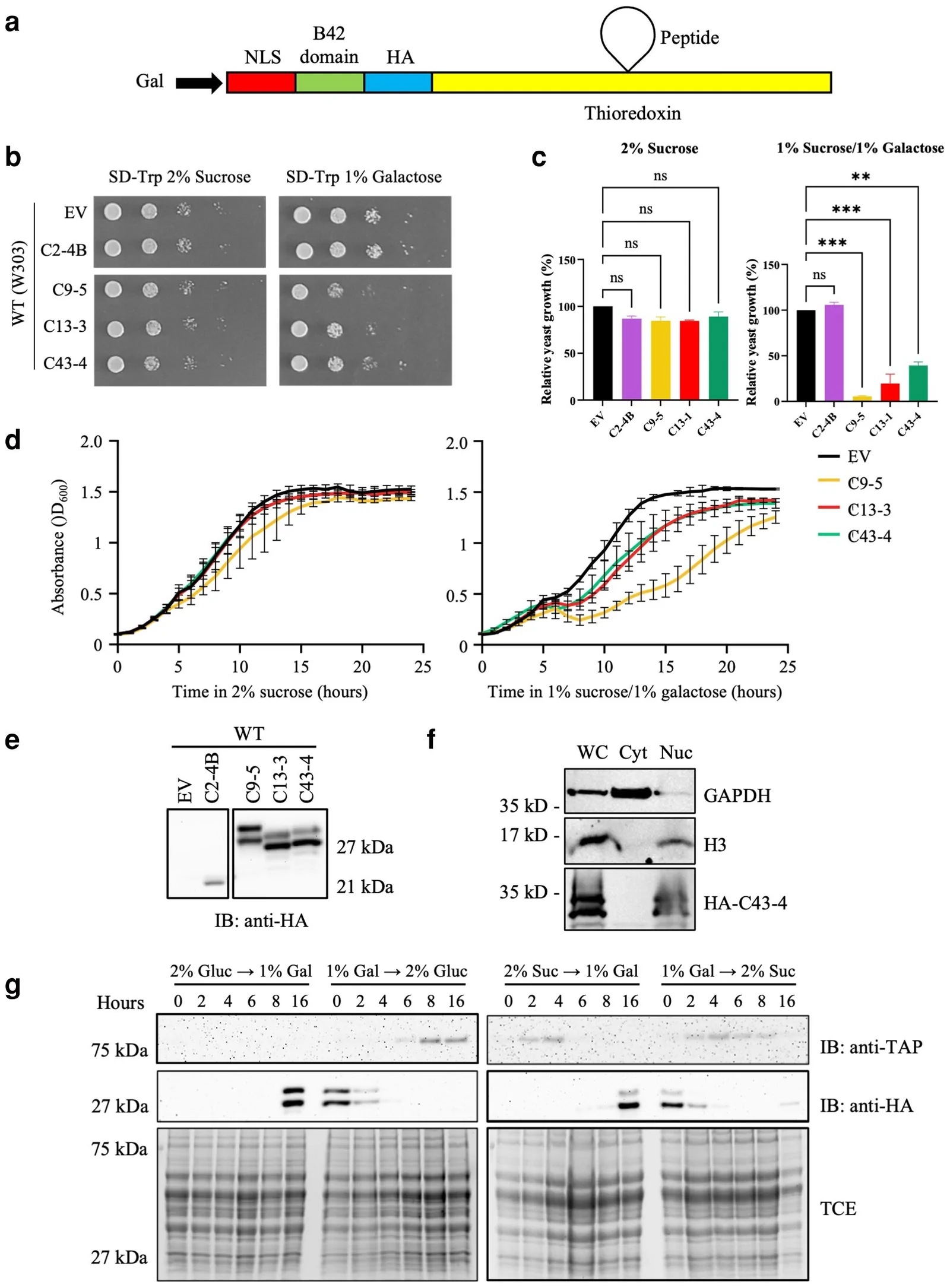
Phenotypic analyses following induction of the different peptide constructs. a) Structure of the Thioredoxin A (TrxA) peptide display cassette. The peptide was cloned into the TrxA backbone, which was fused to a nuclear localization signal (NLS), a transcriptional transactivation domain (B42), and an HA tag, all under the galactose-inducible promoter, as previously described (VanGenderen et al. 2026). b) Spot dilution assay for the WT W303-based strain (YTH1) transformed with the pJG4-5 EV and 4 different peptide constructs. Cells were cultured overnight, adjusted based on optical density, with 10-fold serial dilutions in 5 μl then spotted onto SD -Trp solid agar plates containing the indicated concentrations of carbon sources to induce peptide expression. Plates were incubated at 30 °C for 3 d before imaging. Two sets of independent cultures were tested, and selected representative images are shown for *n* = 2. c) Normalization of 100-fold dilution (third column) for each strain ± SEM was analyzed with a one-way ANOVA and a Dunnett's multiple comparisons post hoc test. No significant differences were observed between the EV control strains and peptides in 2% sucrose, whereas in galactose, the differences between the EV control and C9-5, C13-3, and C43-4 were significant; *P* < 0.01** and *P* < 0.001***. d) Growth curves of the YTH1 strain transformed with an EV control or the 4 different peptide constructs shown above, grown in 2% sucrose (left) or 1% sucrose/1% galactose (right) to induce peptide expression. Cells were cultured overnight, adjusted based on optical density, and maintained in a 96-well plate for OD_600_ readings every hour for 24 h using a microplate reader. Values are presented as mean ± SEM for *n* = 3. Curves were analyzed with a two-way ANOVA and a Dunnett's multiple comparisons post hoc test, where no significance was observed between strains in 2% sucrose and *P* < 0.01** between the EV and C9-5 peptide in 1% galactose/sucrose. e) Western blot analysis of HA-tagged peptide constructs to confirm yeast transformations; representative lysates harvested from cells grown in 1% galactose/sucrose are shown. Uncropped blots are shown in Supplementary Fig. 6a. f) Clb1-TAP cells expressing an integrated TrxA-HA-C43-4 (also contains an NLS) were grown overnight in 2% galactose to induce the cassette. Cells were fractionated into nuclear and cytosolic lysates. Whole cell (WC), cytosolic (Cyt), and nuclear (Nuc) fractions were assessed by Western blots using antibodies against GAPDH, histone H3, and HA. Uncropped blots are shown in Supplementary Fig. 6b. g) Western blot analyses of Clb1-TAP cells with galactose-inducible HA-TrxA-C43-4 integrated into the genome, grown initially overnight in 2% glucose (left) or 2% sucrose (right). The following day, samples were resuspended in 2% galactose (left) or 1% galactose/1% sucrose (right) supplemented media. Samples were collected every 2 h for 8 h, and another sample was taken the next morning after 16 h. Cells were then washed and resuspended in 2% glucose or 2% sucrose, with samples again taken every 2 h for 8 h, and the last sample taken after 16 h. Lysates were prepared for western blot analysis using anti-HA and anti-TAP to detect HA-TrxA-C43-4 and Clb1 abundance, respectively. Uncropped blots are shown in Supplementary Fig. 6c and d.

If the peptides do in fact activate the APC, then the peptides should be localized to the nucleus. We used HA-TrxA-C43-4 to determine whether the peptides could be localized to the nucleus. Therefore, we fractionated cells expressing HA-TrxA-C43-4 integrated into the genome of Clb1-TAP cells (see Supplementary Fig. 1 for the integration scheme) grown overnight in 2% galactose to recover nuclear and cytoplasmic fractions. We showed that histone H3 and GAPDH signals were exclusively in the nucleus and cytoplasm, respectively. As expected, HA-TrxA-C43-4 was exclusively in the nucleus (Fig. 3f). To confirm whether C43-4 activated the APC, we used Clb1-TAP cells with the integrated HA-TrxA-C43-4 cassette. Upon APC activation, we expect that Clb1-TAP protein levels in cells will decrease, as shown previously with chemical activation of the APC in human cells (Arnason et al. 2022; Lubachowski et al. 2024; VanGenderen et al. 2026). Cells were grown overnight in complete SD media supplemented with 2% sucrose, then washed and resuspended in 1% galactose/1% sucrose to induce C43-4, with samples taken as shown. The cells were once again washed and resuspended in 2% sucrose to slow C43-4 overexpression, and samples were again removed over time. We observed that C43-4 was expressed over time with galactose, with levels going down when galactose was removed (Fig. 3g). In support of C43-4 activating the APC, Clb1-TAP levels were reduced upon C43-4 induction and returned once C43-4 induction was stopped.

### C43-4 slows cell growth in a Cdh1-dependent manner

Consistent with increased APC activity slowing yeast growth, we previously reported that overexpression of APC5, or the FKH1 and FKH2 members of the APC stress response network, reduced yeast cell growth (Harkness et al. 2004; Postnikoff et al. 2012). We further characterized peptide effects on yeast growth by expressing C2-4B, C3-1, C3-1B, C4-3, C9-5, C13-3, and C43-4 in the WT W303-based genetic background (Supplementary Fig. 7a) or C2-4B, C9-5, C13-3, and C43-4 in the S288c-based genetic background (Supplementary Fig. 7b). The transformants were spot diluted onto solid SD -Trp media supplemented with either 2% glucose or 2% galactose. To test the effects of the peptides on heat stress, the plates were grown at 30 °C and 37 °C for 2 to 3 d and then scanned. C2-4B, C3-1, C3-1B, and C4-3 had little impact on glucose- or galactose-grown cells at 30° or 37 °C (Supplementary Fig. 7). C13-3, C9-5, and C43-4 slowed the growth of both W303- and S288c-based cells, with the effect more prominent with 2% galactose induction (compare spots shown in Supplementary Fig. 7a with those in Supplementary Fig. 7c; see Supplementary Fig. 7d for quantification of the spots shown in Supplementary Fig. 7c on 1% galactose). We speculate that C9-5, C13-3 and C43-4 may be increasing APC activity to high levels, which we have shown may slow cell growth (Harkness et al. 2004). This was also observed with M2I-1, which increased growth at lower doses but decreased it at higher doses (Fig. 2b).

To test whether the APC is required for the peptide slow growth phenotype, we transformed W303-based WT and *apc5^CA^* cells with the empty vector (EV) or TrxA-HA-C43-3 and spot diluted the cells onto SD -Trp plates supplemented with either 2% sucrose or 1% galactose/1% sucrose (Fig. 4a). C43-4 continued to slow the growth of *apc5^CA^* cells, but not as dramatically as observed in WT cells (Fig. 4a, quantified in Fig. 4b), supporting our observations that C43-4 requires a functional APC to assert its activity. To gain direct evidence that C43-4 requires either the Cdc20 or Cdh1 coactivators to activate the APC, we first transformed W303-based cells harboring a CDH1 deletion with C2-4B, C13-3, C43-4, and the EV control (Fig. 4c). The cells were spot diluted onto SD -Trp media supplemented with either 2% glucose or 2% galactose, with the plates grown at 30° or 37 °C. WT cells expressing C13-3 or C43-4 grew slowly when induced with 2% galactose, whereas C2-4B-expressing cells grew at the same rate as the EV control cells. The effect for C43-4 was enhanced when grown at 37 °C on 2% galactose plates (Fig. 4c; quantified in Fig. 4d). If Cdh1 was required for the peptide phenotypes, then we should no longer observe slowed growth when induced with 2% galactose in *cdh1Δ* cells. In cells expressing C13-3, growth remained significantly suppressed, indicating that Cdh1 does not affect C13-3 function. However, in *cdh1Δ* cells expressing C43-4, cell growth was largely restored. Opposed to C13-3, C43-4 appears to require Cdh1 for function. No substantial growth phenotype was observed for C2-4B, and thus we could not determine if it required Cdh1 for function. Next, we expressed the peptides shown in temperature-sensitive *cdc20-1* cells (Fig. 4e; quantified in Fig. 4f). We observed that with all the peptides tested, Cdc20 is required for activity, as all transformants grew to the same extent as the control cells expressing an empty vector when induced with galactose. It should be noted that growth on galactose suppressed the slow growth of *cdc20-1* cells observed on glucose. It has been described previously that glucose inhibits APC activity, while alternative carbon sources, such as glycerol, galactose, and raffinose, can suppress APC mutations (Baumer et al. 2000; Harkness et al. 2004). Taken together, these results support the idea that C43-4 enhanced both APC^Cdc20^ and APC^Cdh1^ activity. C13-3 appears to activate only APC^Cdc20^, with the remaining peptides requiring testing in future studies.

**Fig. 4.**
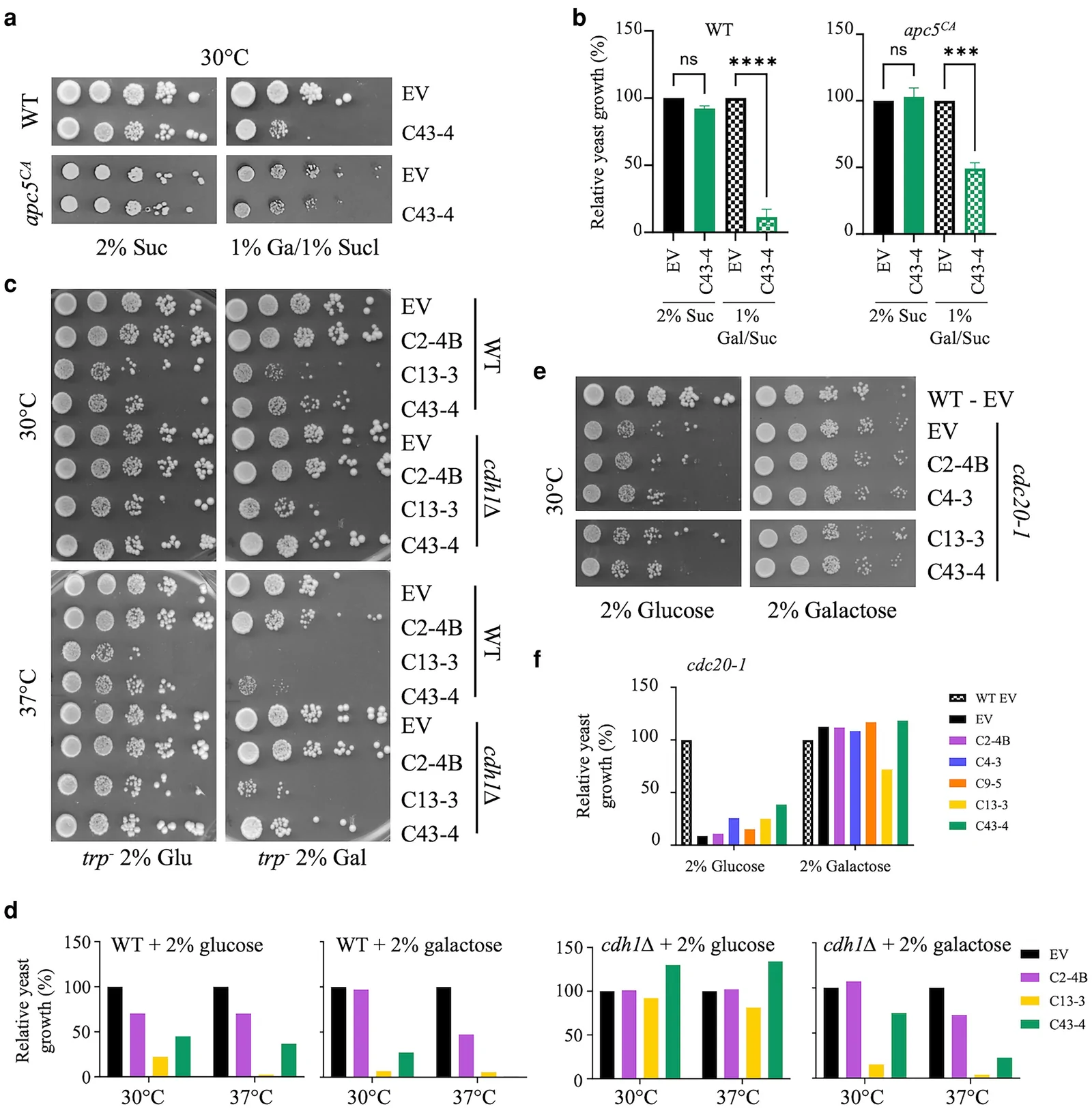
C43-4 growth phenotypes require both CDC20 and CDH1. a) WT (YTH1) and *apc5^CA^* (YTH5065) cells expressing either the EV or HA-TrxA-C43-4 were cultured overnight, adjusted based on optical density, then 10-fold serial dilutions were spotted onto SD -Trp plates supplemented with the sugars shown. Plates were incubated at 30 °C for 3 d before imaging. Two sets of independent cultures were tested, and selected representative images are shown for *n* = 2. b) Normalization of 100-fold dilution (third column) for each strain ± SEM was analyzed with a one-way ANOVA and a Dunnett's multiple comparisons post hoc test, where no significance was observed between the EV control strains and different peptides in 2% sucrose, and *P* < 0.001*** and *P* < 0.0001**** in 1% galactose. c) Spot dilutions of W303 WT (YTH1) and *cdh1Δ* (YTH4940) strains transformed with an EV control or 3 different peptide constructs were performed. Cells were grown in 2% glucose (left) or 2% galactose (right). The top panels were grown at 30 °C, while the lower panels were grown at 37 °C. Cells were grown for 3 to 5 d and then imaged. d) Normalization of 100-fold dilution (third column) for each strain was performed and plotted. *n* = 1. e) Spot dilutions of W303 WT (YTH1) cells transformed with an EV and *cdc20-1* (YTH3863) cells transformed with an EV or the 4 peptide constructs shown were performed. Cells were grown in 2% glucose (left) or 2% galactose (right) to induce peptide expression. Plates were incubated for 3 to 5 d and then imaged. f) Normalization of 100-fold dilution (third column) for each strain was performed and plotted; *n* = 1.

### APC-binding peptides increase the replicative lifespan of WT cells

We have previously shown that the APC is required for longevity in yeast (Harkness et al. 2004; Postnikoff et al. 2012; Menzel et al. 2014; Malo et al. 2016). Therefore, we predicted that peptide activation of the APC would increase yeast RLS, as M2I-1 did (Fig. 2c and d). We transformed the peptides shown into WT cells (YTH2066) for RLS (Fig. 5a; stats, mothers scored, and mean RLS shown in Supplementary Table 3). The cells were maintained on SD -Trp media supplemented with 2% glucose for low-level expression, as we have shown previously that 2% glucose results in low but detectable levels of expression from galactose-inducible promoters (Turner et al. 2010; Postnikoff et al. 2012; Menzel et al. 2014). Under these conditions, the mean RLS of WT cells expressing an EV or most of the peptides was shorter than normally observed on YPD (Supplementary Table 3). However, peptides C2-4B and C13-3 increased the mean RLS by ∼50% compared with that of control cells. We then tested the RLS of the cells maintained on YPD (Fig. 5b). The mean RLS of control cells was 20.87 ± 1.45 generations (Supplementary Table 4), as expected for YPD. Additionally, C2-4B continued to increase RLS, whereas C13-3 did not. The increase in RLS using C2-4B, however, did not reach significance (Supplementary Table 4). Utilizing the C43-4 peptide, we measured RLS when the peptide construct-expressing cells and controls were induced with YP 0.5% galactose/1.5% sucrose and compared with cells maintained on YP 2% glucose (YPD). We used cells expressing the HA-TrxA-C43-4 cassette that was integrated into the genome of Clb1-TAP cells (as used in Fig. 3f and g) to avoid the potential inconsistent effects of transforming plasmids. We observed that cells expressing integrated C43-4 induced with 0.5% galactose lived the longest (Fig. 5c; Supplementary Table 5). The mean RLS of WT + YPD was 22 ± 1.74 generations, while the mean RLS of WT + C43-4 + 0.5% galactose was 29.66 ± 1.97 generations, with a *P*-value of 0.0057. This indicates that C43-4 requires galactose induction to increase yeast RLS.

**Fig. 5.**
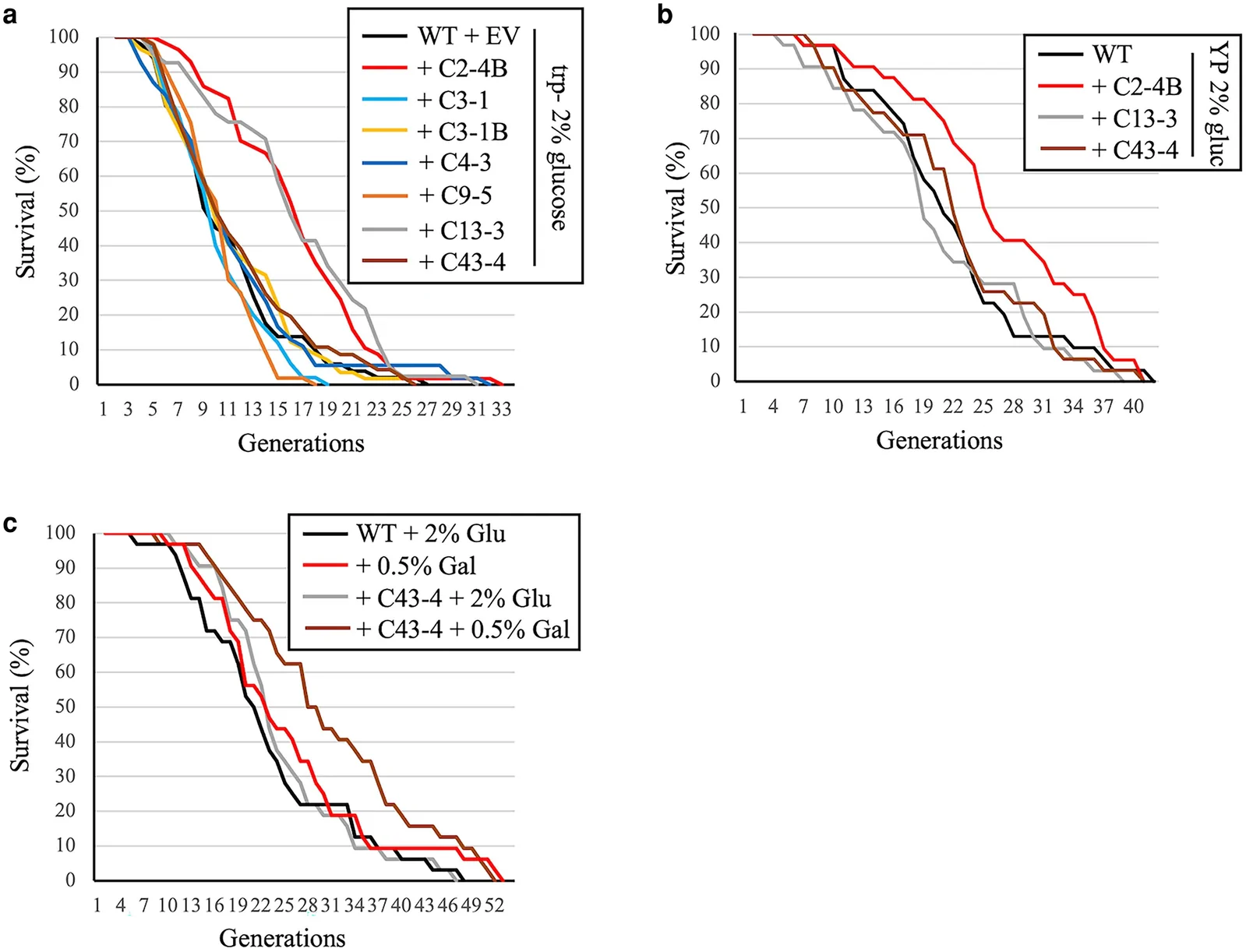
The peptides have different effects on RLS in cells. a) WT cells (YTH2066) were transformed with the peptides shown for RLS assays. Mother cells were placed on SD -Trp plates for the entirety of the experiment. Daughter cells were picked from each mother cell, counted, and discarded. Counting continued until the last mother stopped dividing. Statistical data were generated using OASIS2 and are shown in Supplementary Table 3. *P*-value for WT control vs WT + C2-4B was 9.4 × 10^−6^**** and for WT control vs WT + C13-3 was 2.5 × 10^−5^****. b) Peptides C2-4B, C13-3, and C43-4 were selected for further study. The Y2H strain (YTH3640), co-transformed with APC10-LEXA and the peptides shown, was used for RLS. Mother cells were placed on YPD for the entirety of the RLS experiment. Daughters were picked from each mother until all mothers stopped dividing. Statistical data are shown in Supplementary Table 4. c) Clb1-TAP cells and Clb1-TAP cells expressing integrated HA-TrxA-C43-4 were used for RLS in the presence of YPD or YP 0.5% galactose/1.5% sucrose. Statistical data are shown in Supplementary Table 5. The *P*-value for WT 2% glucose vs WT + C43-4 + 0.5% galactose is 0.0057**. All other comparisons were not significant.

### The C43-4 peptide increases CLS

RLS measures how many cell divisions a mother cell can go through prior to death, which is controlled by APC^Cdc20^ during mitosis and APC^Cdh1^ during mitotic exit and G1. To determine if the peptides can activate a function reliant on APC^Cdh1^ alone, we used CLS. CLS measures how long a population of quiescent cells can remain metabolically active. Entrance and maintenance of G0 are dependent upon APC^Cdh1^ activity (Skaar and Pagano 2008; Cappell et al. 2016). It is important to identify APC^Cdh1^ activators, as this version of the APC is associated with tumor suppression and maintenance of genome stability (García-Higuera et al. 2008; Wäsch et al. 2010; Greil et al. 2016; Ha et al. 2017). To the best of our knowledge, there are no known APC^Cdh1^ activators. As shown above, the commercial APC activator M2I-1 increased RLS, but did not increase CLS (Fig. 2c to e). This was not surprising since M2I-1 works specifically to release Cdc20 from the SAC and is therefore not expected to impact APC^Cdh1^-dependent activity, such as CLS. We tested cells expressing plasmid-borne peptides grown to stationary phase in SD -Trp media supplemented with either 2% sucrose or 1% galactose/1% sucrose for CLS. Peptides C2-4B, C9-5, and C13-3 did not increase CLS when induced, while C43-4 did (Fig. 6a to d; AUC calculations shown to the right of each panel; mean and max lifespans and 95% confidence intervals shown in Supplementary Table 6; all samples were done at the same time with the same WT control). The HA-TrxA-C43-4 cassette was cloned into a 2μ *URA3* plasmid, which also increased CLS in WT cells (Fig. 6e). We next assessed the CLS of Clb1-TAP cells harboring integrated copies of HA-TrxA-C13-3 and HA-TrxA-C43-3. Only the C43-4-expressing cells, when induced with 1% galactose/1% sucrose, exhibited increased CLS (Supplementary Fig. 8). We then assessed CLS in response to increased galactose concentrations. Using Clb1-TAP cells ± the integrated HA-TrxA-C43 cassette, we found that the CLS was unaltered by carbon source usage in cells lacking C43-4 (Fig. 6f; AUC quantified on the right). However, in the presence of C43-4, the CLS increased proportionally to the galactose concentration (Fig. 6g; AUC quantified on the right). Lastly, W303-based WT (YTH1), *apc5^CA^* (YTH5065), and *apc10Δ* (YTH4667) cells were transformed with the HA-TrxA-C43-4 plasmid, followed by CLS using MM SD -Trp media supplemented with either 2% sucrose or 1% galactose/1% sucrose (Fig. 6h; quantitation and mean, max, and stats shown in Supplementary Fig. 9). We observed that while C43-4 induction increased the CLS of WT cells (note that CLS is longer in SD media compared with MM; compared with the CLS in MM in Fig. 6a with CLS in SD media in Fig. 6h), it did not increase the CLS of the APC mutants tested. The results shown here indicate that C43-4 is the only peptide, expressed from the TrxA cassette, to increase CLS in an APC-dependent manner.

**Fig. 6.**
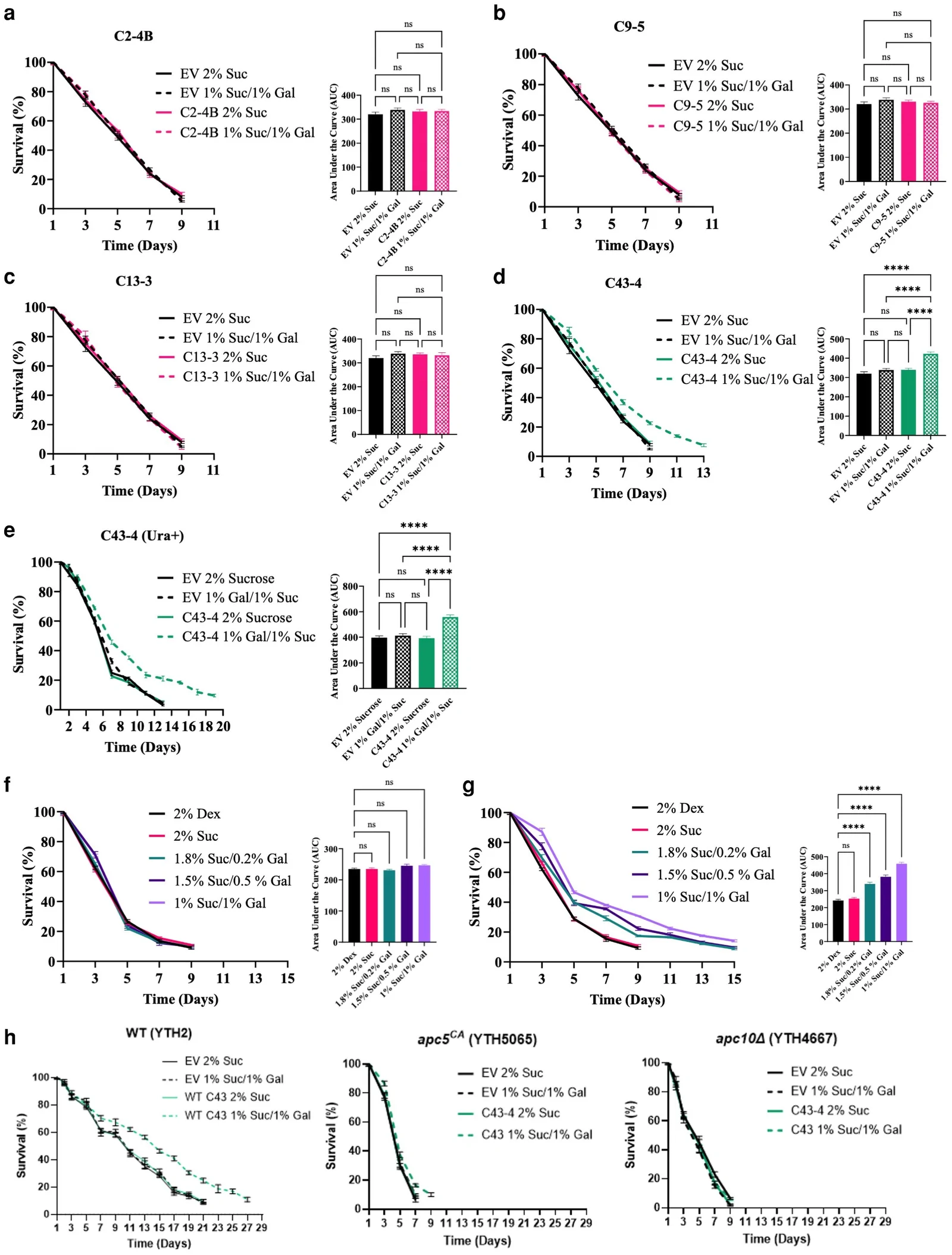
Only C43-4 increases CLS. CLS assays using W303-based cells transformed with the +Trp pJG4-5 EV control and 4 peptide constructs: a) C2-4B, b) C9-5, c) C13-3, and d) C43-4, grown in MM -Trp media with 2% sucrose or 1% galactose/sucrose. Values are presented as mean ± SEM for *n* = 2. e) CLS assays using WT S288c-based cells (YTH4504) transformed with the Ura+ pRS416 EV or the pRS416*-*C43-4 peptide construct in depleted MM -Ura media. Survival in the experiments above was assayed by methylene blue cell counts every second day starting on Day 1 until survival reached <10%. Values are presented as mean ± SEM for *n* = 3. Area under the curve (AUC) calculations for each strain ± SEM were analyzed with a one-way ANOVA and Tukey's multiple comparisons post hoc test where *P* < 0.0001**** between strains containing the C43-4 peptide in galactose and the control strains. Graphs are shown in the panels to the right. Mean and max lifespans and 95% confidence intervals are shown in Supplementary Table 6. f) CLS experiments with Clb1-TAP (YTH4990) or g) Clb1-TAP cells with integrated HA-TrxA-C43-4 (YTH5123) were used for CLS experiments. CLS on sucrose was compared with CLS on glucose (Dex). AUC quantitation is shown to the right. h) CLS was performed on W303-based WT (YTH1), *apc5^CA^* (YTH5065), and *apc10Δ* (YTH4667) cells using the methylene blue method, when maintained in depleted SD -Trp media. Quantitation and stats are shown in Supplementary Fig. 6.

### The APC-binding peptide C43-4 increases APC activity in chronologically aging cells

At the beginning of this study, we showed that the APC loses activity as cells age (Fig. 1). To test whether APC activity can be enhanced in quiescently aging cells, we used plasmid-borne HA-TrxA-C43-4 and the EV as a control, transformed into Clb1-TAP BY4741 cells, as done above (Fig. 1). Cells were removed on the days shown and allowed to reenter the cell cycle in the presence of nocodazole, then arrested in G1 using α-factor (budding index shown in Supplementary Fig. 10). We expected that aging cells reentering the cell cycle would have to rely on the activity of the APC currently in the aging cells. If the APC is impaired, then targeted degradation of Clb1-TAP at G1 would be deficient, leading to Clb1-TAP levels rising in G1 as cells aged (Fig. 7a, first panel from the left; increased G1:M ratio, rightmost panel). We observed this accumulation in cells expressing the EV in glucose (Fig. 7a), similar to Fig. 1d. Cells expressing the EV maintained in 2% galactose showed the same effect (Fig. 7b), with the G1:M ratio increasing as cells aged (Fig. 7b, rightmost panel). When cells expressing C43-4 were maintained in glucose while aging, we again observed a rise in the G1:M ratio (Fig. 7c; G1:M ratio shown in rightmost panel), indicating that the APC remains impaired while aging when C43-4 is not induced. However, when we maintained C43-4-expressing cells under inducing conditions, we observed that Clb1-TAP G1 levels were low throughout the aging experiment (Fig. 7d; G1:M ratio shown in rightmost panel). C43-4 abundance increased in aging cells under inducing conditions, indicating that C43-4 remains inducible throughout the aging experiment. The low G1:M ratio indicates that in the presence of induced C43-4, the APC remains active.

**Fig. 7.**
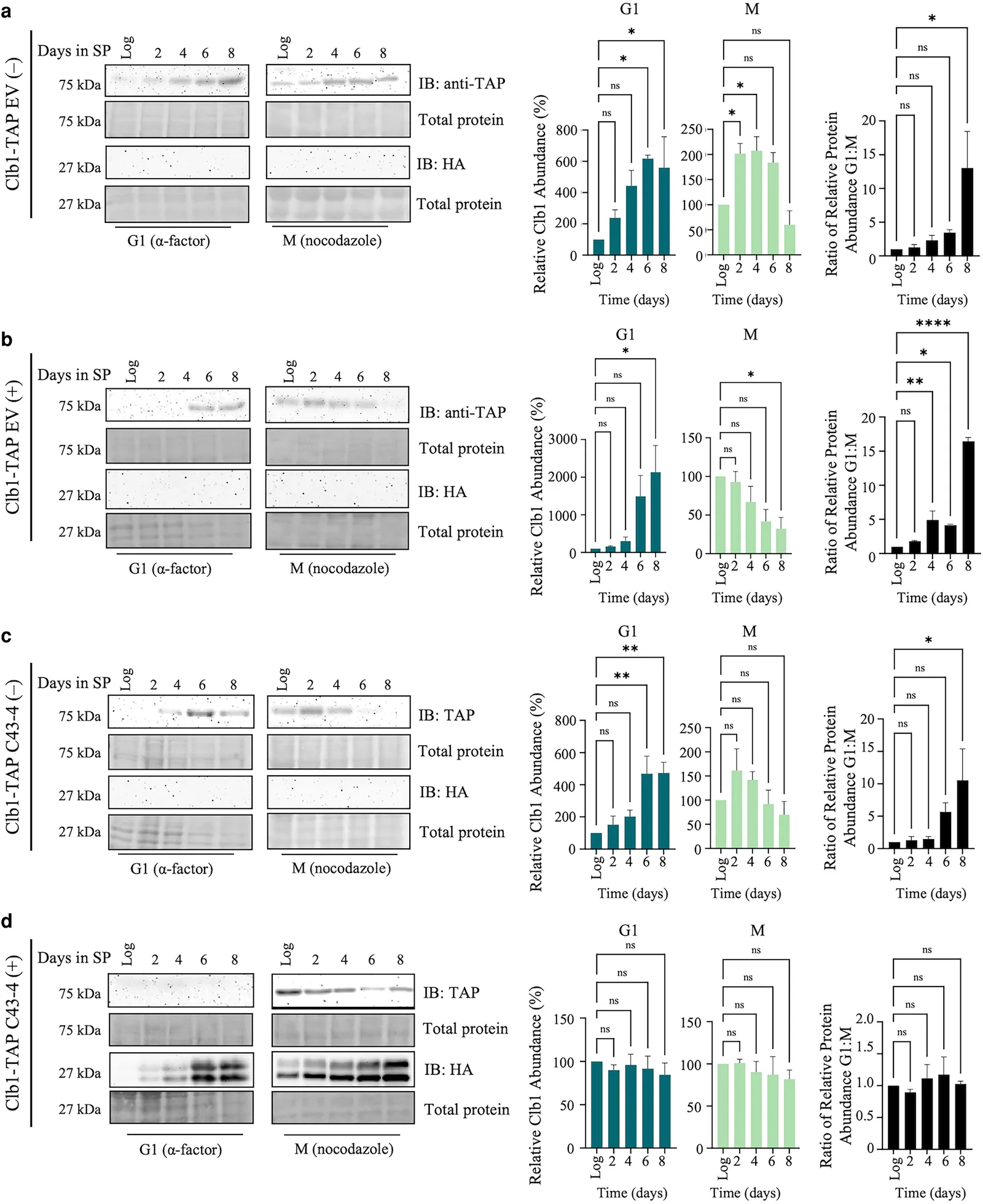
Degradation of APC substrate Clb1 is increased in aging cells upon APC activation by the C43-4 peptide construct. Western blot analyses were performed on lysates harvested from Clb1-TAP strains containing an EV or the integrated HA-TrxA-C43-4 construct in log and stationary phase over 8 d, maintained in depleted MM -Trp media with 2% glucose (−; a and c), or 2% galactose (+; b and d), to induce C43-4 expression. On the days indicated, samples were resuspended in nutrient-rich media to reenter the cell cycle in the presence of nocodazole or α-factor to arrest cells in mitosis or G1, respectively. Full uncropped blots are shown in Supplementary Fig. 11. Densitometry was used to normalize proteins to the total protein Ponceau S stains to determine relative abundance in the indicated stage of the cell cycle in aging cells between M and G1. Normalized graphs were used to compare relative G1:M ratios over time (black bar graphs, right). Values are presented as mean ± SEM for *n* = 3 and were analyzed with a one-way ANOVA and Dunnett's multiple comparisons post hoc test, where *P* < 0.05*, *P* < 0.01**, and *P* < 0.001*** between log phase and each day of stationary phase.

To get a view of whether the C43-4 peptide can be used therapeutically to activate APC^Cdh1^, we synthesized the 20 amino acid C43-4 fused to an FITC motif (C43-4^FITC^) and a scrambled control, also fused to FITC (scrambled^FITC^). We tested whether C43-4^FITC^ required a carrier peptide to cross the cell wall and plasma membrane. We incubated C43-4^FITC^ and scrambled^FITC^ with the cell-permeable TAT2 peptide, which consists of 2 repeats of the membrane-penetrating sequence RKKRRQRRR. The TAT2 cell-penetrating peptide is derived from the HIV-1 transactivator protein (Torchilin 2008) to allow complex formation. The TAT2 peptide has been observed to enter yeast cells, but the efficiency is far less compared with mammalian cells due to the yeast cell wall (Isogai et al. 2025). We performed CLS in BY4741 cells with 5 μg/mL C43-4^FITC^ or scrambled^FITC^ added to the growth media at the beginning of culturing, in the presence or absence of 5 μg/mL complexed TAT2. The results show that C43-4^FITC^ increased CLS in the presence or absence of TAT2 (Fig. 8a). Neither the addition of TAT2 alone nor the addition of scrambled peptide influenced CLS. This confirmed that the C43-4 peptide sequence, and not the TrxA backbone, is necessary for extending lifespan. Next, we added increasing amounts of C43-4^FITC^ or scrambled^FITC^ in the absence of TAT2 to the cell media. We added 1, 5, or 10 μg/mL peptide at the beginning of culturing or added 5 μg/mL at the beginning of culture and again at Day 1 of stationary phase. We observed that increasing the amount of scrambled^FITC^ had no effect, but increasing C43-4^FITC^ steadily increased the maximum CLS of cells (Fig. 8b). It is noteworthy that a single C43-4^FITC^ dose at the beginning of the experiment is sufficient to increase CLS. Also, the addition of 5 μg/mL at the beginning and again upon entering stationary phase was equivalent to adding 1 dose of 10 μg/mL at the beginning of the experiment, indicating that C43-4 aids both dividing and nondividing cells. We were next interested in determining if C43-4^FITC^ could increase the CLS of already aging cells. For this experiment, 5 μg/mL C43-4^FITC^ was added either at the beginning of culturing or at Days 1, 3, 5, or 7 of stationary phase (Fig. 8c). The greatest effect on lifespan was observed when adding C43-4^FITC^ to the mitotically growing cells prior to entry into stationary phase. As can be seen in Fig. 8c, the CLS also increased when C43-4^FITC^ was added to aging cells, even at Day 7. This suggested that even later in stationary phase, lifespan could still be extended, and that APC impairment with age can be reversed. To determine whether C43-4^FITC^ required either of the APC coactivators, Cdc20 or Cdh1, we added C43-4^FITC^ to W303-based WT, *cdc20-1*, or *cdh1Δ* cells at the beginning of culture (Fig. 8d). We observed that both *cdc20-1* and *cdh1Δ* cells had a short CLS, as observed in Fig. 2a. C43-4^FITC^ increased the CLS of W303 WT cells but could not increase CLS in the mutant backgrounds, indicating that C43-4 requires Cdc20 and Cdh1 to function as a CLS extender.

**Fig. 8.**
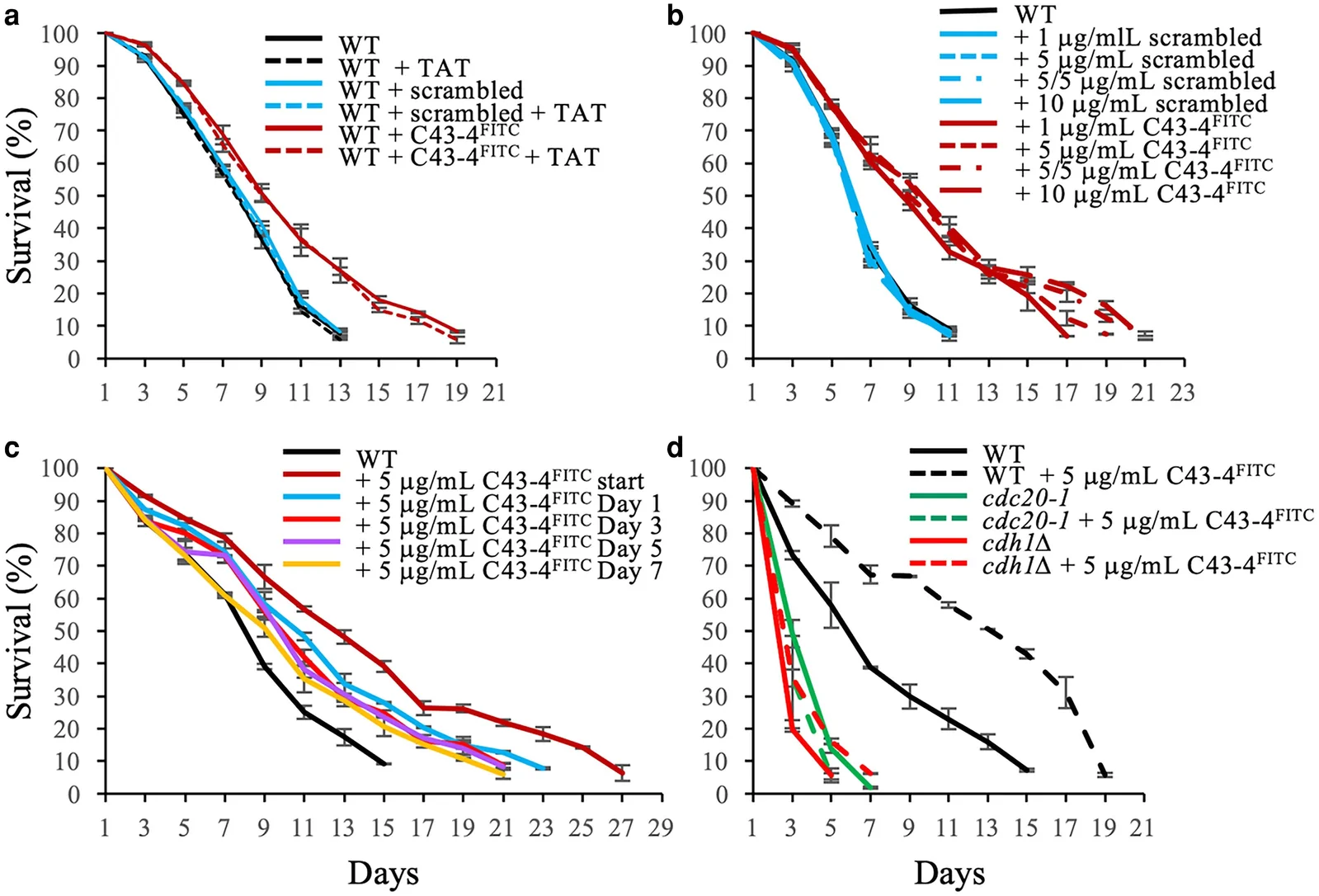
A synthesized 20 amino acid C43-4 fused with FITC (C43-4^FITC^) increases lifespan. a) WT BY4741 cells (YTH4504) were grown in the presence or absence of 5 μg/mL synthetic C43-4^FITC^. Scrambled peptide fused to FITC and the TAT2 permeable peptides were used as controls. Cells were grown to stationary phase, with samples removed every other day to assess viability using methylene blue to identify dead cells. b) BY4741 cells were grown to stationary phase in the presence of 1, 5, or 10 μg/mL C43-4^FITC^ added to the culture at the beginning of growth. In another sample, 5 μg/mL C43-4^FITC^ was added at the beginning and again when cells stopped dividing. Scrambled peptide was used in the same manner. Cell survival was determined using methylene blue. c) BY4741 cells were grown to stationary phase. C43-4^FITC^ was added at the beginning of growth, and then at the days shown in different samples while the cells were chronologically aging. Cell survival was assessed, as above, with methylene blue. d) W303-based WT, *cdc20-1*, and *cdh1Δ* cells were grown to stationary phase and aged chronologically, in the presence or absence of C43-4^FITC^. Survival was assessed using the methylene blue method. Error bars represent mean ± SEM; *n* = 3.

To confirm that C43-4^FITC^ was reaching the nucleus, we used an antibody that recognized the FITC motif. First, we observed that C43-4^FITC^ was present in whole cell lysates following overnight incubation and multiple washes, but the scramble peptide was not (Supplementary Fig. 12a; full uncropped blots are shown). To ensure the scrambled peptide could be recognized by the FITC antibody, we spiked lysates with 1 μg C43-4^FITC^ or scrambled^FITC^. We observed that both FITC-labeled peptides could be recognized by the antibody (Supplementary Fig. 12b). We then complexed the scrambled peptide with TAT2 and added this to cells overnight. We still did not observe scrambled^FITC^ in cells (Supplementary Fig. 12c). As observed with CLS, where the TAT peptide was not required to extend C43-4-dependent CLS (Fig. 8a), the TAT carrier peptide was not required for the translocation of C43-4 across the plasma membrane (Supplementary Fig. 12c). Using lysates fractionated into cytosolic and nuclear fractions, we observed that C43-4^FITC^, like HA-TrxA-C43-4, did indeed enter the cell and the nucleus (Fig. 9a, uncropped blots shown in Supplementary Fig. 12d). However, since the scrambled peptide did not enter the cell, we required a control peptide that entered the cell and nucleus but did not increase CLS.

**Fig. 9.**
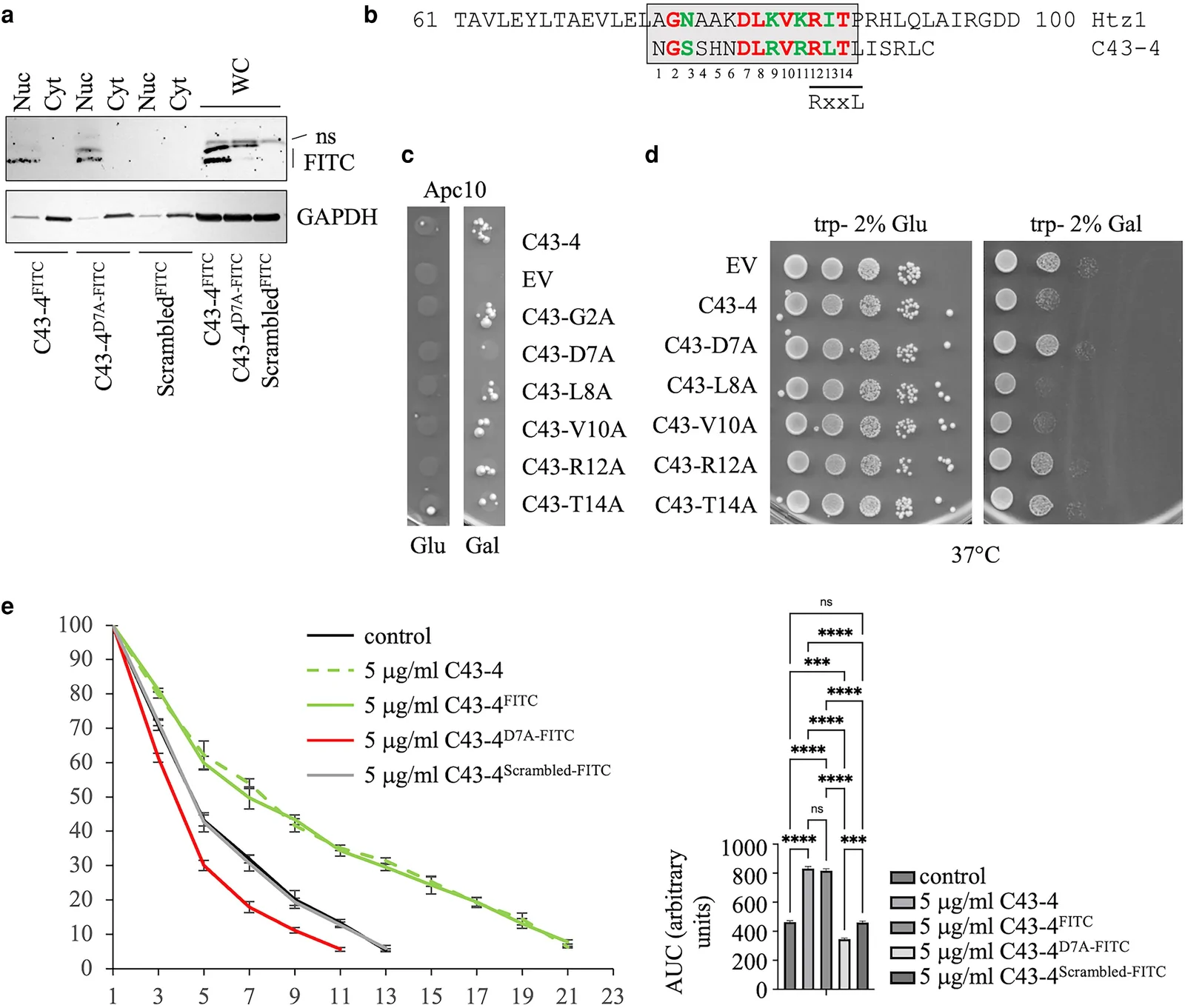
Identification of amino acids required for C43-4 function. a) Cells incubated with C43-4^FITC^ or untreated control cells were fractionated into cytosol and nuclear fractions. Antibodies against FITC, and GAPDH were used. C43-4, a mutated C43-4^D7A^, and the scrambled peptide were incubated with cells overnight. Full uncropped blots are shown in Supplementary Fig. 12d. b) Homology of C43-4 with a region of Htz1, with shared amino acids shaded in red and amino acids of the same class shaded in green. The RxxL motif is noted. The amino acids shaded in red in C43-4 were mutated to alanine. c) A 2-hybrid test using the mutated C43-4 constructs and LexA-Apc10. Cells were spotted onto -Trp/His/Ade supplemented with either 2% glucose or galactose. d) The C43-4 mutants were expressed in W303-based cells and grown at 37 °C on media containing 2% glucose or 2% galactose. The plates were grown at 37 °C for 2 to 3 days. e) The peptides shown were added to BY4741 cells at the beginning of the CLS experiment. The cells were maintained in depleted CM for the duration of the experiment. Counts using methylene blue staining were stopped once the cells alive were below 10%. Three biological repeats were performed for each sample, with mean ± SEM shown. Area under the curve (AUC) was calculated for each curve and plotted.

To generate a control for the synthetic C43-4 peptide, we mutated C43-4. In order to determine which amino acids to mutate, we performed a BLAST search of the peptide amino sequence against the yeast proteome. We observed that C43-4 shared sequence with a number of yeast proteins (Supplementary Fig. 13a). C43-4 encoded the D-box RxxL motif, which is observed in many APC substrates (Arnold et al. 2015). Proteins encoding the RxxL motif are often recruited to the APC by interacting with the Apc10-Cdc20/Cdh1 co-receptor (Carroll et al. 2005; da Fonseca et al. 2011). C43-4 shared sequence homology with the proteins Cst9 and Rpt1, both of which express an RxxL, but are not known to serve as APC targets. We chose Htz1, a stress-responsive histone H2A variant (Srivatsan et al. 2018; Saatchi and Kirchmaier 2019; Brewis et al. 2025) as our target, as we previously demonstrated that the APC interacts genetically with histones during mitosis (Harkness et al. 2002, 2005; Turner et al. 2010; Islam et al. 2011). Expression of galactose-inducible GST-HTZ1 (kind gift from A. Emili, U of Toronto) in WT, *apc5^CA^*, and *apcΔ10* cells, even at low levels in glucose, showed that HTZ1 could suppress APC mutant defects (Supplementary Fig. 13b). Since Htz1 is described as stress-responsive, we asked if GST-Htz1 protein levels are also responsive to APC activity. GST-HTZ1 expressed in the mutants shown above was grown in 0.5% galactose for 16 h to synthesize GST-Htz1. The cells were then arrested in mitosis with nocodazole, followed by treatment with CHX to inhibit the ribosome. Samples were taken every 30 min for 90 min (Supplementary Fig. 13c). Antibodies against Clb2, an APC substrate, were used as a control. We observed that Clb2 levels were elevated and stable in *apc5^CA^* and *apc10Δ* cells, as expected. Htz1 levels were also elevated in *apc5^CA^* cells and more so in *apc10Δ* cells. Htz1 was partially stabilized in the APC mutants, indicating that the APC, as well as another E3, likely controls Htz1 protein levels. Lastly, we asked if Htz1 levels may impact the protein levels of APC subunits. We expressed GST-HTZ1 in Apc5-TAP and Apc10-TAP cells. As a control, we expressed a vector expressing only GST. Cells were grown overnight in glucose and then switched to 0.5% galactose-supplemented media, with samples recovered at 0, 8, and 16 h for Western analyses. We observed that Apc5-TAP levels were unchanged throughout the experiment, whereas Apc10-TAP was weakly expressed at the beginning of galactose induction and decreased after 16 h in control GST cells (Supplementary Fig. 13d). However, in the presence of GST-Htz1, Apc10-TAP levels increased over the time course. This suggests that Apc10 is an unstable protein that is stabilized by Htz1. This provides a potential mechanism whereby C43-4 may mimic Htz1 activity by interacting with the APC, possibly stabilizing Apc10, and promoting stress-responsive APC activity.

We therefore mutated all amino acids in C43-4 that were shared with Htz1 individually to alanine (amino acids shaded in red in Fig. 9b). We then expressed the mutant versions of C43-4 in yeast 2-hybrid cells co-expressing LexA-Apc10 to determine if any of the mutations disrupt the C43-3-Apc10 2-hybrid interaction. We observed that all mutant peptides, except C43-4^D7A^, still interacted with Apc10 (Fig. 9c). Next, we spot diluted W303-based cells expressing the C43-4 mutants on SD -Trp media at 37 °C supplemented with either 2% glucose or 2% galactose. We observed that the WT C43-4 peptide slowed the growth of cells, as did the L8A and V10A variants (Fig. 9d), indicating that L8 and V10 are not required for C43-4 function. The D7A, R12A, and T14A mutants all grew like the EV control, indicating that these amino acids are required for function. Notably, D7 is required for the 2-hybrid interaction, while R12 and T14 make up the RxxL domain. We therefore synthesized the C43-4^D7A^ peptide fused to FITC. When C43-4^D7A^-^FITC^ was added to cells overnight, we observed that the peptide was localized to the nucleus following cell fractionation (Fig. 9a). We then added the peptide to cells at the beginning of log growth for CLS studies. We observed that C43-4^D7A^-^FITC^ did not increase CLS but slightly reduced it (Fig. 9e). Therefore, the D7A mutation disrupts Apc10-peptide interactions, C43-4 function, and abolishes extended CLS, supporting our observations that C43-4 interacts with the APC and prolongs CLS.

Next, to gather additional evidence that a functional APC is required for C43-4^FITC^ to increase CLS, we added 5 μg/mL C43-4^FITC^ to the cultures of BY4741 cells harboring APC mutations (Fig. 10a to h; AUC and statistical calculations shown in Fig. 10i). C43-4^FITC^ could not increase the CLS of any of the APC mutants used and could not increase the CLS of APC mutants in W303- or BY4741-based cells, indicating that the effect is unlikely due to the genetic background. We also used a strain lacking Ama1, a meiosis-specific APC activator that is involved in spore formation (Cooper et al. 2000). We expected that *ama1Δ* cells would have a short CLS, as stationary phase often leads to meiosis under the right conditions (Reinders et al. 1998). Indeed, the deletion of AMA1 shortened CLS, which C43-4^FITC^ could not restore. This indicates that C43-4^FITC^ requires a functional APC to increase CLS in a strain background-independent manner.

**Fig. 10.**
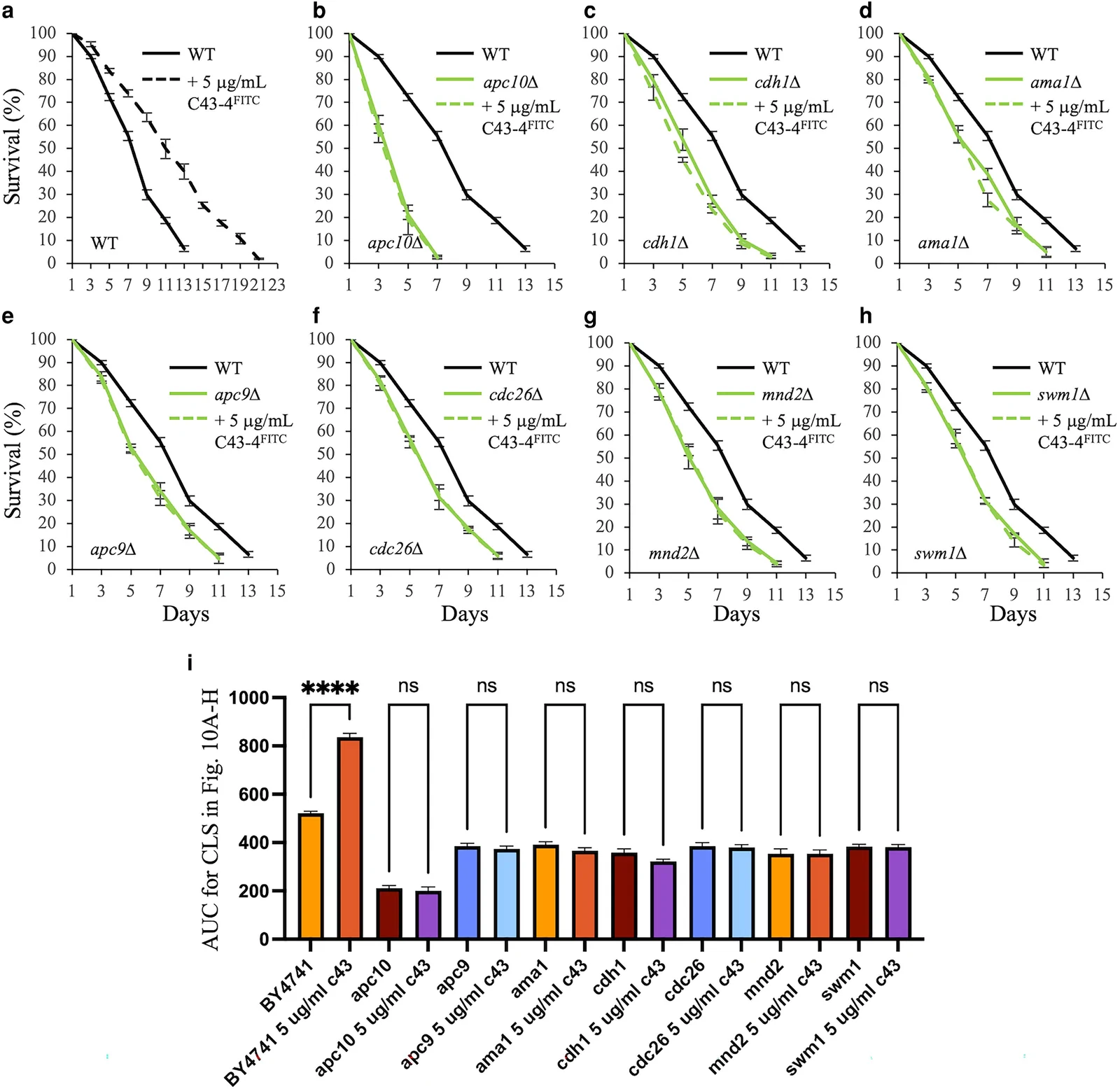
A functional APC is required for increased C43-4-dependent CLS. a) WT BY4741-based cells (YTH4505) were grown to stationary phase and allowed to chronologically age, in the presence or absence of 5 μg/mL C43-4^FITC^. Cell survival was determined and plotted using the methylene blue technique. b to h) *apc10Δ* (YTH5020), *cdh1Δ* (YTH5126), *ama1Δ* (YTH5131), *apc9Δ* (YTH5127), *cdc26Δ* (YTH5128), *mnd2Δ* (YTH5129), and *swm1Δ* (YTH5130) cells, all from the BY4741 deletion collection, were grown to stationary phase in the presence or absence of 5 μg/mL C43-4^FITC^. Cell survival was measured using the methylene blue method and compared with WT curves. The experiment was done together with the same WT with biological triplicates. In all cases, the mean ± SEM is shown. i) AUC was calculated for the CLS experiments shown above.

### C43-4 increases lifespan in *C. elegans* in a *daf-16* and *aak-2*-dependent manner

Lastly, we asked if the lifespan function of C43-4 is conserved in higher eukaryotes. We chose to test this in the worm *C. elegans.* The construct shown in Fig. 11a was cloned to express C43-4 fused to GFP under the ubiquitous *eft-3* gene promoter. Stable integration of the C43-4 transgene showed consistent expression as visualized by GFP fluorescence (Fig. 11b). Post-mitotic lifespan was then carried out on these worms. We observed that C43-4 continued to increase lifespan in worms (Fig. 11c; stats calculations shown in Supplementary Table 7; *P* < 0.001). Our work in yeast demonstrated that the APC works in a feedforward loop with the stress response factors Fkh1/Fkh2 and Snf1 (Harkness et al. 2004; Postnikoff et al. 2012; Jiao et al. 2015; Malo et al. 2016), which are members of the evolutionarily conserved FOXO3A and AMPK families, respectively. We introduced the loss-of-function mutant alleles of *daf-16* and *aak-2* into the integrated C43-4-GFP construct and performed lifespan assays. Increased lifespan induced by C43-4 partially required *daf-16* (Fig. 11d; stats calculations shown in Supplementary Table 8; *P* < 0.00001 for all combinations). Increased C43-4 lifespan also required *aak-2* [Fig. 11e; stats calculations shown in Supplementary Table 9; *P* < 0.0001 on average for all combinations, except N2 vs *aak-2* C43-4 {0.3218, 0.0033, and 1.1 × 10^−7^ in the 3 trials, and *aak-2* vs *aak-2* C43-4, which was ns in 2 out of 3 trials}]. This indicates further evolutionary conservation, as the yeast APC requires the Fkh proteins or Snf1 to sense stress and increase lifespan (Harkness et al. 2004; Postnikoff et al. 2012; Jiao et al. 2015). As a final validation, we expressed C43-4 in worms with a loss-of-function mutation in *akt-1*. These worms naturally have a longer lifespan, similar to what is observed in yeast, as *sch9Δ* mutants live long (Fabrizio et al. 2001). In *akt-1* mutant worms expressing C43-4, we observed that *akt-1* loss-of-function was epistatic to C43-4, as they continued to experience *akt-1*-dependent long life and did not show a further extension (Fig. 11f; stats calculations shown in Supplementary Table 10; *P* < 0.0001 for all comparisons, except *akt-1* vs *akt-1* C43-4, which was ns in all trials). This indicates that the induction of longer life by C43-4 may involve inhibition of the Akt-1 protein. Taken together, our data indicate that we have discovered the first APC^Cdh1^ activator that increases the lifespan of quiescent cells in yeast and worms.

**Fig. 11.**
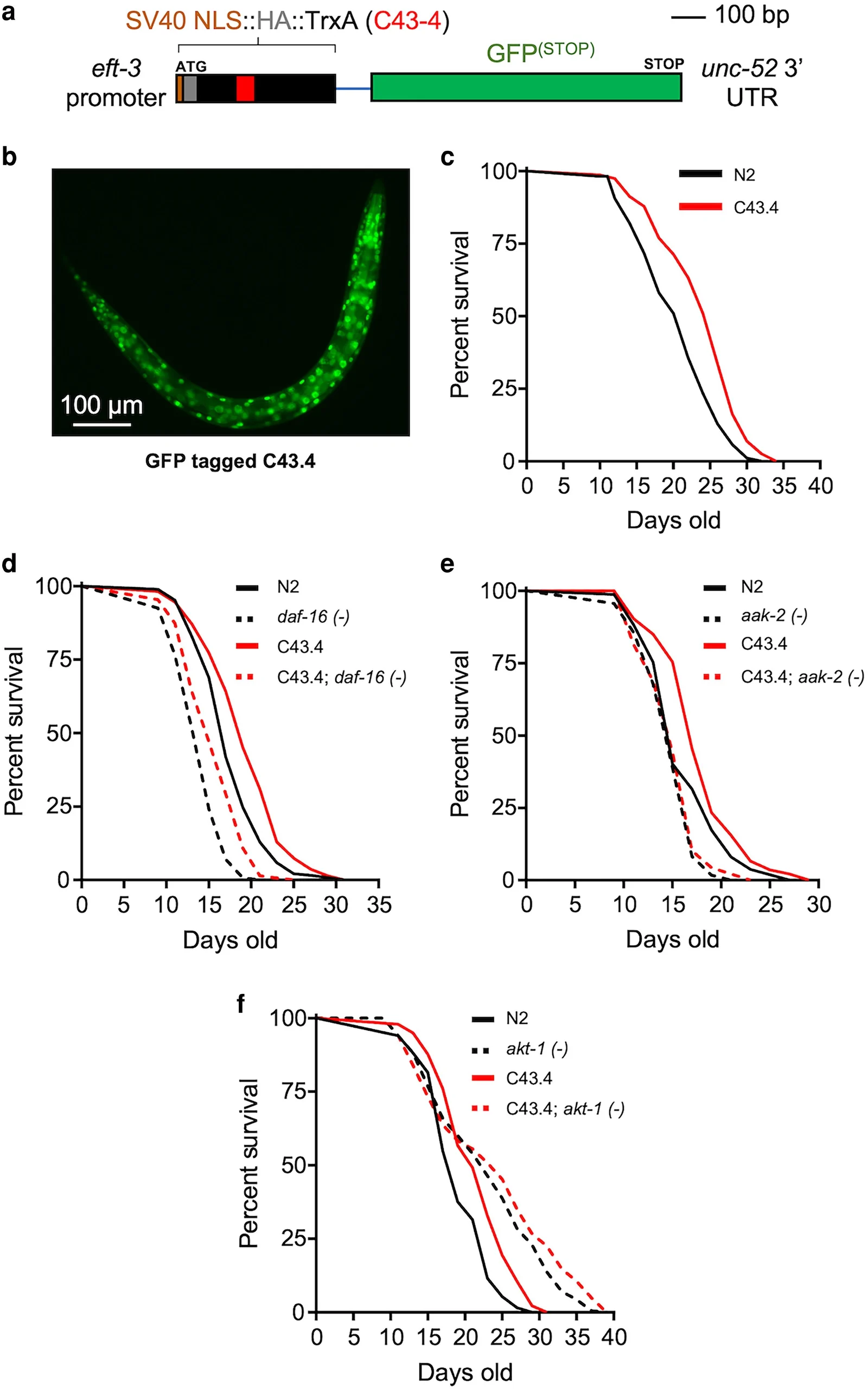
Effects of C43.4 expression in *C. elegans*. a) Schematic of the C43-4 plasmid built for *C. elegans* expression. b) C43-4-GFP expression pattern in an L4 *C. elegans*. c) Expression of C43-4 extends *C. elegans* lifespan compared with the N2 wildtype. *n* = 98 to 124 worms scored for each condition; trial 3 is shown. d) Effects of C43-4 on lifespan in the *daf-16(mu86)* loss-of-function background, *n* = 129 to 186 worms measured per condition; trial 1 is shown. The *daf-16* gene is the yeast FKH1 orthologue. e) Effects of C43-4 on lifespan in the *aak-2(ok524)* loss-of-function background, *n* = 146 to 175 worms measured per condition; trial 3 is shown. The *aak-2* gene is the yeast SNF1 ortholog. f) Effects of C43-4 on lifespan in the *akt-1(ok525)* loss-of-function background, *n* = 133 to 184 worms measured per condition; trial 1 is shown. The *akt-1* gene is the yeast SCH9 orthologue. For all lifespan assays, 3 independent trials were performed, with full results and statistics for all trials shown in Supplementary Tables 7 to 10.

## Discussion

In the study presented here, we sought to discover small peptides that directly activate APC^Cdh1^. Mammalian APC^CDH1^ activation can occur via CDK4/6 inhibitors that block the phosphorylation of CDH1, allowing APC^CDH1^ to remain active and prevent S phase transit (Mouery et al. 2024). However, these inhibitors are indirect and impact many other processes. Chemicals that indirectly activate APC^CDC20^ through SAC inhibition are available, but the one tested in this study, M2I-1, does not increase the lifespan of nondividing quiescent cells. Thus, even though SAC inhibitors are moving toward clinical trials, with unsatisfactory results (Zhou et al. 2016; Novais et al. 2021; Pinto et al. 2023), we predict that these chemicals will not increase the cell health of quiescent differentiated cells. A key example of this is the effect CDH1 has on the proper function of neurons (Almeida et al. 2005; Bobo-Jimenez et al. 2017), where CDH1 is involved in neuronal differentiation and the developing brain. A loss of APC7 was recently described to be at the heart of an intellectual disability syndrome in humans, suggesting that the APC subunit, APC7, is required for the developing mammalian brain (Ferguson et al. 2022). In these experiments, the APC was shown to target the proliferation marker, Ki-67, for ubiquitin-dependent degradation when associated with heterochromatin. Conditional knockout of CDH1 in mice, but not CDC20, led to the accumulation of Ki-67, linking APC^CDH1^, but not APC^CDC20^, with brain development. In another study, the genetic ablation of CDH1 caused the death of neural progenitor cells and microencephaly in mice (Delgado-Esteban et al. 2013). The same group showed that a missense mutation in CDH1 in a young boy was associated with severe antenatal microencephaly, psychomotor retardation, and refractory epilepsy (Rodríguez et al. 2019). Impaired CDH1 also leads to memory loss and impaired learning (Li et al. 2008; Bobo-Jimenez et al. 2017). Not surprisingly, APC^CDH1^ was linked with Alzheimer's, as it has been shown that the amyloid-β peptide causes the phosphorylation of CDH1, leading to APC^CDH1^ disassembly and loss of activity (Lapresa et al. 2022). Therefore, the need for methods to target activation of APC^CDH1^ in the aging brain cannot be understated.

In a recent study, using a yeast 2-hybrid screen, we discovered small peptides that bind to the yeast Apc10 subunit of the APC (VanGenderen et al. 2026). Apc10 is the processivity factor for the APC, required for the recruitment of substrates and the building of long K48-linked ubiquitin chains (Passmore et al. 2003; Buschhorn et al. 2011; Meyer and Rape 2011). Apc10, from yeast to humans, forms a co-receptor with Cdc20/Cdh1 to recruit substrates that contain a D-box motif, composed of the RxxL core sequence (da Fonseca et al. 2011; Qin et al. 2019). We chose to use Apc10 as our bait due to its pivotal role in recruiting and ubiquitinating substrate targets. Its broad interaction domains suggest that there is ample opportunity to manipulate Apc10 protein–protein interactions through small peptide binding.

We had shown previously that APC activity is impaired in MDR breast cancer cells (Arnason et al. 2022; Lubachowski et al. 2024). We also showed using the APC-binding peptides that MDR cancer could be reversed (VanGenderen et al. 2026). The peptides C13-3 and C43-4, stably expressed from the TrxA plasmid construct, demonstrated the best conserved activity of the peptides in human cells, showing a significant decrease in APC substrates. C13-3 and C43-4 both increased the pAPC1^S355^ activation signal and APC E3 activity, and this was associated with increased DNA damage, apoptosis, and mitotic catastrophe. Both C13-3 and C43-4 resensitized MDR breast cancer cells growing in culture to DOX, but only C43-4 stalled tumor growth in a mouse model growing MDA-MB-231 triple negative breast cancer cells stably expressing HA-TrxA-C43-4. M2I-1 also stalled tumor growth in the mouse model and acted at least additively with C43-4, indicating that M2I-1 and C43-4 activate the APC through different mechanisms.

We hypothesized in the study presented here that the APC loses function in aging cells. The rationale for this hypothesis was based on our observations in yeast that APC mutants have shorter RLS and CLS (Harkness et al. 2004) and that the APC is responsible for chromatin assembly and histone posttranslational modifications during mitosis (Harkness et al. 2002, 2005; Arnason et al. 2005; Turner et al. 2010; Islam et al. 2011). We also observed that the APC worked with the Snf1/Fkh1 stress response pathway (Harkness et al. 2004; Postnikoff et al. 2012; Menzel et al. 2014; Malo et al. 2016; Harkness 2018), all indicative that the APC would impact longevity. In this study, we validated this hypothesis by showing that the APC cannot properly target the APC substrates Clb1 or Mps1 when derived from aging cells (Fig. 1). We tested whether APC^Cdc20^ activation using the M2I-1 SAC inhibitor could increase yeast lifespan. It increased RLS, but it did not increase CLS (Fig. 2). Since M2I-1 only works on APC^CDC20^, and entrance to quiescence requires APC^CDH1^ (Marescal and Cheeseman 2020), it was not surprising that M2I-1 did not increase CLS. C43-4 increased the RLS of WT cells (Fig. 5a) and was the only peptide to increase the CLS of WT cells that we tested (Fig. 6). C43-4 required CDH1 to induce a slow growth phenotype and increase CLS (Figs. 4c to h and 10c). C43-4 was used to test whether it could maintain APC function in aging cells using the assay shown in Fig. 1. Only when C43-4 was induced with galactose in aging cells did we observe the maintenance of low levels of Clb1-TAP (Fig. 7), indicating that the APC remained active throughout the aging process.

We next synthesized the 20 amino acid C43-4 fused to an FITC motif and found that it could increase CLS by itself (Fig. 8). This indicated that C43-4 did not require the TrxA backbone it was expressed from in the plasmid, nor did it require the restrained confirmation that was offered with the TrxA backbone. Using westerns and cellular fractionation, we observed that both the HA-TrxA-C43-4 construct and the synthetic C43-4^FITC^ peptide were localized to the nucleus (Figs. 3f and 9a). HA-TrxA-C43-4 was fused to a nuclear localization signal, but C43-4^FITC^ was not. We asked if C43-4^FITC^ required the cell-penetrating TAT2 peptide to transport C43-4 into the cells, but C43-4^FITC^ increased the CLS of cells in the presence or absence of TAT2 (Fig. 8a), and C43-4^FITC^ still entered the cell and the nucleus in the absence of the cell-penetrating TAT2 peptide (Fig. 9a). Use of the scrambled peptide did not increase CLS. However, while C43-4^FITC^ was observed in the nucleus, the scrambled^FITC^ peptide did not enter the cell (Fig. 9a). Both C43-4 and scrambled^FITC^ are predicted to have a net positive charge of approximately 3 (bachem.com; biosynth.com) and to be amphipathic, which are features of cell-penetrating peptides (Svirina and Terterov 2021; Egberink et al. 2023). An enrichment of positively charged amino acids typically defines a nuclear localization signal. C43-4 and the scrambled peptide both contain 4 arginines, with 3 of them clustered in C43-4 (RVRR), but not in the scrambled peptide. The clustered stretch of arginines in C43-4 may be sufficient to allow C43-4 to cross the nuclear membrane. To test this, we created a D7A mutation in C43-4 and observed that it was capable of entering the nucleus (Fig. 9a), but did not slow the growth of cells like WT C43-4, did not increase CLS, and did not interact with Apc10 in a 2-hybrid assay (Fig. 9c to e). The C43-4^D7A^ mutant peptide provided the evidence we required to demonstrate that C43-4 increased yeast CLS and slowed growth via an interaction with Apc10. This was further supported by the observations that C43-4^FITC^ could not increase CLS in any APC mutant tested (Fig. 10), confirming that C43-4 required a fully functional APC. C43-4 could not increase the CLS of any of the 3 APC co-activator mutants tested, *cdc20-1*, *cdh1Δ,* or *ama1Δ*, indicating that C43-4 can activate all forms of the APC. Finally, both the FITC-tagged and untagged C43-4 versions increased CLS to the same extent (Fig. 9e), demonstrating that the FITC motif does not impact CLS.

The C43-4^D7A^ mutation was discovered due to sequence homology between C43-4 and Htz1, deduced from a BLAST search (Supplementary Fig. 13a). Six C43-4 amino acids were homologous to a sequence stretch observed in Htz1 (Fig. 9b), a histone variant that responds to stress (Srivatsan et al. 2018; Saatchi and Kirchmaier 2019; Brewis et al. 2025). All 6 amino acids were individually converted to alanines in C43-4. In addition to D7A creating a loss-of-function C43-4 version, the R12A and T14A mutations also reversed the slow growth phenotype of C43-4 (Fig. 9d). C43-4^R12A^ and C43-4^T14A^ both retained the ability to interact with Apc10, whereas C43-4^D7A^ did not (Fig. 9c). We synthesized the D7A mutant for our control, but future work will involve synthesizing the R12A and T14A variants. R12 was found to be conserved in all the homologous proteins identified using BLAST (Supplementary Fig. 13a) and defined the R in the RxxL motif, highlighting the importance of this motif for C43-4 function. In addition, C43-4 was found to share homology with several other proteins, 2 of which (Cst9 and Rpt1) also contained an RxxL domain. Neither Cst9 nor Rpt1 has been reported to be unstable or to interact with the APC, suggesting that these 2 proteins could be novel APC targets. We focused on Htz1 and observed genetic and functional interplay between Htz1 and the APC (Supplementary Fig. 13b to d). Strikingly, we found that the Apc10 subunit is unstable when grown in galactose but was stabilized when HTZ1 was overexpressed. Furthermore, the Htz1 protein was weakly expressed but accumulated in *apc5^CA^* and *apc10Δ* mutants, suggesting that Htz1 may be partially targeted by the APC. This suggests a novel means of controlling APC function. Reducing Apc10 levels in stressed cells could streamline APC activity to target the stress response rather than the cell cycle. Future work will investigate these ideas.

Ama1 is a third APC activator that is specific to yeast and thought to be active only in meiosis (Pesin and Orr-Weaver 2008; Cooper and Strich 2011). As discussed above, the exchangeable APC activators (one of Cdc20, Cdh1, or Ama1) and Apc10 form a 2-component substrate recognition module for substrate recruitment (Vazquez-Fernandez et al. 2024). The D-box, comprised of the base RxxL sequence, is a motif encoded by many APC substrates that is necessary for recognition by the Apc10 2-component complex (Carroll et al. 2005; Barford 2011, 2020; Morgan 2013). Strikingly, the C43-4 peptide contains an RxxL motif (Table 2). A recent study showed that peptides that mimic the D-box motif can bind to Cdc20 and inhibit APC^Cdc20^ activity (Eapen et al. 2025). We do not fully understand the mechanism at work with C43-4, but we suspect that C43-4 may mitigate Apc10-coactivator binding, possibly in the absence of substrate, prematurely activating the APC. Alternatively, C43-4 could replace Cdc20 in the co-receptor. This possibility was suggested by our observation that C43-4 could inhibit large tumor growth in mice induced by APCIN (VanGenderen et al. 2026), the APC inhibitor that works by blocking recruitment of CDC20 to the APC. Therefore, C43-4 may work in the absence of CDC20.

We have shown that C43-4 works in human breast cancer cells by resensitizing MDR cells to DOX, both in vitro and in vivo (VanGenderen et al. 2026). Here, we demonstrated that the lifespan-extending properties of C43-4 are indeed conserved. C43-4 was fused to GFP and expressed within worms (Fig. 11a and b). We found that, similar to yeast, C43-4 extended the lifespan of the *C. elegans* worm (Fig. 11c). As discussed above, the APC works within the SNF1/Fkh1 feedforward loop in yeast (Harkness et al. 2004; Postnikoff et al. 2012; Malo et al. 2016). To test if this pathway of APC control was also conserved in worms, we measured lifespan in worms expressing the AMPK mutant *aak-2* and the FOXO3A mutant *daf-16*. In both cases, the longer lifespan induced by C43-4 was lost (Fig. 11d and e), showing that in worms, APC activation by C43-4 likely works within the same AMPK/FOXO feedforward loop as in yeast. Lastly, mutation of the AKT orthologue, *akt-1*, resulted in long life that could not be extended further by C43-4 (Fig. 11f). This suggests that removal of the conserved AKT nutrient sensing kinase is important for APC-dependent lifespan increases. Consistent with this, our unpublished data indicate that the APC targets the nutrient sensor Sch9 (AKT in humans) for ubiquitin- and proteasome-dependent degradation when stress is present (unpublished).

In conclusion, we have discovered the first direct APC^Cdh1^ activator using a yeast 2-hybrid screen where Apc10 was the bait. The C43-4 peptide is the only one to require a functional APC^Cdh1^, identifying this peptide as a possible therapeutic for use in maintaining the health of differentiated, quiescent nondividing cells. Maintaining the health of quiescent cells will inhibit or delay the transition of these cells to senescent cells, which occurs upon the accumulation of damaged DNA and proteins as cells age. Senescence is considered a key hallmark of aging, suggesting that the inhibition or delay of senescence, such as that provided by the C43-4 peptide, could delay aging. Protecting the health of differentiated quiescent cells could also be vital to protect against the development of cancer in differentiated cells and against neurodegeneration in neurons and the brain. Characterizing how C43-4 interacts with the APC, and possibly directly with Apc10, will provide the blueprint for developing further therapeutics that can be used to delay aging and the onset of age-related disease.

## Supplementary Material

iyag249_Supplementary_Data

iyag249_Peer_Review_History

## Data Availability

No new databases were generated in this study. Strains and plasmids are available upon request. The authors affirm that all data necessary for confirming the conclusions of the article are present within the article, figures, and tables. Supplemental material available at GENETICS online.
