# Supplementary material for: Discovery of a small peptide that increases yeast lifespan by enhancing the function of APCCdh1 in nondividing quiescent cells
Source: Genetics. 2026 Sep 16;234(3):iyag249. doi: 10.1093/genetics/iyag249 (PMC13732707; doi:10.1093/genetics/iyag249)
Supplement: Supplementary file 1 [file iyag249_peer_review_history.pdf]

GENETICS

Discovery of a small peptide that increases yeast lifespan by enhancing the function of APCCdh1 in nondividing quiescent cells

Rachel Harris, Spike Postnikoff, Nishita Shukla, Alexandra Harkness, Hannah Brooks, Abd-Al-Wahab Khawaja, Mackenzie Malo, Carlos Zapien-Verdugo, Brandon Waddell, Cheng-Wei Wu, and Troy Harkness

NOTE: The reviews and decision letters are unedited and appear as submitted by the reviewers.
In extremely rare instances and as determined by a Senior Editor or the EIC, portions of a review may be redacted. If a review is signed, the reviewer has agreed to no longer remain anonymous.
The review history appears in chronological order.

Review Timeline:

Submission Date:	2026-04-06
Editorial Decision:	2026-05-07
Resubmission Received:	2026-08-05
Accepted:	2026-08-30

1st Editorial Decision: May 7, 2026

May 7, 2026

GENETICS-2026-309337
Discovery of a small peptide that increases yeast lifespan by enhancing the function of APCCdh1 in nondividing quiescent cells

Dear Dr. Harkness:

Two experts in the field have reviewed your manuscript, and I have read it as well. We all think that your findings are interesting and will likely be impactful for the field. While your manuscript is not currently acceptable for publication in GENETICS, we would welcome a substantially revised manuscript. Both reviewers have comments and concerns to be addressed in a revised manuscript. You can read their reviews at the end of this email.

You can see that most of the reviewers comments suggest improvements to readability and clarifications of the text. Please consider the suggested changes and also consider the experimental addition. Importantly, make sure the methods includes all experiments in the manuscript. We look forward to receiving your revised manuscript. Please let the editorial office know approximately how long you expect to need for revisions.

Upon resubmission, please include:
1. A clean version of your manuscript;
2. A marked version of your manuscript in which you highlight significant revisions carried out in response to the major points raised by the editor/reviewers (track changes is acceptable if preferred);
3. A detailed response to the editor's/reviewers' feedback and to the concerns listed above. Please reference line numbers in this response to aid the editor and reviewers.

Your paper will likely be sent back out for review.

Additionally, please ensure that your resubmission is formatted for GENETICS
https://academic.oup.com/genetics/pages/general-instructions

Follow this link to submit the revised manuscript: Link Not Available

Sincerely,

Soni Lacefield
Associate Editor
GENETICS

Approved by:
Meera Sundaram
Senior Editor
GENETICS

Reviewer #1 :

This work utilizes a peptide that this group seems to have identified in another study (in press) to activate the mammalian Anaphase Promoting Complex (APC), and they use it here to show that this APC activating peptide extends the yeast chronological lifespan and replicative lifespan, and the worm lifespan. This is an important finding with translational potential. The work will be of interest to the readership of Genetics and beyond. The clarity of the manuscript could be improved and made tighter with some simple changes (listed largely in order of appearance in the manuscript.)

1. The Abstract is very long, with half of it being background information and lacks clarity, some examples are given below - a tighter abstract would be more palatable. The abstract would also benefit from being specific when talking about lifespan (i.e. is it chronological or replicative). Examples: "Using yeast, we have observed that the Anaphase Promoting Complex (APC) protects nondividing quiescent cells from entering senescence by extending the lifespan of these cells." Please clarify - is this chronological or replicative lifespan?

"We propose that activation of the APCCdh1 subcomplex in yeast will maintain the health of quiescent cells, extending their lifespan." Please clarify - is this chronological or replicative lifespan?

"Loss of Cdh1 blocks the ability of cells to enter quiescence, resulting in genomic instability and reduced chronological lifespan of stationary phase (G0/quiescent) cells." Do cells that have a blocked ability to enter quiescence have a longer replicative lifespan?
The abstract and results sections discuss the apc5CA mutant without any description of what it is / does. What is a "translational peptide"?

2. The beginning of the results section states: "We previously demonstrated that the APC loses function in drug-resistant MDA-MB-231 breast cancer cells, and that activating either APCCDC20 or APCCDH1, using M2I-1 or C43-4, respectively, rendered these cells sensitive to drug therapy," Page 7 - please specify what kind of drug. Also, could the authors better explain the logic of how this observation led them to hypothesize that "APC loses function in aging quiescent yeast cells" as surely the drug-resistant MDA-MB-231 breast cancer cells that had lost APC function are still dividing... how would this then predict loss of APC function in quiescent (non-dividing) yeast cells? This can simply be fixed by more explicit statements.

3. Please increase the size of some of the text (the very small gene names) in Fig. 1A. Also, the red crosses are too thick to read the gene names underneath, please make the lines thinner on the crosses. The naming of the different phases on the diagram twice is not necessary for this journal, G1, S, G2 and M would be sufficient.

4. In the legend to Fig. 1B "APC (left) or SCF (right)" should be APC (top) or SCF (bottom)" perhaps?

5. Fig. 1D-F. It is not clear to this reviewer why it was necessary to bring the cells out of quiescence back into cycling to measure levels of APC substrates Clb1, Mps1 and Sic1 during chronological aging if they wanted to know how APC activity was during quiescence? i.e. Why weren't the westerns just performed on the quiescent cells (G1)? i.e. the state of the APC substrates in cells that are brought out of quiescence does not necessarily reflect the state of APC substrates during quiescence as they are now dividing and APC is being regulated in a different manner in cycling cells. Also, labelling of the quantitation of the westerns is too small, and should be made the same size as the other text in the westerns.

6. Fig. 2E: The key shows 9 conditions, but only the CLS data for three conditions are shown in the figure.

7. In discussing Fig. 2C "We found that treatment of WT cells with M2I-1 increased the
RLS by roughly 50% at the highest dose used "this should be qualified as "mean /median RLS", as it is not increasing the max RLS.

8. What was the "library of random small peptides" used in the two-hybrid analysis (no method section exists)? Also, the two-hybrid data is not shown, just a table of 8 peptide sequences. What were the relative strengths / scores of the interactions? One of these peptides is only 3 amino acids... Does it make sense that activating the APC (something that promotes progression through the cell cycle), would lead to slow growth? None of the interactions identified by two-hybrid are confirmed in IPs - not essential but that would have been nice, perhaps in the next paper when they map the site of interaction etc (see below).
As I read the discussion, it cites the two-hybrid screen to find these peptides being performed in another publication of theirs in press. If that is the case, this section of the results on the identification of peptides by two hybrid needs to be deleted, along with Table 1.

9. Are the reduced levels of APC substrates upon induction of the C43-4 peptide as shown by western in Fig. 3F dependent on the APC? i.e. is this effect still seen in a mutant lacking the critical subunit that this peptide is meant to bind to? This would prove the specificity of the result, rather than it being due to indirect effects. This is not a necessity but would be a nice control if the authors have the data handy.

9. There are several issues with Figure 4A, the wild type strain background was very short lived - why? - and 2% glucose would have completely repressed the GAL1 promoter expressing the peptides (due to Gal80 blocking Gal4 binding plus via the dephosphorylated Mig1 repressor), so why did the C2-4B and CI3-3 extend the lifespan? They talk about glucose leading to low peptide expression (which goes against all literature on glucose repressing this promoter). Was expression of the peptide monitored and reduced levels of APC substrates shown upon glucose addition? Same thing with the lifespan extensions seen upon glucose addition in Fig. 4B and 4F. These results are quite concerning as glucose should not be inducing this promoter, unless they used a different promoter from the one described (Gal1).

10. Fig. 4C-E show that expression of peptide C43-4 with 1% and 2% galactose don't extend the RLS significantly yet 0.5% galactose does. If they want to include this data, they should show by western blots that 1 and 2% galactose make a lot more of the peptide and / or result in lower levels of APC substrates, to make this dataset complete and could then be interpreted as having a dose response effect. Otherwise, just show the data in Fig. 4E and remove the other panels.

11. Again, regarding Fig. 4F, without knowing what the apc5CA mutant is, it makes it difficult to understand how an APC activator peptide (C43-4) can increase the chronological lifespan of an apc mutant - does this apc5CA mutant take out the function of the APC? Also, this experiment again is done in glucose that should be repressing the expression of the peptide. If they are going to keep this data, they need to show that the peptides that increase the RLS of the apc5CA mutant also increase

the activity of the APC (by looking at substrate protein levels).

12. The CLS curves for the wild type within Fig. 5 are inconsistent, despite identical media being used. Panels A-H showing a median lifespan of around 4 and max of 9 days, while in Fig. 5I the WT has a median lifespan of around 11 days and max of 21 days. This variability is concerning. Fig. 5A-D (showing a bunch of other peptides that won't be discussed again in the paper fail to extend the CLS) should be probably put in the supplemental section or deleted, especially because two of the curves (the EV curves) in each panel appear to be from the identical data in all four panels.

13. Data showing that the membrane soluble peptide used in Fig. 7 increases APC activity, or any other established phenotype of APC activity would be beneficial.

14. Does the C43-4 peptide resemble the sequence of any known activator / interactor of APC? If you do an alpha fold 3 prediction of the peptide with APC10, where does the peptide interact? Future studies may want to mutate the predicted interaction site on APC10 / the peptide to confirm the prediction to learn the mechanistic basis of APC activation by this peptide. Additionally, we don't learn how the APC activity goes down during quiescence - is it because there is less of the APC proteins, or less of the regulators? Accordingly, does the 43-3 peptide increase APC subunit levels in quiescent cells?

15. Figure 7, characterization of the peptides to show that their phenotypes are specific to the APC, should be inserted after Figure 3, as its current location in the paper disrupts the flow of the lifespan analyses in the second half of the paper. Also, Figure 8 (showing the 43-3 peptide activates the APC during the CLS) should be inserted after Fig. 5 (showing that the 43-3 peptide extends the CLS).

16. Just a conceptual question for the authors to address in the discussion - the APC drives progression through M phase to G1 phase. As such, how do the authors imagine that the APC plays a role in quiescent / non dividing (G1/G0 phase) cells? Is the fact that the CLS assay requires a somewhat artifactual ability of the cells to form colonies again, i.e. grow again after leaving quiescence, be where the APC activation is playing a role, rather than during quiescence? If so, how does APC function relate to quiescence in the aging brain (after brain development and cell division is finished), where the cells will not be dividing again, regarding the statement "Therefore, the need for methods to target activation of APCCDH1 in the aging brain cannot be understated."

17. There is method section on measuring half-life with cycloheximide, but no results presented used this approach. Please remove.

Reviewer #3 :

Harris et al. show that APC function declines as cells age and hypothesize that activating the APC/Cdh1 complex could block the transition from quiescence to senescence. This would extend chronological lifespan and represent a strategy for healthspan extension.By screening a set of previously identified Apc5- and Apc10-interacting peptides for APC activation, they identify peptide C43-3. Controlled expression of this peptide reduces levels of APC substrates Clb1 and Mps1 and increases chronological lifespan of yeast in an APC-dependent manner. Importantly, the effect is observed in both young and old yeast cells, implying that both a preventive and restorative effect on cellular fitness can be mediated by controlled (re)activation of APC-Cdh1. A synthetic C43-4 peptide also extends CLS, and a similar anti-aging effect is observed in worms expressing the peptide. The study is a thorough, large body of work, and the experiments are well executed. The identification of a small peptide activator of APC is a major advance. I have the following comments.

1. The RxxL motif in C43-3 is noteworthy for reasons pointed out by the authors. Mutagenesis of this motif in one experiment would strengthen the proposed model of how this peptide impacts APC and allow comments on the use of this peptide as a scaffold for future optimization. A synthetic mutant peptide or a mutant form of the yeast construct seems reasonably straightforward.
2. Considerable efforts to control experiments with a scrambled C43-3 (+TAT) peptide were made, which is commendable, but this peptide still fails to enter the nucleus. A C43-3-like peptide that enters the nucleus but does not interact with APC-Cdh1 would be a preferred control. In regard to this, a) what is Peptide 2 in Supp. Fig. 10B, and does it meet this criterion? b) Has a scrambled peptide been expressed in the TrxA vector? Addressing point 1 above would also address this issue, but with added value.
3. How stable is the C43-4 peptide when expressed in yeast, and when applied exogenously to cultures? From Fig. 3f, the Trx fusion has a half-life in the range of hours, but the peptide addition experiments in Fig. 6C imply the peptide was added as the cultures grew to saturation. Does this mean the effective peptide concentration in the aging interval of days may in fact be much lower?
4. Is there a maximal benefit of APC activation that is capped by APC protein levels? Has a time course of APC subunit protein

levels across chronological age been performed?
5. The legend of Fig. 6 is out of order with the data panels.
6. Please clarify which experiments involved the CHX pulse-chase described in the Methods.

Associate Editor Comments:

Aug. 4, 2026

We thank the reviewers and the editor for the time committed to reading the manuscript and for providing excellent input on how to improve the draft. Your insight was perfect for the changes that needed to be made. I had to make some alterations listed below. Comments specific for the reviewer suggestions follow where each point was made.

Thank you again for your help improving the manuscript.

Troy

Changes made to the figures and manuscript.

1. Figs. 1D-F, changed the Y-axis of the ratio graph so it is normalized to 1.

2. Fig. 1E, replaced the mitosis graph to better reflect the western. Changed the molecular weight markers on the western to correctly reflect the molecular weight of 105 kDa (was 75 kDa). Replaced the ratio graph to reflect the correct mitosis graph. Changed the Y-axis of the ratio graph so that it is normalized to 1 in log phase (was reflected as fold change normalized to 100%).

3. Fig. 1F, changed the molecular weight of Sic1-TAP from 75 to 52 kDa. Regraphed the ratio to better reflect the westerns. Changed the Y-axis of the ratio graph so is it now normalized to 1 at log phase.

4. Removed peptide C6-5 from the Figs. 3 and 5. We resequenced the peptides and C6-5 could not be validated. This peptide was removed from the paper. This did not impact the results in any way.

5. Added uncropped blots for Figures 3E-G to the reordered Supplemental Figure 6. Incorporated a Gluc/Gal time course induction of C43-4 with Clb1-TAP reduction to match the Suc/Gal experiment in Figure 3G. This experiment shows that the Gal promoter is shut down slower with Suc compared to Gluc, resulting in a quicker expression of Clb1-TAP following Sucrose promoter shut off.

6. Changes in the text were made to correctly reflect the changes to the figures.

7. New Figures were added to the main manuscript (Figure 9) and to the Supplementary Figures (Supplemental Figure 13) to address a reviewer's comment about comparing C43-4 sequence with other proteins. This allowed us to mutate C43-4 and discover an amino acid replacement that disrupts C43-4-Apc10 2-hybrid interactions and abolishes growth and CLS phenotypes observed with C43-4. We feel this adds to the paper and allowed us to generate a peptide control that enters the yeast nucleus but does not activate the APC. I have added three authors to the paper who did this work: Hannah Brooks, Abdal Khawaja and Mackenzie Malo.

8. The methods section was moved to follow the Introduction.

9. TrxA-HA-C43-4 was rewritten throughout the text to HA-TrxA-C43-4 to reflect the correct order of the motifs.

Reviewer #1 :

This work utilizes a peptide that this group seems to have identified in another study (in press) to activate the mammalian Anaphase Promoting Complex (APC), and they use it here to show that this APC activating peptide extends the yeast chronological lifespan and replicative lifespan, and the worm lifespan. This is an important finding with translational potential. The work will be of interest to the readership of Genetics and beyond. The clarity of the manuscript could be improved and made tighter with some simple changes (listed largely in order of appearance in the manuscript.)

1. The Abstract is very long, with half of it being background information and lacks clarity, some examples are given below - a tighter abstract would be more palatable. The abstract would also benefit from being specific when talking about lifespan (i.e. is it chronological or replicative). Examples: "Using yeast, we have observed that the Anaphase Promoting Complex (APC) protects nondividing quiescent cells from entering senescence by extending the lifespan of these cells." Please clarify - is this chronological or replicative lifespan?

"We propose that activation of the APCCdh1 subcomplex in yeast will maintain the health of quiescent cells, extending their lifespan." Please clarify - is this chronological or replicative lifespan?

"Loss of Cdh1 blocks the ability of cells to enter quiescence, resulting in genomic instability and reduced chronological lifespan of stationary phase (G0/quiescent) cells." Do cells that have a blocked ability to enter quiescence have a longer replicative lifespan? The abstract and results sections discuss the apc5CA mutant without any description of what it is / does. What is a "translational peptide"?

The abstract has been rewritten.

The *apc5*^{CA} mutant was first described in Harkness et al. 2002. In the text, line 414, I have added, "The *apc5*^{CA} mutant was in the essential *APC5* gene that was isolated in a screen for chromatin assembly (CA) mutants (Harkness et al. 2002). The *apc5*^{CA} allele contains a deletion of nucleotides 36 and 37, generating a novel amino acid sequence after amino acid 12, with a stop codon 18 amino acids later, producing a temperature-sensitive phenotype at 37°C. When the *apc5*^{CA} allele was TAP tagged on the C-terminus, it resulted in a shorter, weakly expressed protein (Menzel, J. MSc thesis, 2014), indicating the *apc5*^{CA} phenotype was likely due to translational slippage."

2. The beginning of the results section states: "We previously demonstrated that the APC loses function in drug-resistant MDA-MB-231 breast cancer cells, and that activating either APCCDC20 or APCCDH1, using M2I-1 or C43-4, respectively, rendered these cells sensitive

to drug therapy," Page 7 - please specify what kind of drug. Also, could the authors better explain the logic of how this observation led them to hypothesize that "APC loses function in aging quiescent yeast cells" as surely the drug-resistant MDA-MB-231 breast cancer cells that had lost APC function are still dividing... how would this then predict loss of APC function in quiescent (non-dividing) yeast cells? This can simply be fixed by more explicit statements.

This comment has been removed from the beginning of the Results and moved to the Discussion, where it will be discussed further.

3. Please increase the size of some of the text (the very small gene names) in Fig. 1A. Also, the red crosses are too thick to read the gene names underneath, please make the lines thinner on the crosses. The naming of the different phases on the diagram twice is not necessary for this journal, G1, S, G2 and M would be sufficient.

This has been corrected. The protein names have been increased. The extra cell cycle names have been removed and the red crosses have been removed. Instead of the red crosses I have shown that the proteasome degrades the proteins tagged with Ub with degraded protein names.

4. In the legend to Fig. 1B "APC (left) or SCF (right)" should be APC (top) or SCF (bottom)" perhaps?

This has been corrected on line 381.

5. Fig. 1D-F. It is not clear to this reviewer why it was necessary to bring the cells out of quiescence back into cycling to measure levels of APC substrates Clb1, Mps1 and Sic1 during chronological aging if they wanted to know how APC activity was during quiescence? i.e. Why weren't the westerns just performed on the quiescent cells (G1)? i.e. the state of the APC substrates in cells that are brought out of quiescence does not necessarily reflect the state of APC substrates during quiescence as they are now dividing and APC is being regulated in a different manner in cycling cells. Also, labelling of the quantitation of the westerns is too small, and should be made the same size as the other text in the westerns.

We reintroduced the cells back into the cell cycle following quiescence in order to observe the degradation of the substrates. Clb1 and Msp1 are not present (or at least at low levels) and Sic1 is not degraded in quiescent cells. Thus, we cannot measure APC and SCF substrate degradation by simply doing westerns on lysates prepared from chronologically aging quiescent cells. By arresting cells when substrates are highest and lowest after they re-enter the cell cycle out of quiescence enables us to observe the degradation of substrates with the APC that is present in aging quiescent cells. We propose that the modifications that control cell cycle APC function rely on epigenetic changes to APC subunits by pathways that may erode over time. Therefore, we are asking how well an APC from aging cells targets its substrates in the subsequent cell cycle. This is clarified in the text (see lines 356-362). Labeling in the quantitation Figures 1D-F has been enlarged, as

have the labels in Fig. 1C. This has also been done in Figure 8.

6. Fig. 2E: The key shows 9 conditions, but only the CLS data for three conditions are shown in the figure.

Figure 2E actually contains 9 curves, WT, *apc5^{CA}* and *apc10Δ* treated with M2I-1, APCIN, or left untreated. M2I-1 and APCIN have no impact on CLS, so the treatments are the same as the untreated samples and overlap. This text was added to the Fig. 2E legend on line 454: The treatments had no effect on CLS, therefore the overlap of the 3 CLS experiments for each strain looked like a single CLS experiment.

7. In discussing Fig. 2C "We found that treatment of WT cells with M2I-1 increased the RLS by roughly 50% at the highest dose used "this should be qualified as "mean /median RLS", as it is not increasing the max RLS.

This has been corrected on line 461.

8. What was the "library of random small peptides" used in the two-hybrid analysis (no method section exists)? Also, the two-hybrid data is not shown, just a table of 8 peptide sequences. What were the relative strengths / scores of the interactions? One of these peptides is only 3 amino acids... Does it make sense that activating the APC (something that promotes progression through the cell cycle), would lead to slow growth? None of the interactions identified by two-hybrid are confirmed in IPs - not essential but that would have been nice, perhaps in the next paper when they map the site of interaction etc (see below). As I read the discussion, it cites the two-hybrid screen to find these peptides being performed in another publication of theirs in press. If that is the case, this section of the results on the identification of peptides by two hybrid needs to be deleted, along with Table 1.

I originally had the 2-hybrid screen in this manuscript, but it had to be moved from this paper to the paper studying the effects of the peptides on cancer (VanGenderen et al., Sci Rep, in press), due to the timing of the papers. The library was obtained from a colleague as described in VanGenderen et al. 2026. That paper describes the screen, so reference to the 2-hybrid will be kept to a minimum. We have renamed the section from "**Identification of peptides that bind to Apc5 or Apc10**", to "**Characterization of peptides that bind to Apc10**" on line 473. We have several projects going now to do exactly as the reviewer suggests. We have been working on CoIPs using several approaches. We have fused the maltose binding protein to C43-4 and are using MBP-TRAP columns to identify C43-4 binding proteins. Since C43-4 was identified as binding to Apc10, the CoIP and column experiments are using strains that lack *APC10*, in addition to the wild type cells. At present, these experiments are not complete and would be beyond the scope of this paper. But this is something we have been putting a lot of effort into for the past several years.

We have shown that the APC interacts with the stress response pathway through genetic and physical interactions with the SNF1 kinase and Fkh1/Fkh2 pathways. We have shown

that SNF1/Fkh/APC are involved in a feedforward pathway that responds to stress (Harkness et al. 2004; Postnikoff et al. 2012; Jiao et al. 2015; Malo et al 2016). When the stress pathway is active, it shifts resource use from growth and division to repair and maintenance. Under these conditions cell cycle progression is slowed. We did not follow up on this mechanism in this paper as this paper sought to describe the effects of the peptide on lifespan. The slowed growth we observe in our experiments with C43-4 is consistent with C43-4 activating the APC, with a subsequent increase in repair pathways. Current and future work are addressing these concepts.

9. Are the reduced levels of APC substrates upon induction of the C43-4 peptide as shown by western in Fig. 3F dependent on the APC? i.e. is this effect still seen in a mutant lacking the critical subunit that this peptide is meant to bind to? This would prove the specificity of the result, rather than it being due to indirect effects. This is not a necessity but would be a nice control if the authors have the data handy.

The old Figure 3F is the new Figure 3G. This is a good point. We did not do this exact experiment. However, we do show in this and the previous draft that increased CLS by C43-4 depends on the APC, as all APC mutants tested could not support extended CLS by C43-4 (see Figures 6H, 8D, and 10).

9. There are several issues with Figure 4A, the wild type strain background was very short lived - why? - and 2% glucose would have completely repressed the GAL1 promoter expressing the peptides (due to Gal80 blocking Gal4 binding plus via the dephosphorylated Mig1 repressor), so why did the C2-4B and CI3-3 extend the lifespan? They talk about glucose leading to low peptide expression (which goes against all literature on glucose repressing this promoter). Was expression of the peptide monitored and reduced levels of APC substrates shown upon glucose addition? Same thing with the lifespan extensions seen upon glucose addition in Fig. 4B and 4F. These results are quite concerning as glucose should not be inducing this promoter, unless they used a different promoter from the one described (Gal1).

There are several issues with Figure 4A, the wild type strain background was very short lived - why? The old Figure 4 is now new Figure 5. The wild type RLS in this Fig. 5A is short because of the media we used. We maintained the cells on trp- media in order to maintain the plasmid. Wild type cells have a very short RLS on trp- media. In future experiments we simply used YP media without selection, as we observed that plasmid loss was not noticeable in the RLS expts. We included this figure as 2 of the peptides increased the RLS under these conditions.

- and 2% glucose would have completely repressed the GAL1 promoter expressing the peptides (due to Gal80 blocking Gal4 binding plus via the dephosphorylated Mig1 repressor), so why did the C2-4B and CI3-3 extend the lifespan? They talk about glucose leading to low peptide expression (which goes against all literature on glucose repressing this promoter). In a previous publication (Turner et al. 2010, Euk Cell) we addressed the

concern with studying *GAL* promoter driven expression when using glucose supplemented media. We used qPCR in cells expressing a galactose inducible construct in cells grown overnight in glucose, then induced with 2% galactose for 3 hours. qPCR showed that the construct was induced over 100-fold in glucose and over 900-fold with galactose (Figure 5B, Turner et al.). A western against a galactose driven HA-tagged gene grown in glucose or galactose showed that the HA signal was visible in glucose but heavily overexpressed following 2 hours of gal induction (Fig. 5C, Turner et al.). In Fig. 5F of the that paper we showed that the *apc5^{CA}* (the *apc5^{CA}* mutant will be described below, and in the text) temperature defect can be suppressed when *GAL*-driven constructs expressing *APC5*, *APC10* and *RTT109* are expressed on glucose. These studies show that GAL-induced constructs are still expressed when grown on glucose, just at a much lower level. Since we still see effects from our galactose inducible constructs on glucose, we conclude that low level expression of the peptides is more effective than high level expression. We have observed this previously with *APC5* and *FKH1* over expression. Galactose induced over expression of *APC5* was toxic to cells (Tuner et al. 2010, Fig. 5E) and copper induction of *APC5* shortened RLS (Harkness et al. 2004, Fig. 3). Induction of galactose driven *FKH1* or *FKH2* using 2% galactose was toxic to cells (Postnikoff et al. 2012, PLoS Genetics, Figs. 3A, and 5B). On the other hand, induction of *FKH1* or *FKH2* using 0.05% galactose increased CLS and RLS of the cells (Postnikoff et al. 2012, PLoS Genetics, Fig. 4). Interestingly, when the *FKH1* and *FKH2* galactose driven constructs were grown on glucose, they suppressed the *apc5^{CA} ts* defect and promoted better growth of wild type cells (Postnikoff et al. 2012, Fig. 5B). In our Menzel et al. 2014 paper, we showed that a stabilized version of Fob1-HA expressed under the galactose inducible promoter was easily observed when grown in 2% glucose media. These are examples of how growth of galactose driven constructs on glucose can be used when low level expression is required. We added a short statement relating to this on line 622: …", as we have shown previously that 2% glucose results in low, but detectable levels of expression of galactose inducible promoters (Turner et al. 2010; Postnikoff et al. 2012; Menzel et al. 2014)."

The toxic effects of *FKH1*, *FKH2* and *APC5* when expressed at high levels is consistent with the idea that increasing APC activity in aggressive cancer cells causes cells with high loads of damage to enter mitosis faster than usual, bypassing the need for DNA repair, causing mitotic catastrophe (reviewed in VanGenderen et al. 2020). We showed using the APC activator Mad2 Inhibitor 1 (M2I-1; used in the study here) in drug resistant cancer cells that APC activation pushed cells more rapidly into mitosis and this was linked to increased cell killing (Lubachowski et al. 2024). Therefore, we believe that high level activation of the APC/FKH feedforward loop causes cells to cycle through mitosis faster than normal, allowing for increased mutation and DNA damage due to less time for repair, causing toxicity. In the Postnikoff et al. 2012 paper, we showed that overexpression of FKH1/2 caused apoptosis (Postnikoff PhD thesis). On the other hand, lower activation of the feedforward loop appears to be a benefit to cells, possibly by increasing cell repair mechanisms. Therefore, we recognize that dosing of APC activators will be important for therapeutic purposes in the future.

Was expression of the peptide monitored and reduced levels of APC substrates shown upon glucose addition? We showed in the new Figure 3G of this paper that C43-4-HA peptide levels increased over time with 1% or 2% gal induction with equal expression, and that this coincided with reduced levels of Clb1-TAP, an APC substrate. In the same expt., we used glucose or sucrose to turn off the *GAL* promoter following galactose induction, resulting in reduced expression of C43-4-HA and the restoration of Clb1-TAP levels, as explained above. So, we did observe reduced substrate levels following promoter activation, and increased levels following peptide promoter shutoff under 2 different conditions. We performed a time course experiment rather that a dose response.

10. Fig. 4C-E show that expression of peptide C43-4 with 1% and 2% galactose don't extend the RLS significantly yet 0.5% galactose does. If they want to include this data, they should show by western blots that 1 and 2% galactose make a lot more of the peptide and / or result in lower levels of APC substrates, to make this dataset complete and could then be interpreted as having a dose response effect. Otherwise, just show the data in Fig. 4E and remove the other panels.

We have removed Figures 4C and 4D. The results in Figures 4C and 4D are consistent with a lower dose being more effective than a higher dose, but we are yet to obtain a western showing this. Therefore, we have removed those figures and changed the text and figure legend to reflect this change.

11. Again, regarding Fig. 4F, without knowing what the apc5CA mutant is, it makes it difficult to understand how an APC activator peptide (C43-4) can increase the chronological lifespan of an apc mutant - does this apc5CA mutant take out the function of the APC? Also, this experiment again is done in glucose that should be repressing the expression of the peptide. If they are going to keep this data, they need to show that the peptides that increase the RLS of the apc5CA mutant also increase the activity of the APC (by looking at substrate protein levels).

Figure 4F has been removed. While I think the figure is informative and important, it needs more work and I do not have an explanation for why C43-4 requires the APC for CLS, but not RLS. Future studies will address this issue.

12. The CLS curves for the wild type within Fig. 5 are inconsistent, despite identical media being used. Panels A-H showing a median lifespan of around 4 and max of 9 days, while in Fig. 5I the WT has a median lifespan of around 11 days and max of 21 days. This variability is concerning. Fig. 5A-D (showing a bunch of other peptides that won't be discussed again in the paper fail to extend the CLS) should be probably put in the supplemental section or deleted, especially because two of the curves (the EV curves) in each panel appear to be from the identical data in all four panels.

We removed panel 5B from this figure which showed C6-5, since this peptide was removed from the paper and had no impact on the results. The final panel in Figure 5 (new Figure H)

shows a longer WT mean and max CLS than that in panels 5A-G. This is because panels A-G used depleted minimal media (MM)-Trp, whereas the CLS experiments in panel H used depleted SD-Trp media. MM has a 10-fold reduction in amino acids compared to SD (see methods). It is our fault for not making this clearer in the legend and text. This has been corrected.

13. Data showing that the membrane soluble peptide used in Fig. 7 increases APC activity, or any other established phenotype of APC activity would be beneficial.

The figure is now Figure 8. We have not yet generated data using the membrane permeable peptide that require plates due to the cost of adding the peptide to plates. In other work, we have shown that the synthetic peptide causes reduced levels of Sch9-HA, which is due to APC activity, and is not supported by the D7A mutant peptide. This will be described elsewhere.

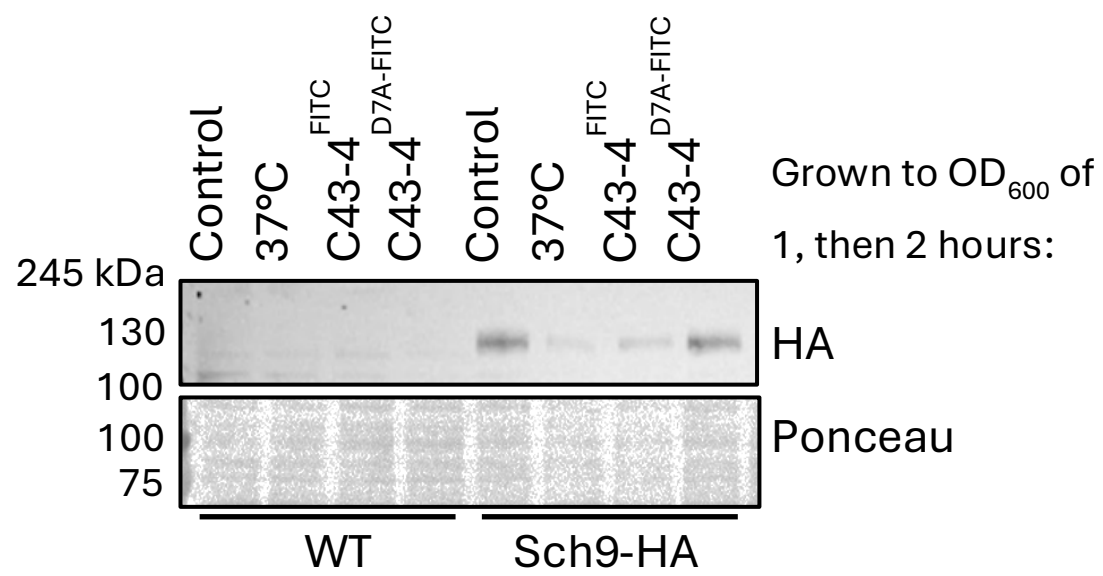

14. Does the C43-4 peptide resemble the sequence of any known activator / interactor of APC? If you do an alpha fold 3 prediction of the peptide with APC10, where does the peptide interact? Future studies may want to mutate the predicted interaction site on APC10 / the peptide to confirm the prediction to learn the mechanistic basis of APC activation by this peptide. Additionally, we don't learn how the APC activity goes down during quiescence - is it because there is less of the APC proteins, or less of the regulators? Accordingly, does the 43-3 peptide increase APC subunit levels in quiescent cells?

These are excellent suggestions. We are currently working on these experiments. We have used BLAST to determine if any yeast proteins have similar sequence motifs to C43-4 (new **Supplemental Figure 13A**). We have included these figures in the revised manuscript. Our control peptide, C43-4^{D7A}, that we are using was derived from mutagenesis of amino acids within C43-4 that share homology with Htz1 (**Figure 9B; Supplemental Figure 13A**). We showed that *HTZ1* genetically interacted with the APC, accumulated at the protein level in APC mutants, and stabilized Apc10 (**Supplemental Figure 13**). The mutant peptides were then used in a 2-hybrid assay to determine if any of the mutants disrupt the Apc10-C43-4 2-hybrid interaction. D7A was the only mutant to disrupt the interaction (**Figure 9C**). The mutants were then expressed in WT cells and spot diluted onto glu and gal plates. (**Figure 9D**). Three of the mutants, D7A, R12A and V14A, could not slow the growth of cells similar to the WT peptide. D7A disrupts the 2-hybrid interaction while R12 and V14 are part of the RxxL motif encoded within the C43-4 peptide that recruits APC substrates to the

Cdh1/Apc10 co-receptor. We synthesized the D7A mutant version of C43-4 and showed that it localized to the nucleus (**Figure 9A**) and did not increase yeast CLS (**Figure 9E**). We feel this extra data requested by the reviewer adds mechanistic potential for the peptide and allowed us to generate a control that validates the use of C43-4 for increasing yeast CLS.

15. Figure 7, characterization of the peptides to show that their phenotypes are specific to the APC, should be inserted after Figure 3, as its current location in the paper disrupts the flow of the lifespan analyses in the second half of the paper. Also, Figure 8 (showing the 43-3 peptide activates the APC during the CLS) should be inserted after Fig. 5 (showing that the 43-3 peptide extends the CLS).

We have reorganized the flow of figures as suggested by the reviewer. The old Figure 7 was moved to the new Figure 4. The old Figure 4 moves to Figure 5. The old Figure 5 moves to new Figure 6. The old Figure 8 moves to new Figure 7. The old Figure 6A-D moves to new Figure 8. The new Figure 9 introduces the C43-4 mutant characterization. The old Figures 6F-M have been moved to Figure 10. And the old Figure 9 becomes new Figure 11. The text within the manuscript was been edited to reflect these changes.

16. Just a conceptual question for the authors to address in the discussion - the APC drives progression through M phase to G1 phase. As such, how do the authors imagine that the APC plays a role in quiescent / non dividing (G1/G0 phase) cells? Is the fact that the CLS assay requires a somewhat artifactual ability of the cells to form colonies again, i.e. grow again after leaving quiescence, be where the APC activation is playing a role, rather than during quiescence? If so, how does APC function relate to quiescence in the aging brain (after brain development and cell division is finished), where the cells will not be dividing again, regarding the statement "Therefore, the need for methods to target activation of APCCDH1 in the aging brain cannot be understated."

The APC^{CDH1} complex is required for cells to enter quiescence and to maintain it. APC^{CDH1} promotes cell repair that protects the cell from genomic instability and from developing cancer. Nondividing cells benefit from the ability of APC^{CDH1} to remain functional. We do not understand why the APC loses function in aging cells, but the defect is reversible, as observed in our studies, indicating it is a post-translational mark that is impaired likely due to loss of proper signalling. Our studies on another project indicate that APC activation by C43-4 and M2I-1 induce similar phosphorylation changes on proteins important for cell cycle progression, DNA repair, and cancer development and progression. Our recent observations (VanGenderen et al. 2026) show that C43-4 induces activating APC1 phosphorylation and increases APC ubiquitination activity. This indicates that C43-4 can restore the altered activity of multiple signaling pathways in cells due to impaired targeting of APC substrates for degradation. APC^{CDH1} targets many pro-cancer proteins for degradation. Therefore, loss of APC^{CDH1} activity could result in cancer because the many cancer causing kinases and transcription factors targeted by the APC would accumulate. By activating the APC, these proteins, and the growth promoting pathways downstream of

them, would be inhibited and result in phosphorylational changes. These concepts have been enhanced in the Discussion.

17. There is method section on measuring half-life with cycloheximide, but no results presented used this approach. Please remove.

We have added a new figure, Supplemental Figure 13C where cycloheximide is used. We have left this in the methods.

Reviewer #3 :

Harris et al. show that APC function declines as cells age and hypothesize that activating the APC/Cdh1 complex could block the transition from quiescence to senescence. This would extend chronological lifespan and represent a strategy for healthspan extension.By screening a set of previously identified Apc5- and Apc10-interacting peptides for APC activation, they identify peptide C43-3. Controlled expression of this peptide reduces levels of APC substrates Clb1 and Mps1 and increases chronological lifespan of yeast in an APC-dependent manner. Importantly, the effect is observed in both young and old yeast cells, implying that both a preventive and restorative effect on cellular fitness can be mediated by controlled (re)activation of APC-Cdh1. A synthetic C43-4 peptide also extends CLS, and a similar anti-aging effect is observed in worms expressing the peptide. The study is a thorough, large body of work, and the experiments are well executed. The identification of a small peptide activator of APC is a major advance. I have the following comments.

1. The RxxL motif in C43-3 is noteworthy for reasons pointed out by the authors. Mutagenesis of this motif in one experiment would strengthen the proposed model of how this peptide impacts APC and allow comments on the use of this peptide as a scaffold for future optimization. A synthetic mutant peptide or a mutant form of the yeast construct seems reasonably straightforward.

We have done this and added it to the manuscript. Three mutants in particular are important. We identified a C43-4 D7A mutation that disrupts the C43-4-Apc10 2-hybrid interaction and function. We synthesized this peptide and when added to cells, it is localized to the cell nucleus similar to the WT C43-4. We have used this peptide in addition to the scrambled peptide to show that D7A does not increase CLS. We made an R12A mutant that disrupts a conserved R in the peptide that alters the R in the RxxL motif. A third mutant changes the V within the xx domain to A. Both the R12A and V14A mutants disrupt C43-4 function as evidenced by the loss of the slow growth phenotype driven by the C43-4 peptide (**Figure 9D**). These mutants were made because these amino acids are shared between C43-4 and the yeast histone variant Htz1. We have included data showing that Htz1 interacts genetically with the APC, accumulates in APC mutants and when Htz1 is expressed, the normally unstable Apc10 accumulates. This data has also been added.

2. Considerable efforts to control experiments with a scrambled C43-3 (+TAT) peptide were made, which is commendable, but this peptide still fails to enter the nucleus. A C43-3-like peptide that enters the nucleus but does not interact with APC-Cdh1 would be a preferred control. In regard to this, a) what is Peptide 2 in Supp. Fig. 10B, and does it meet this criterion? b) Has a scrambled peptide been expressed in the TrxA vector? Addressing point 1 above would also address this issue, but with added value.

We have shifted towards using C43-4^{D7A} as our control peptide as is does exactly what the reviewer suggests a good control should do. Peptide 2 in the old manuscript was C43-4^{D7A} and that has now been revealed and expanded on. We have not expressed the scrambled peptide in the TrxA backbone. But the scrambled provides insight into how the peptide gets into the nucleus on its own as the 2 peptides have the same amino acid composition, but only C43-4 enters the cells. The arginines are clumped together in the WT peptide, suggesting they form some sort of nuclear localization sequence that is not present in the scrambled peptide. A reversed peptide has been ordered and will be used in future work. We expect this peptide will enter the nucleus as the arginine cluster is maintained.

3. How stable is the C43-4 peptide when expressed in yeast, and when applied exogenously to cultures? From Fig. 3f, the Trx fusion has a half-life in the range of hours, but the peptide addition experiments in Fig. 6C imply the peptide was added as the cultures grew to saturation. Does this mean the effective peptide concentration in the aging interval of days may in fact be much lower?

The peptide when expressed from the TrxA backbone is indeed unstable when tested in a cycloheximide experiment (not shown in this paper), consistent with the short half-life in the promoter shut off experiment in Figure 3G. We have observed this with every peptide expressed from the TrxA backbone we have used. We suspect it is due to the TrxA backbone, but do not currently have the clone to test this. We are currently performing a cycloheximide experiment on cells treated with the synthetic peptide. The synthetic peptide remains active throughout the CLS experiment suggesting it is either stable or its effects are long lasting. We have an ongoing study addressing how C43-4 stabilizes Apc10, as Htz1 does, so this concept of protein stability and C43-4 function is of major interest to us.

4. Is there a maximal benefit of APC activation that is capped by APC protein levels? Has a time course of APC subunit protein levels across chronological age been performed?

We have run a cycloheximide arrest release experiment with TAP-tagged APC subunits. We have observed that Apc10-TAP and Cdc27-TAP are both unstable, while the remaining subunits are stable. Interestingly, C43-4 stabilized Apc10-TAP, but not Cdc27. We feel the instability of Apc10 and Cdc27 may impair APC function over time, with the stabilization of Apc10-TAP by C43-4 reversing this. This data is being prepared for another manuscript.

Regarding protein levels of APC substrates, we have run westerns on aging cells in stationary phase against Clb1-TAP and Mps1-TAP in normal untreated cells. These substrates are acted upon by both Cdc20 and Cdh1. The proteins decline over time but do not disappear. So, we would expect that the proteins would go down further in the presence of C43-4. We have not yet tested this with C43-4 treatment. We have yet to test whether APC subunits remain stable during stationary phase, but expect they do as Apc2, Apc4, Apc5, Apc11 and Cdc16 are all stable in CHX experiments. This data will be presented elsewhere.

5. The legend of Fig. 6 is out of order with the data panels.

This has been fixed. The old Figure 6 has now been split into new Figures 8 and 10.

6. Please clarify which experiments involved the CHX pulse-chase described in the Methods.

The methods describing the CHX experiment refer to the new Supplemental Figure 13C. The old version did not contain a CHX experiment. That was my oversight.

August 28, 2026
RE: GENETICS-2026-309832

Dr. Troy Anthony Alan Harkness
University of Alberta
Medicine
320 Heritage Medical Research Center
Edmonton Canada
Canada

Dear Dr. Harkness:

Congratulations, your manuscript titled "Discovery of a small peptide that increases yeast lifespan by enhancing the function of APCCdh1 in nondividing quiescent cells" is accepted for publication in GENETICS! Many thanks for submitting your research to the journal.

To Proceed to Publication:
1. Format your article according to GENETICS style: https://academic.oup.com/genetics/pages/author-guidelines

2. Ensure that you comply with data and community resource citation guidelines:
https://academic.oup.com/genetics/pages/author-guidelines#section-5-9-2

3. Upload your final files at https://genetics.msubmit.net

4. Add oupsupport@scipris.com and genetics.oup@novatechset.com (or the domains @scipris.com and @novatechset.com) to your email program's "safe senders" list. You will be contacted by both at various points during the production process.

Notes:
- Your currently-accepted manuscript (unedited, as submitted, reviewed, and accepted) will be published at GENETICS and deposited into PubMed as an Advance Access article. Notify sourcefiles@thegsajournals.org before signing your license if you do not wish to publish your article via Advance Access.

- We invite you to submit an original color figure related to your paper for consideration as cover art. Please note that submissions created with AI image generation tools are not eligible. Please email your submission to the editorial office or upload it with your final files. You can submit a small-sized image for evaluation, and if selected, the final image must be a TIFF file 2513px wide by 3263px high (8.375 by 10.875 inches; resolution of 600ppi). Please avoid graphs and small type.

- After files are sent to Oxford University Press we use SciPris to manage article licensing and payment. If you do not have a SciPris account, you will receive an email from no-reply@scipris.com to sign up to use Oxford University Press' author portal. After logging in, follow the online instructions to sign your license and arrange any payment due.

If you have any questions or encounter any problems while uploading your accepted manuscript files, please email the editorial office at sourcefiles@thegsajournals.org.

Sincerely,

Soni Lacefield
Associate Editor
GENETICS

Approved by:
Meera Sundaram
Senior Editor
GENETICS

Review comments (if applicable):

Reviewer #3 :

The authors are commended on the effort made to address reviewers comments and the addition on Fig 9 strengthens the paper with important mechanistic details. I have no further suggestions.
