# Supplementary material for: Discovery of a small peptide that increases yeast lifespan by enhancing the function of APCCdh1 in nondividing quiescent cells
Source: Genetics. 2026 Sep 16;234(3):iyag249. doi: 10.1093/genetics/iyag249 (PMC13732707; doi:10.1093/genetics/iyag249)
Supplement: Supplementary file 2 [file iyag249_supplementary_data.pdf]

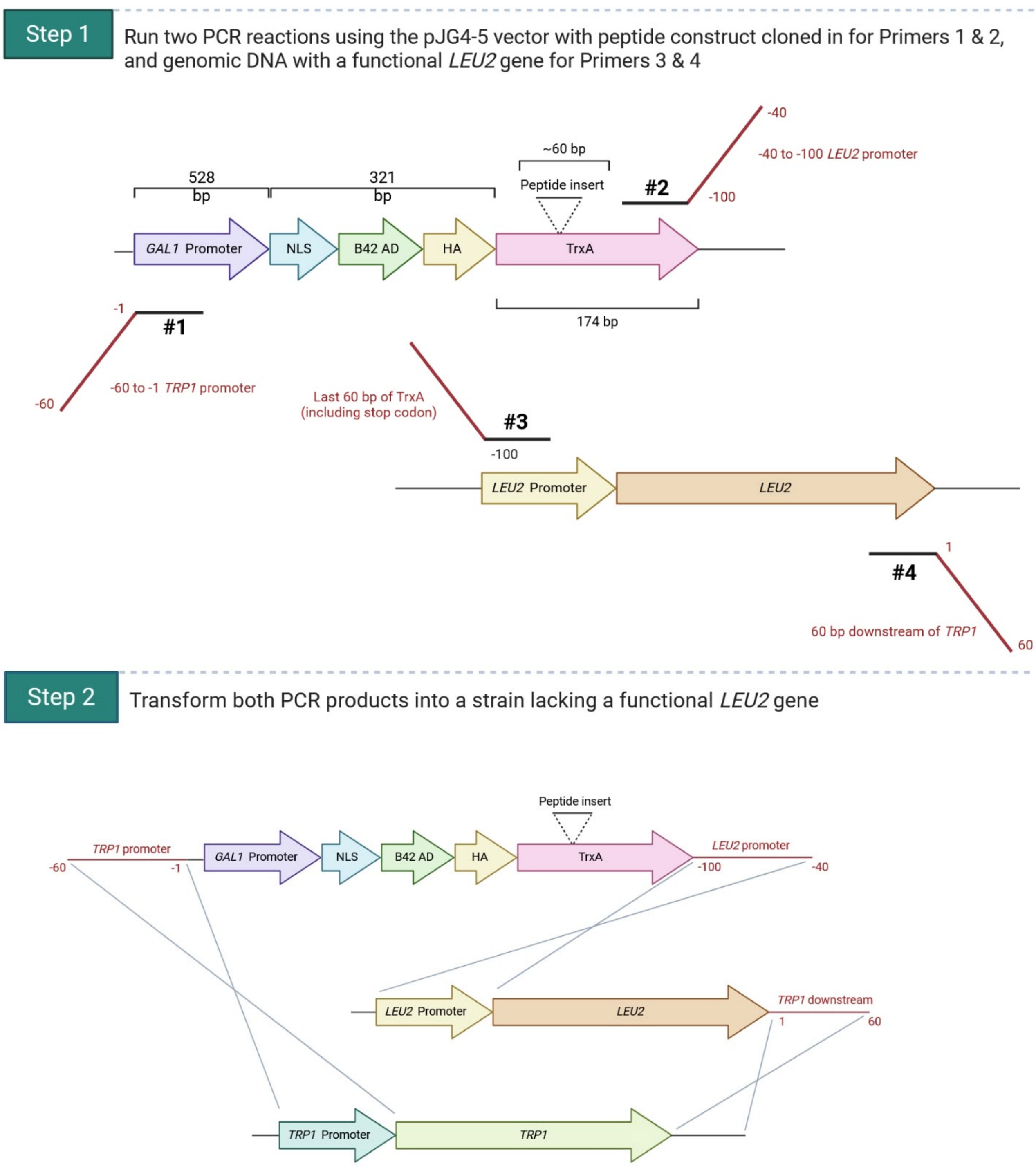

Figure S1. Schematic for integration of peptide constructs into the yeast genome. See Table 3 for primer sequences.

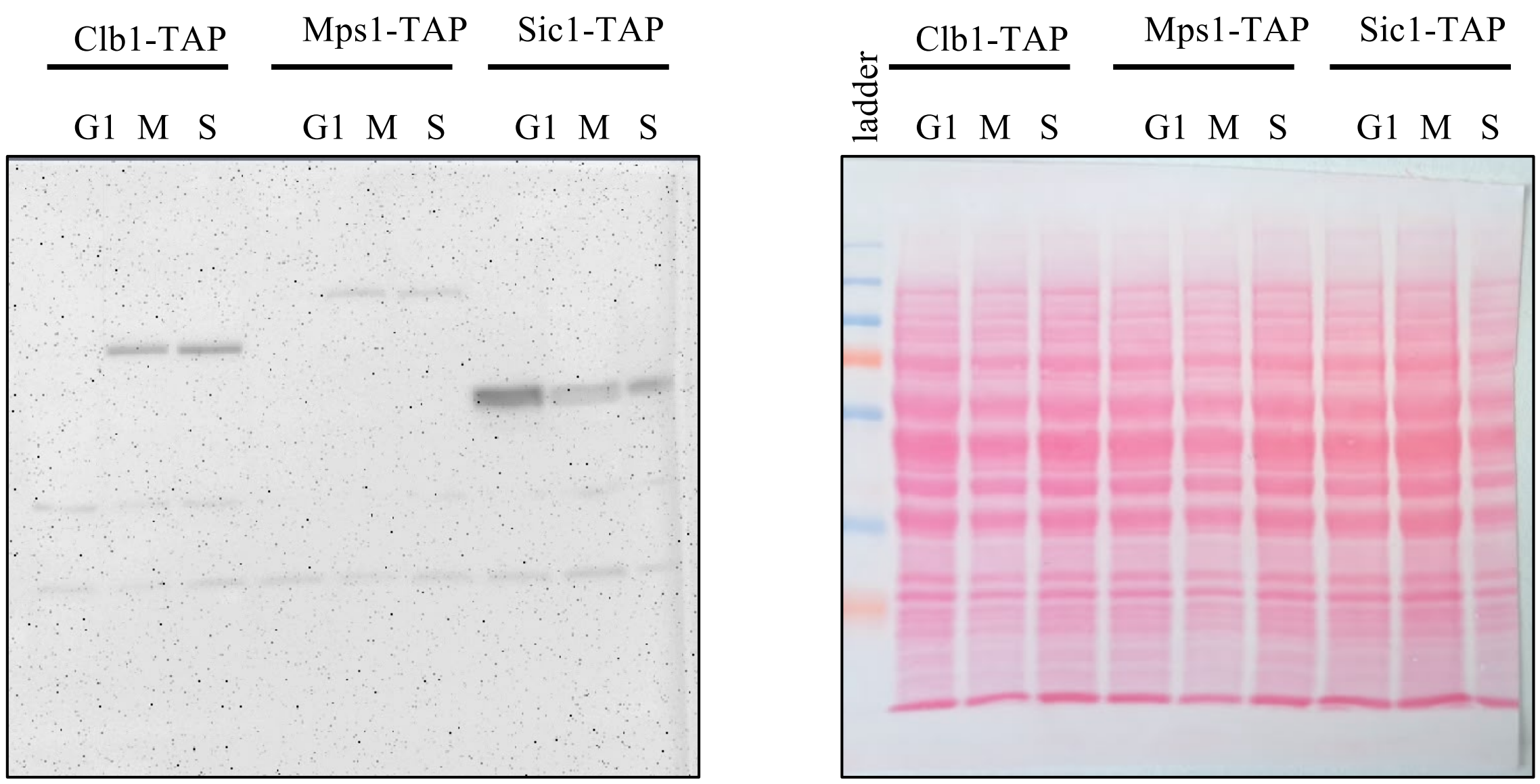

Figure S2. Uncropped blots for westerns in Fig. 1C. Yeast trains expressing Clb1-TAP, Mps1-TAP, or Sic1-TAP were grown to mid-log phase, then treated with either α−factor, nocodazole, or hydroxyurea to arrest the cells in G1, M or S phase, respectively.

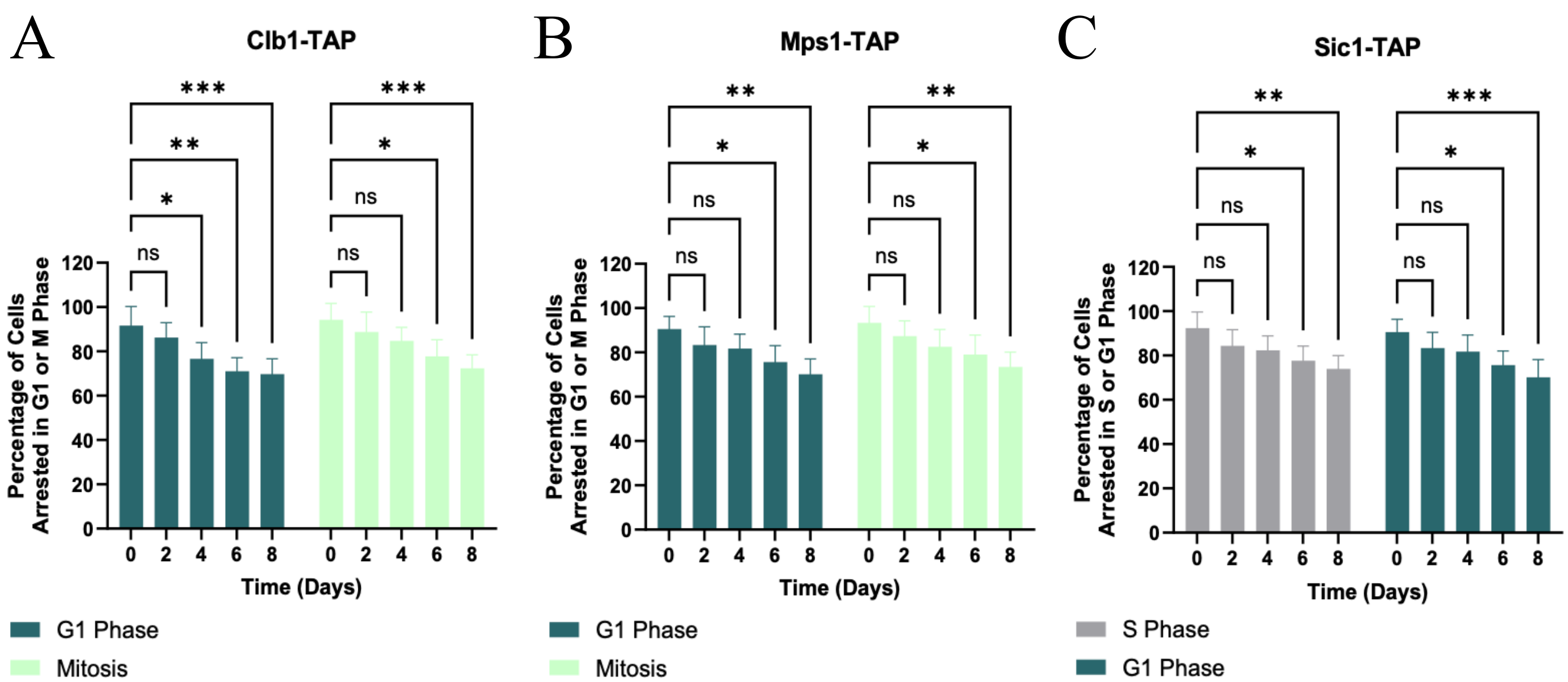

Figure S3. Budding index of cells used in Fig. 1. (A) Aging CLS cells expressing Clb1-TAP were re-introduced into the cell cycle using fresh YPD media containing nododazole to arrest cells in the next mitosis, or α−factor to arrest them in the next G1. Cells at the days shown were counted for arrested cells. **(B)** Aging CLS cells expressing Mps1-TAP were treated as in **(A). (C)** Aging CLS cells expressing the SCF substrate Sic1-TAP were re-introduced back into the cell cycle as above, in the presence of α−factor to arrest cells in G1 or hydroxyurea to arrest cells in S phase. Cells were counted at each time point for arrested cells.

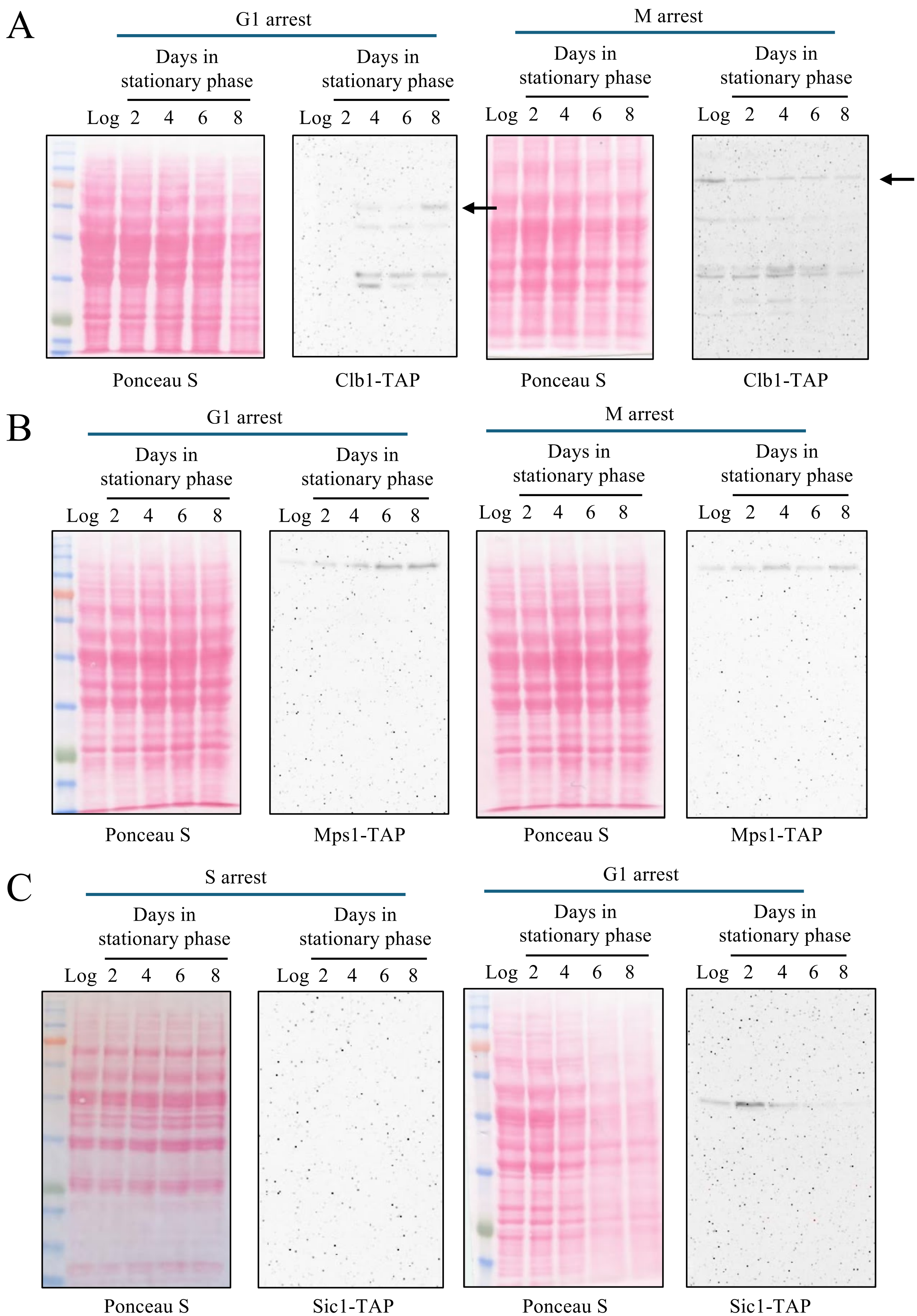
A
G1 arrest
Days in stationary phase
Log 2 4 6 8
Ponceau S
Days in stationary phase
Log 2 4 6 8
Clb1-TAP
M arrest
Days in stationary phase
Log 2 4 6 8
Ponceau S
Days in stationary phase
Log 2 4 6 8
Clb1-TAP
B
G1 arrest
Days in stationary phase
Log 2 4 6 8
Ponceau S
Days in stationary phase
Log 2 4 6 8
Mps1-TAP
M arrest
Days in stationary phase
Log 2 4 6 8
Ponceau S
Days in stationary phase
Log 2 4 6 8
Mps1-TAP
C
S arrest
Days in stationary phase
Log 2 4 6 8
Ponceau S
Days in stationary phase
Log 2 4 6 8
Sic1-TAP
G1 arrest
Days in stationary phase
Log 2 4 6 8
Ponceau S
Days in stationary phase
Log 2 4 6 8
Sic1-TAP

Figure S4. Uncropped blots for Figures 1C-F. (A) Cells expressing Clb1-TAP were grown to stationary phase. Samples were obtained after 2, 4, 6, and 8 days of aging, and added back to YPD media to allow the cells to re-enter the cell cycle. α−factor or nocadozole was added to arrest cells in the first G1 or mitosis encountered. **(B)** Cells expressing Mps1-TAP were treated as in **(A)**. **(C)** Cells expressing Sic1-TAP were treated as in **(A)**, except that cells re-entered the cell cycle in the presence of hydroxyurea or α−factor, to arrest in S or G1. Lysates were prepared from all samples with westerns performed using antibodies against the TAP epitope. All membranes were stained with Ponceau S to control for protein load.

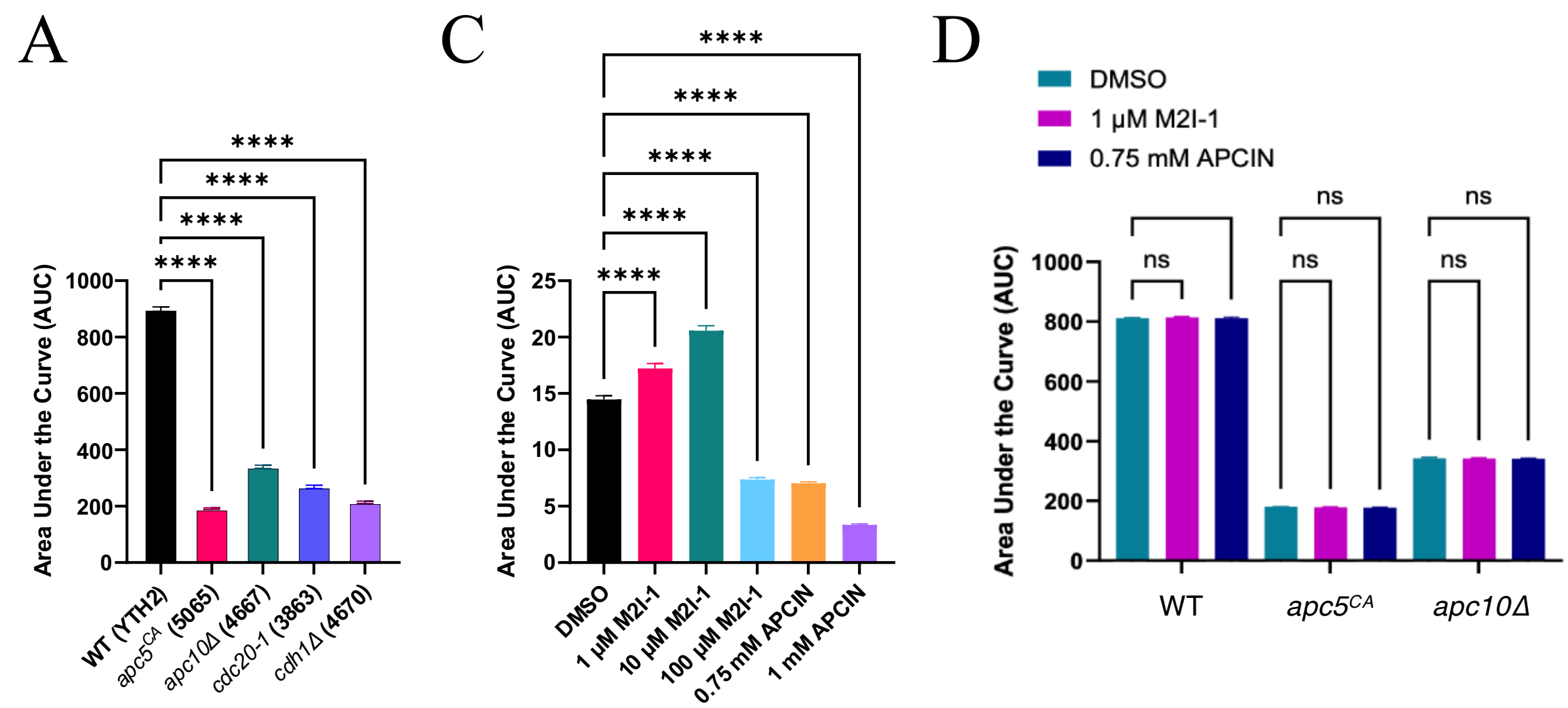

B

Strain	Mean Survival (Days)	95% Confidence Interval	Max survival <10% (Days)
WT	13.26 ± 0.09225	12.93 to 13.61	23
apc5^{CA}	3.80 ± 0.6632	3.606 to 3.988	7
apc10Δ	6.25 ± 0.3796	5.558 to 6.912	15
cdc20-1	5.20 ± 0.5098	4.520 to 5.819	13
cdh1Δ	4.02 ± 0.5953	3.830 to 4.219	7

Figure S5. Quantitation for CLS assays of APC mutants and cells treated with M2I-1 or Apcin. (A) WT and APC mutants were used for methylene blue CLS assays. Area under the curve (AUC) was determined for each curve shown in **Figure 2A**. **(B)** A statistical analysis for the curves shown in **Figure 2A** is provided. **(C)** Cells treated with the doses of M2I-1 or Apcin shown were used for CLS using the methylene blue assay. AUC is shown. Linked with **Figure 2B**. **(D)** WT, *apc5^{CA,}* and *apc10*Δ cells were treated with 1 μM M2I-1 or 0.75 mM Apcin and used for CLS measured using methylene blue staining. AUC is shown. Linked with. **Figure 2E**

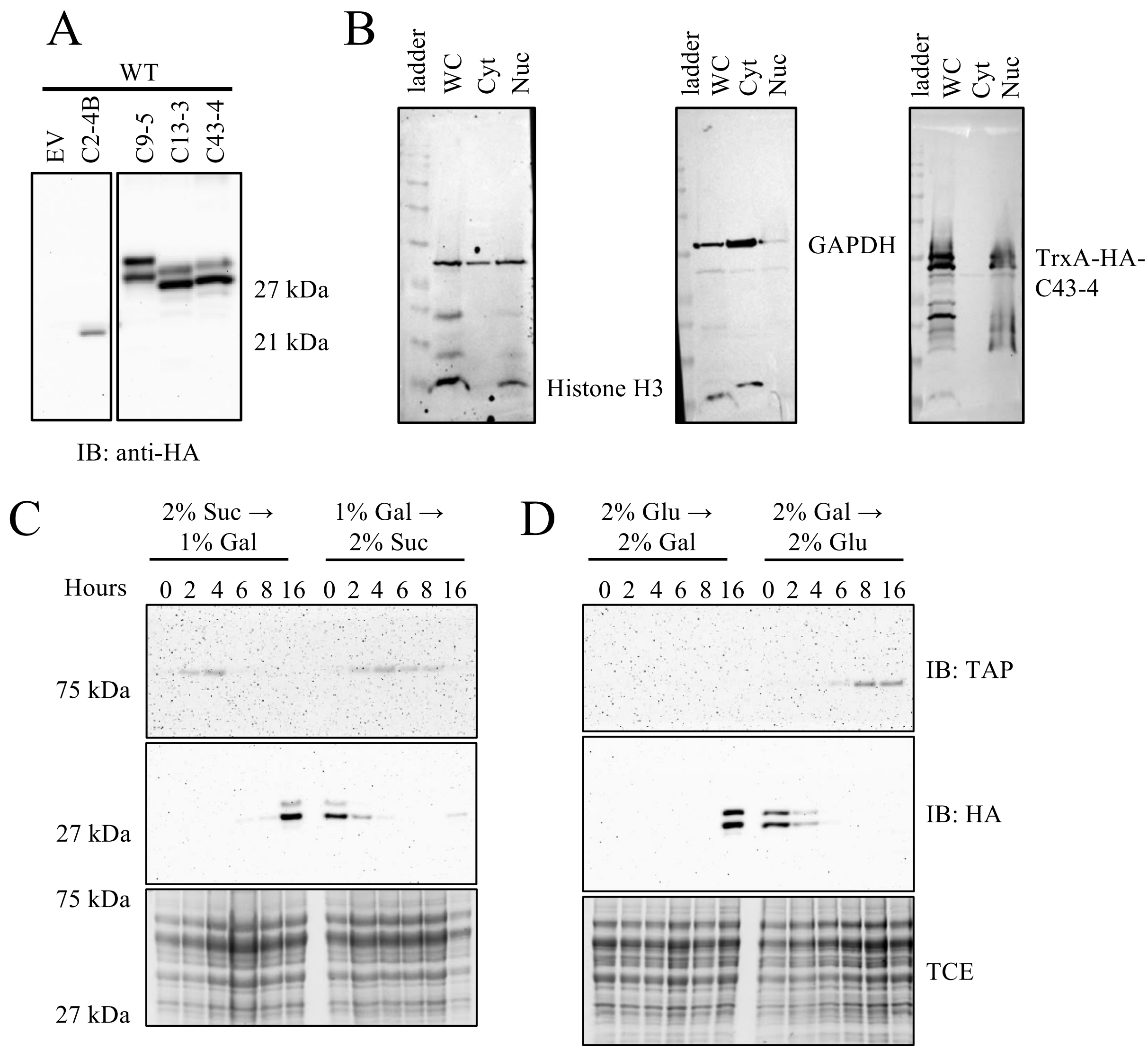

Figure S6. Uncropped blots for westerns shown in Figures 3E-G. (A) Cells expressing plasmid-borne HA-tagged peptides were induced with galactose, then prepared for westerns with antibodies against HA. Linked with **Figure 3E**. **(B)** Nuclear fractionation of cells expressing plasmid-borne HA-C43-4 was performed. Cells were fractionated into cytosolic or nuclear fractions and compared to whole cell lysates. Antibodies against GAPDH (cytosol), H3 (nucleus), and HA were used in westerns. Linked with **Figure 3F**. **(C)** Cells expressing Clb1-TAP and the integrated C43-4-HA construct were grown in 2% sucrose supplemented media overnight, then shifted to 1% galactose for the times shown. After galactose induction, the cells were washed and grown in media containing 2% sucrose for the times shown to stop galactose induction. Lysates were prepared and assessed using antibodies against TAP and HA. Linked with **Figure 3G**. **(D)** The same experiment and cells shown in **(C)** were used, except rather than 2% sucrose, the cells were grown in 2% glucose before and after 1% galactose induction. Linked with. **Figure 3G**

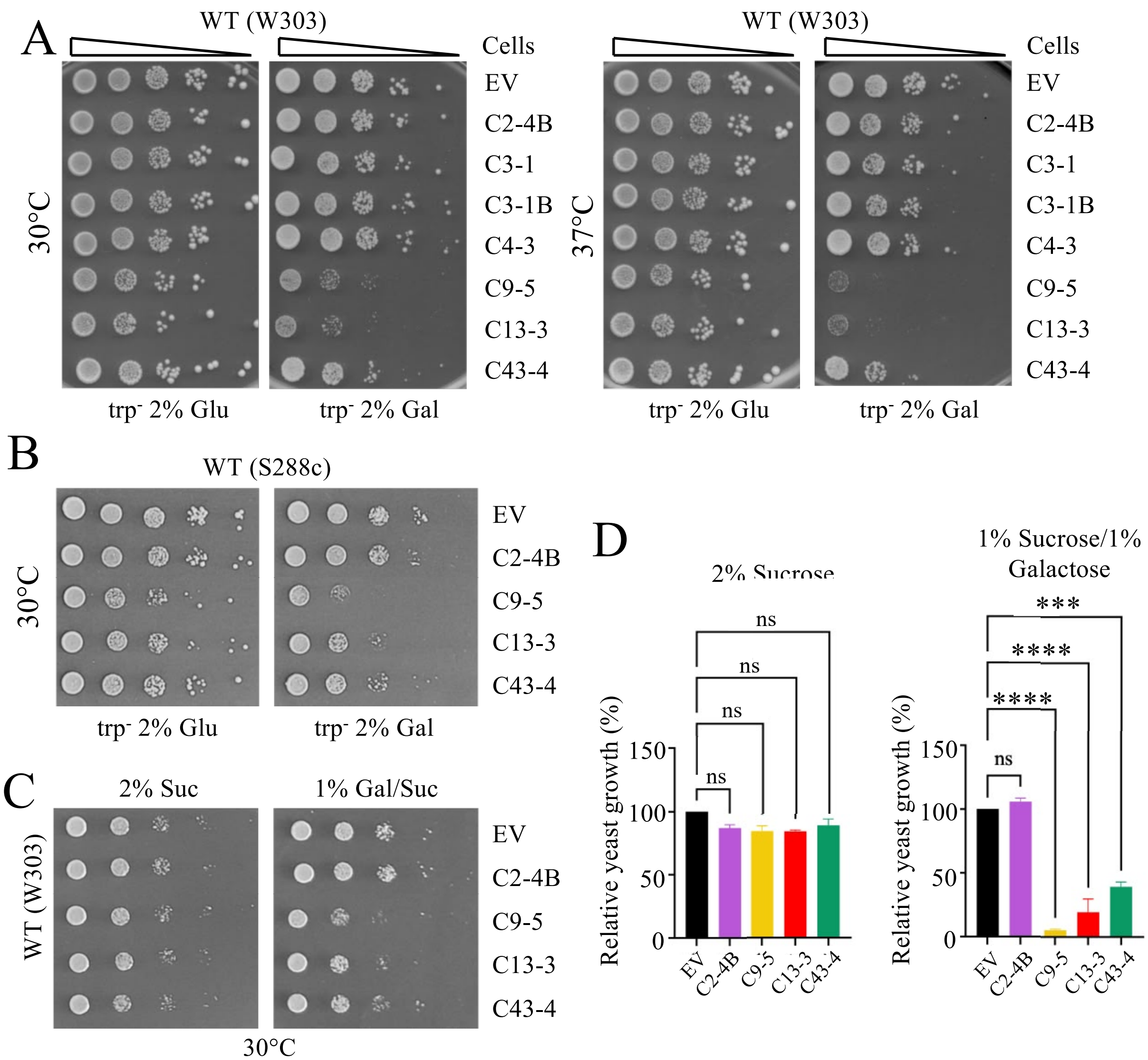

Figure S7. Growth phenotypes of cells expressing the various plasmid-borne peptide constructs. (A) W303-based WT cells were transformed with the peptide constructs shown. 10-fold serial dilutions were spotted onto trp- media to select for the plasmids that contained either 2% glucose to repress peptide expression, or 2% galactose to induce expression. Plates were grown at 30° and 37° for 2 days, then scanned. **(B)** S288c-based WT cells were transformed with the peptide constructs shown, then spot diluted as described in **(A)**. Cells were grown at 30°C on trp- plates supplemented with either 2% glucose or 2% galactose. **(C)** W303-based WT cells expressing the peptides shown were grown on trp- plates supplemented with either 2% sucrose or a mixture of 1% sucrose and 1% galactose. The plates were maintained at 30°C for 2 days, then scanned. **(D)** The spots shown in **(C)** were scanned with densitometry performed using Image J to determine the density of the spots in the third column from the left. The results were graphed.

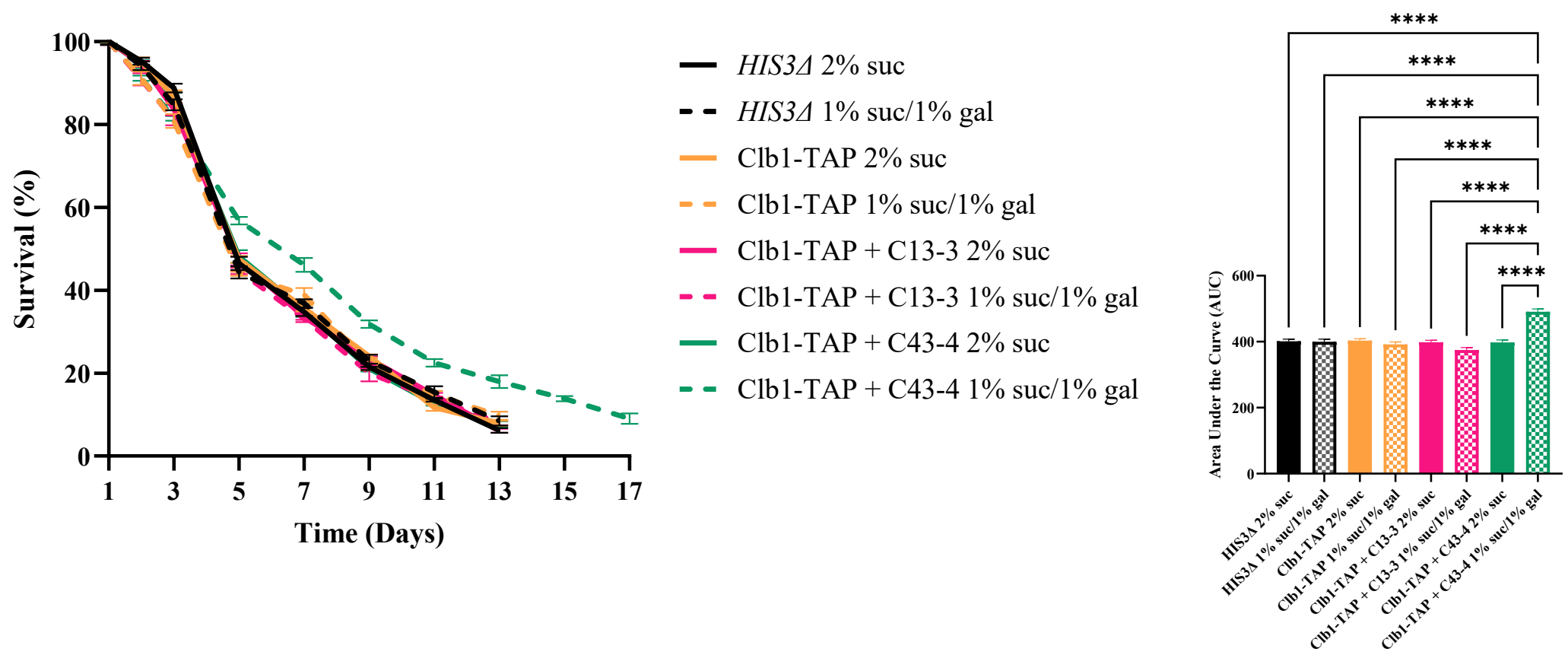

Figure S8. An integrated copy of C43-4 extends CLS, while an integrated copy of C13-3 does not. Clb1-TAP cells containing integrated copies of C43-4-HA or C13-3-HA were grown to stationary phase in complete media supplemented with either 2% sucrose or 1% sucrose/1% galactose. The cells were grown to stationary phase and maintained in the depleted media until cell viability was below 10%. Samples were removed every other day and assessed for viability using the methylene blue method. Area under the curve was determined for each curve and plotted on the right.

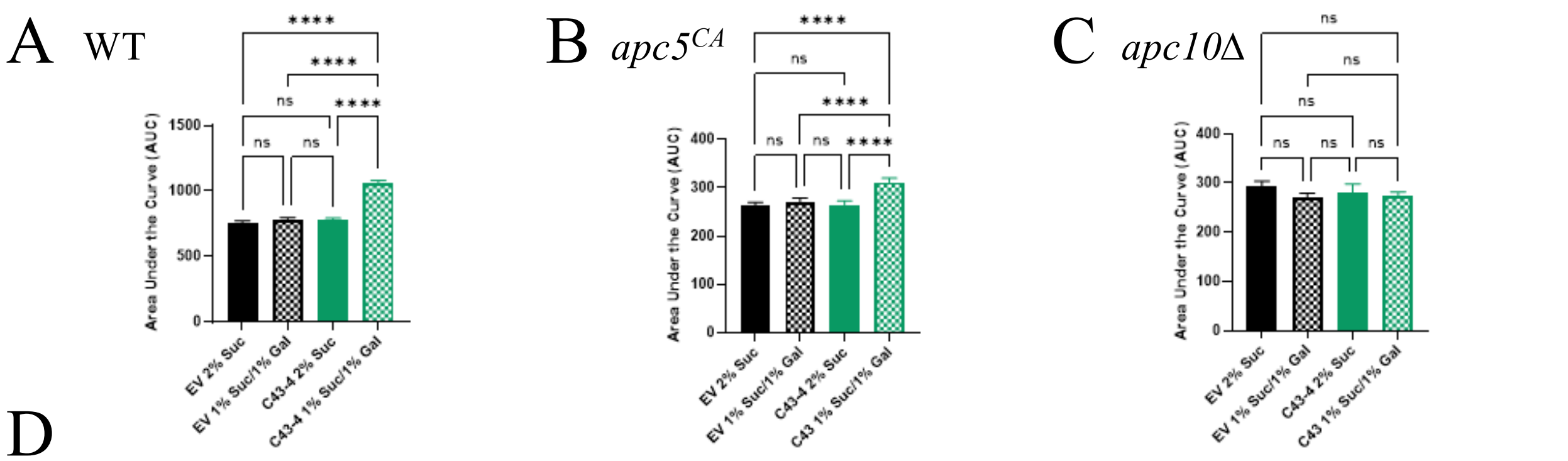

D

Strain	Mean Survival (Days)	95% Confidence Interval	Max survival (Days)
**WT EV 2% Suc**	10.73 ± 0.1151	10.41 to 11.06	21
**WT EV 1% Gal**	10.92 ± 0.1049	10.62 to 11.22	21
**WT C43 2% Suc**	10.92 ± 0.1171	10.59 to 11.25	21
**WT C43 1% Gal**	14.38 ± 0.05722	14.10 to 14.67	27
***apc5^{CA}* EV 2% Suc**	4.209 ± 0.7631	3.977 to 4.445	7
***apc5^{CA}* EV 1% Gal**	4.254 ± 0.8992	3.977 to 4.539	7
***apc5^{CA}* C43 2% Suc**	4.303 ± 0.8374	4.041 to 4.574	7
***apc5^{CA}* C43 1% Gal**	5.011 ± 0.9553	4.538 to 5.485	9
***apc10Δ* EV 2% Suc**	4.834 ± 0.4333	4.611 to 5.062	9
***apc10Δ* EV 1% Gal**	4.634 ± 0.4421	4.418 to 4.852	9
***apc10Δ* C43 2% Suc**	4.577 ± 0.5863	4.298 to 4.857	9
***apc10Δ* C43 1% Gal**	4.659 ± 0.5458	4.384 to 4.937	9

Figure S9. C43-4 CLS extension requires a functional APC complex. Linked with Figure 6H. (A) WT W303-based cells were transformed with an empty vector (EV) or C43-4-HA. Cells were maintained on media supplemented with either 2% sucrose or 1% sucrose/1% galactose. AUC was measured for each curve and plotted. **(B)** W303-based *apc5^{CA}* cells were treated as in **(A)**. AUC was determined for each CLS curves and plotted. **(C)** W303-based *apc10*Δ cells were treated as in **(A)**. AUC was determined for each CLS curves and plotted. **(D)** Stats for each experiment were determined.

Supplemental Figure 10

A

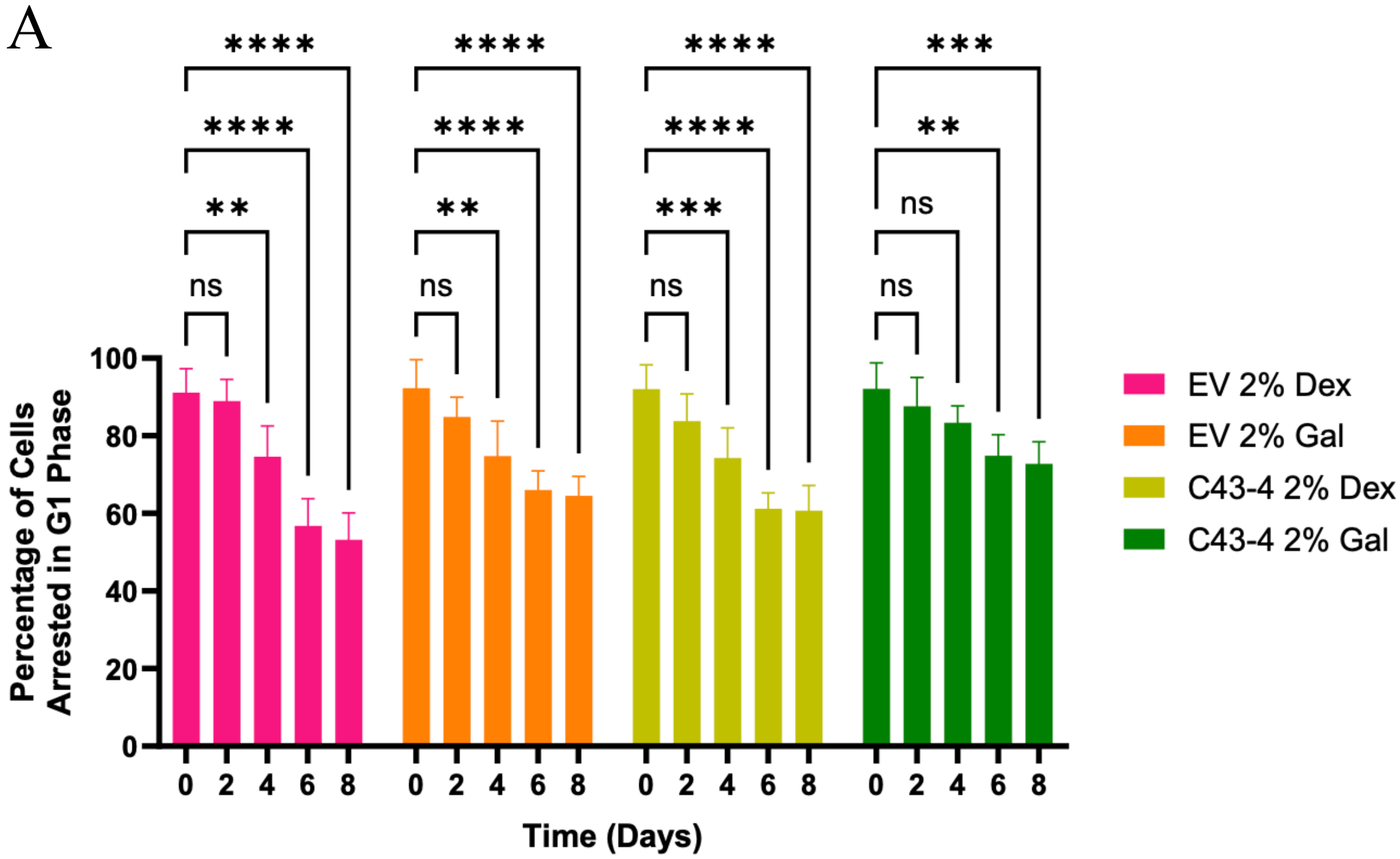

B

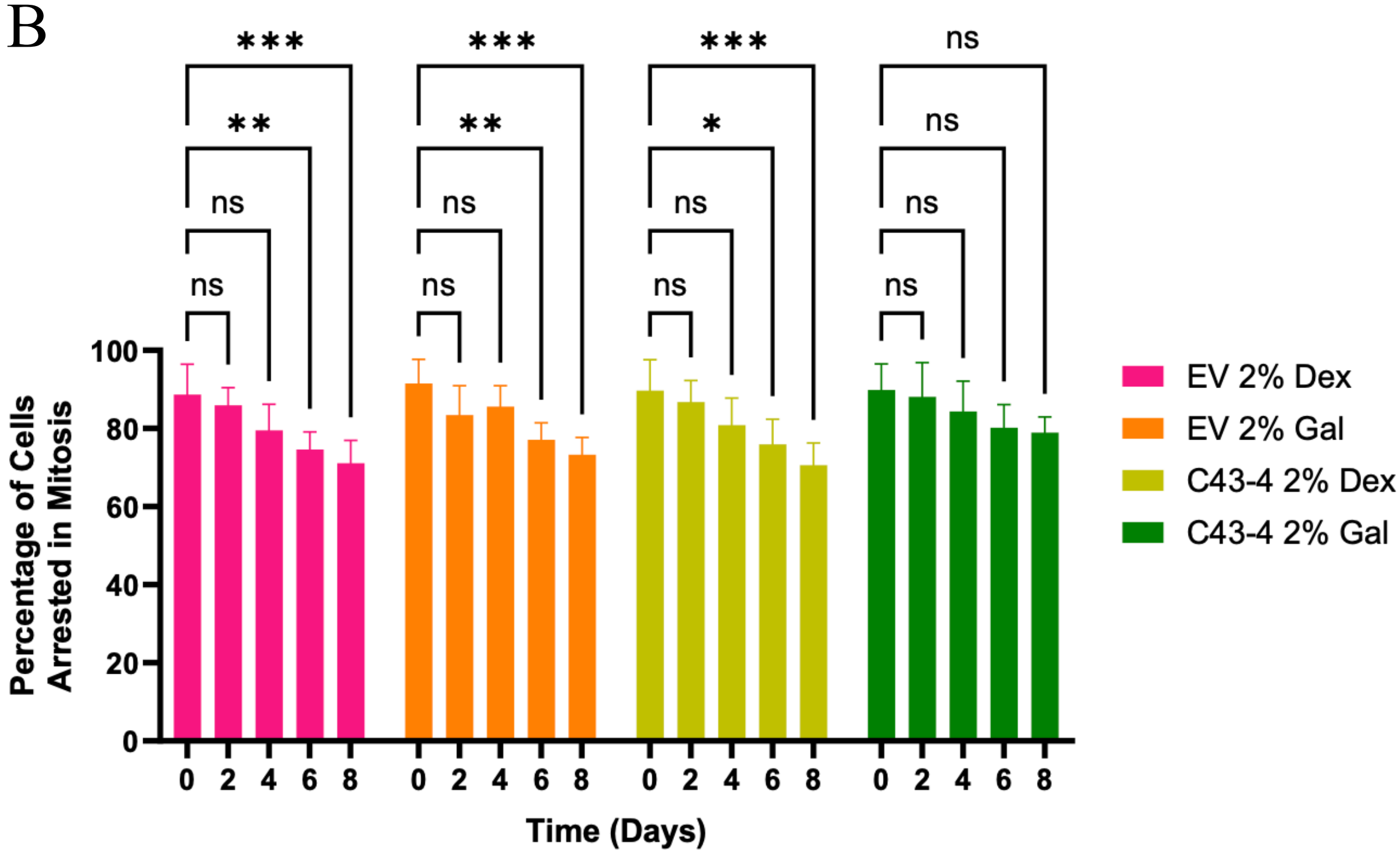

Figure S10. Budding index of cells used in Figure 7. (A) Cells expressing Clb1-TAP harboring an integrated C43-4-HA, or Clb1-TAP cells transformed with an EV, were maintained in depleted MM-trp to age. Media contained either 2% glucose or 2% galactose. Cells were re-introduced back into the cell cycle every other day. The YPD media contained α−factor to arrest them in the first G1 they encountered. Cells at each timepoint were scored for the number of cells arrested in G1 and plotted. **(B)** Cells as above were aged then re-introduced back into the cell cycle using YPD media supplemented with nododazole to arrest cells in the first mitosis they encountered. Cells at the days shown were scored for cells arrested in mitosis.

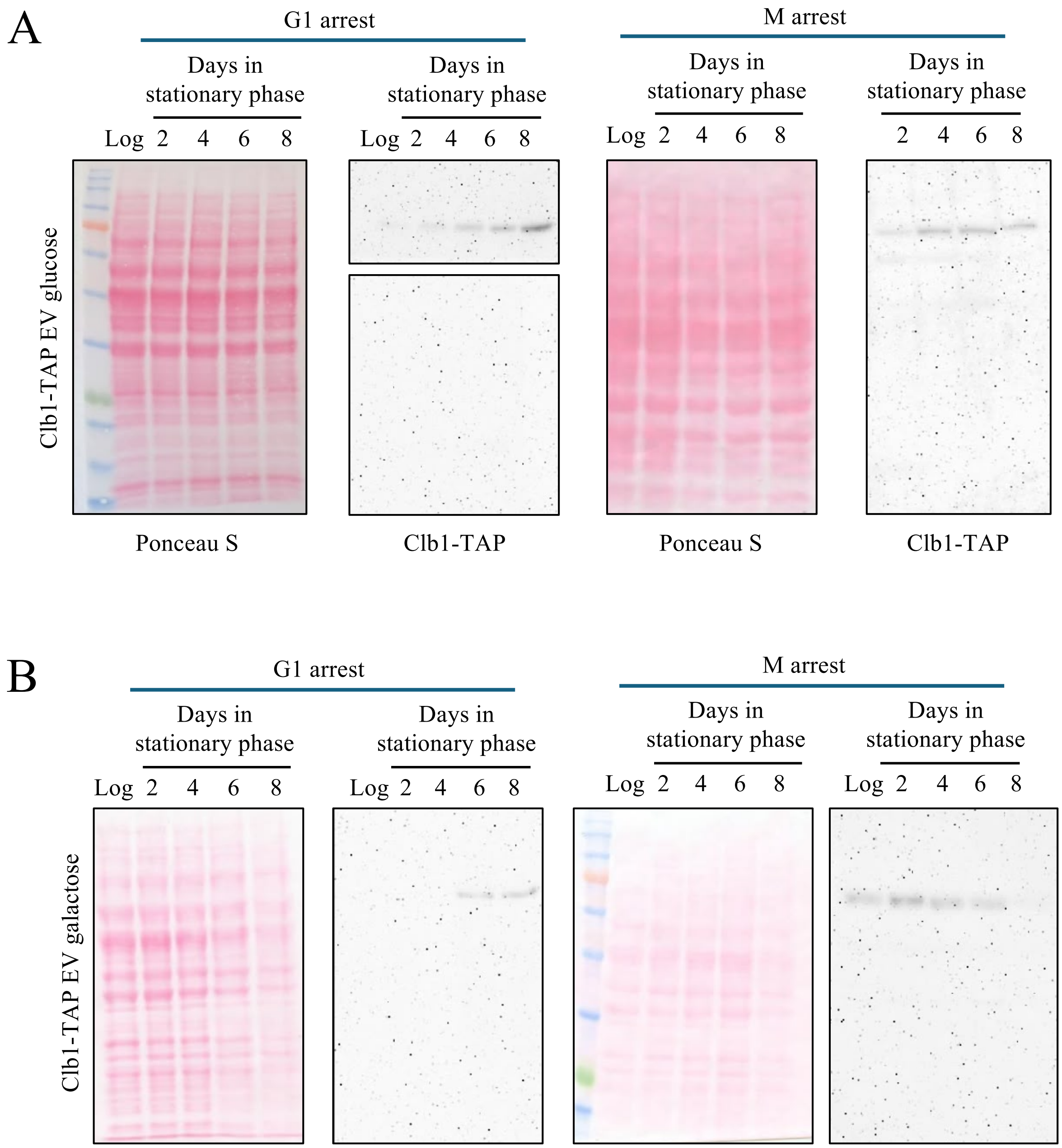
A
G1 arrest
M arrest
Days in stationary phase
Log 2 4 6 8
2 4 6 8
Clb1-TAP EV glucose
Ponceau S
Clb1-TAP
B
Clb1-TAP EV galactose

C

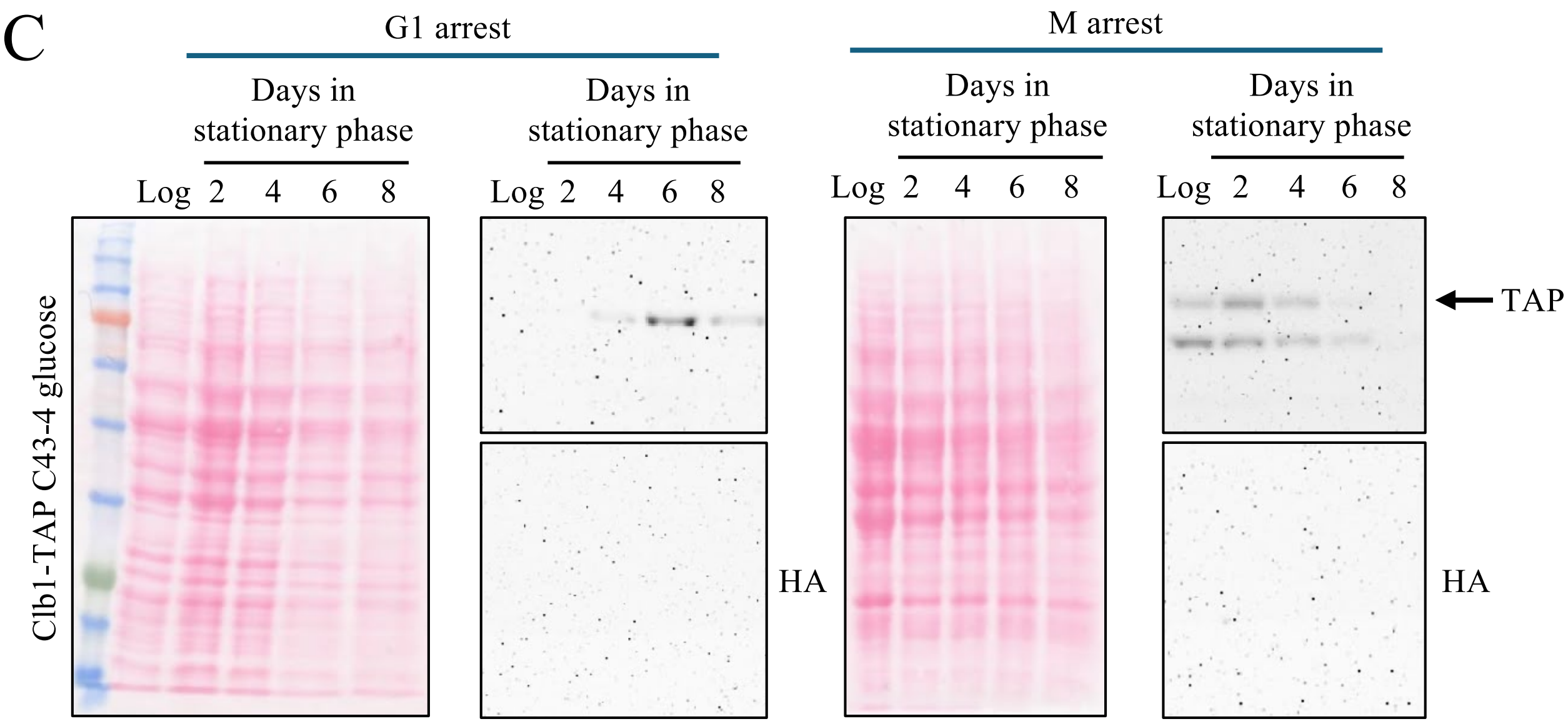
G1 arrest
M arrest
Days in stationary phase
Days in stationary phase
Days in stationary phase
Days in stationary phase
Log 2 4 6 8
Log 2 4 6 8
Log 2 4 6 8
Log 2 4 6 8
Clb1-TAP C43-4 glucose
TAP
HA
HA

D

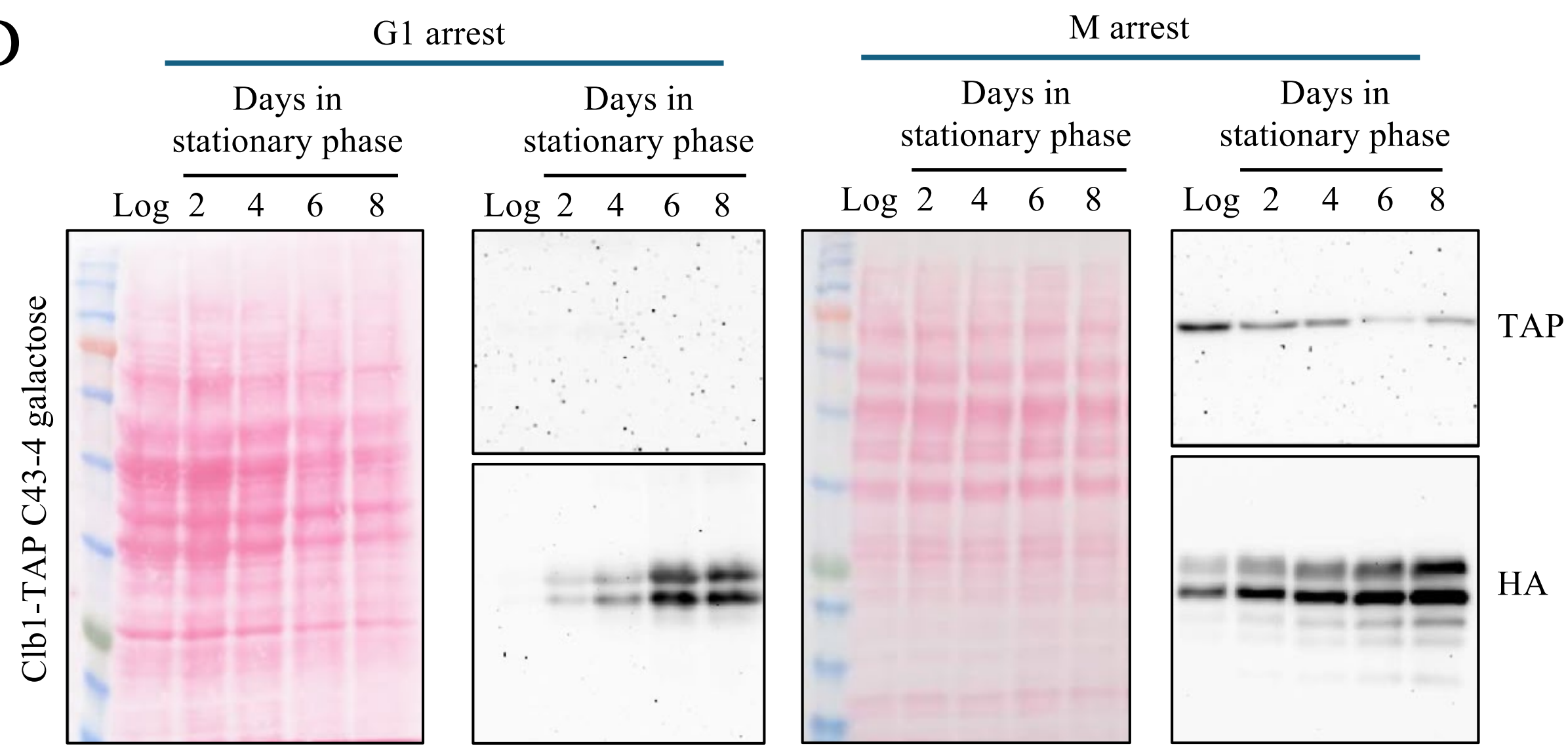
G1 arrest
M arrest
Days in stationary phase
Days in stationary phase
Days in stationary phase
Days in stationary phase
Log 2 4 6 8
Log 2 4 6 8
Log 2 4 6 8
Log 2 4 6 8
Clb1-TAP C43-4 galactose
TAP
HA

Figure S11. Uncropped blots for westerns shown in Figure 7. (A) Clb1-TAP cells transformed with an empty vector were maintained in depleted MM-trp supplemented with 2% glucose to age. Cells were re-introduced back into the cell cycle using YPD media supplemented with either α−factor or nocodazole at the days shown. Lysates were prepared and assessed using antibodies against the TAP or HA motifs. Linked with **Figure 7A**. **(B)** Clb1-TAP cells transformed with an empty vector were treated as above, except they were maintained in media containing 2% galactose. Cells were re-introduced back into the cell cycle, as described above. Lysates were prepared and assessed using antibodies against the TAP or HA motifs. Linked with **Figure 7B**. **(C)** Clb1-TAP cells containing an integrated C43-4-HA were maintained in depleted MM supplemented with 2% glucose to age. Cells were re-introduced back into the cell cycle using YPD media supplemented with either α−factor or nocodazole at the days shown. Lysates were prepared and assessed using antibodies against the TAP or HA motifs. Linked with **Figure 7C**. **(D)** The cells used in **(C)** were treated as above except they were maintained in media supplemented with 2% galactose. They were re-introduced back into the cell cycle as described. Lysates were prepared and analyzed using antibodies against TAP or HA. Ponceau S stained membranes served as load controls.

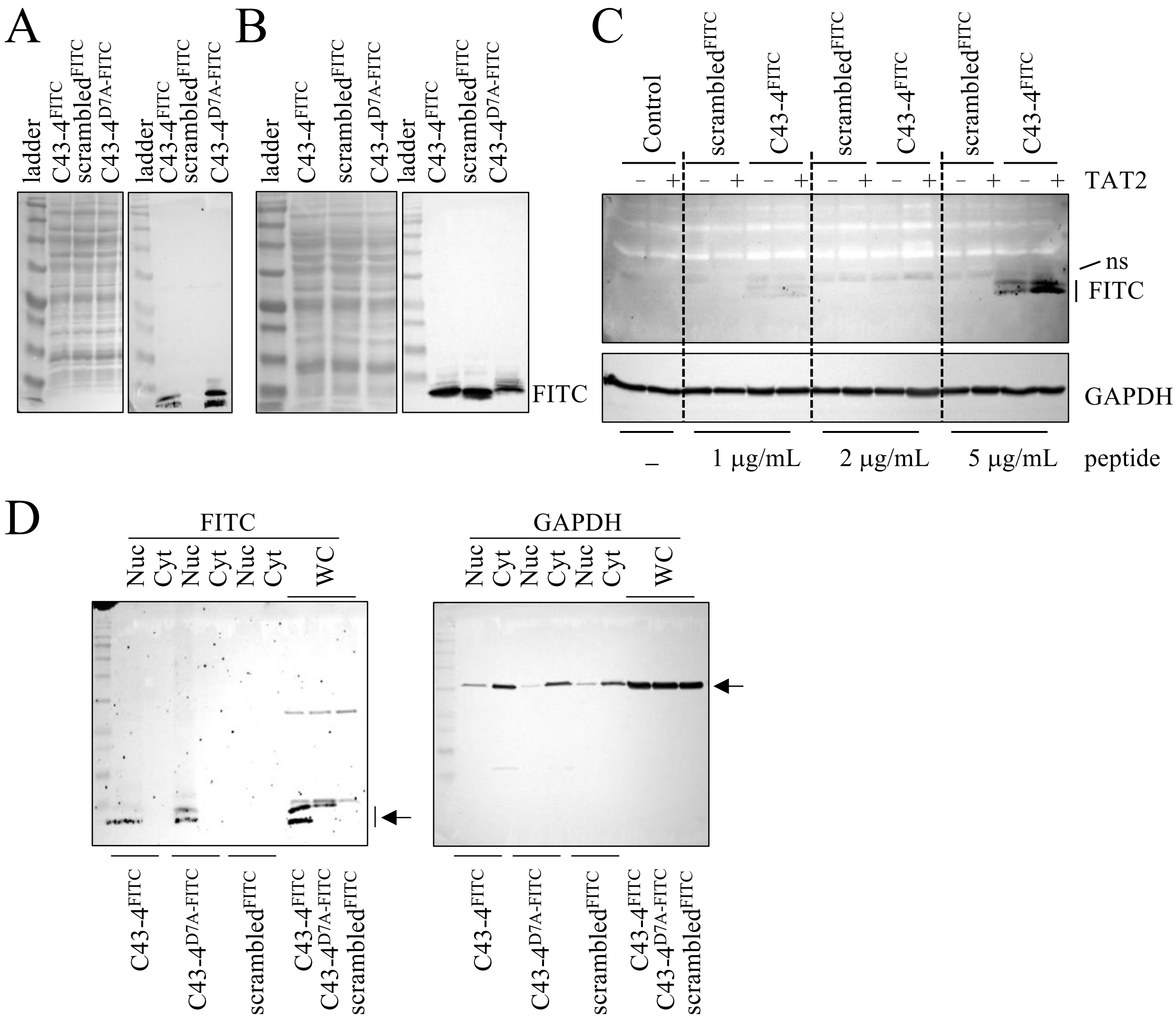

Figure S12. Uncropped blots detailing localization of C43-4^{FITC} and its controls, C43-4$^{D7A\text{-}FITC}$ and scrambled^{FITC}. (A) Cells were grown overnight with 5 µg/ml of each peptide added to the culture. The next morning, lysates were prepared and westerns were performed using antibodies against the FITC motif. **(B)** The experiment shown in **(A)** was repeated, except 1 ug of C43-4^{FITC} or scrambled^{FITC} were spiked into the lysates. Westerns were performed using antibodies against FITC. **(C)** Cells were grown overnight containing either 1, 2 or 5 ug/ml of either C43-4^{FITC} or scrambled^{FITC}, or left untreated. The peptides were coupled to the membrane permeable TAT2 or left uncoupled. The next day lysates were prepared and used for westerns with FITC antibodies. Antibodies against GAPDH were used as a load control. **(D)** Uncropped blots for nuclear fractionation of cells growing in the presence of C43-4^{FITC}, C43-4$^{D7A\text{-}FITC}$ or scrambled^{FITC}. Antibodies against FITC or GAPDH were used. Linked with **Figure 9A**.

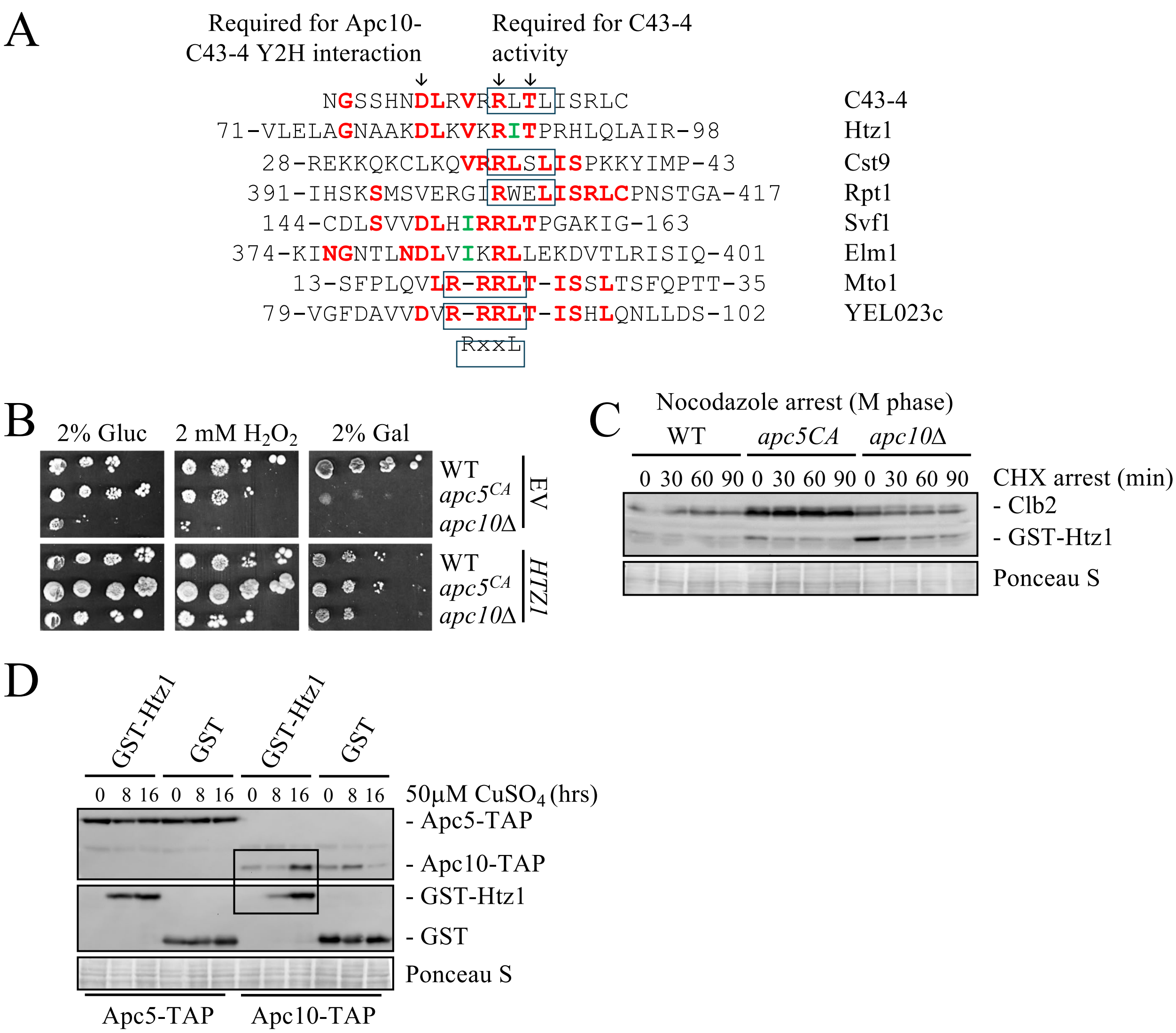

Figure S13. The stress responsive histone variant, Htz1, shares sequence homology with C43-4 and genetically and functionally interacts with the APC. (A) Results from a BLAST of the C43-4 peptide sequence against the yeast proteome. **(B)** *Overexpression of galactose inducible GST-HTZ1 suppresses apc5^{CA} and apc10Δ defects*. WT, *apc5^{CA}*, and *apc10*Δ cells expressing galactose inducible *GST-HTZ1* were spot diluted onto plates containing 2% glucose, H_2O_2, or 2% galactose. Plates were incubated at 30°C for 3 days. **(C)** *Htz1 is targeted by the APC for degradation*. *GST-HTZ1* was induced with 0.5% galactose in the cells shown for 16 hours. Nocodazole was added for 3 hours to arrest the cells in mitosis. Cycloheximide (CHX) was then added to inhibit protein synthesis with cells removed at the times shown to assess protein stability using antibodies against endogenous Clb2 or GST. **(D)** *Htz1 stabilizes Apc10.* Apc5-TAP or Apc10-TAP cells expressing Gal inducible *GST-HTZ1*, or the GST empty vector, were grown to mid log phase. 0.5% galactose was added for the time shown followed by GST and TAP westerns. The boxed area highlights induction of Apc10-TAP in response to increasing Htz1 levels.

Supplemental Table 1. Data for **Figure 2C** using OASIS2. The log rank stats test was used to determine P-values.

2% Glucose	Mean Survival (generations)	95% confidence interval	# subjects scored
**WT + 0 uM M2I-1**	21.43 ± 1.61	18.27 ~ 24.60	30
**WT + 0.1 uM M2I-1**	23.69 ± 1.55	20.65 ~ 26.73	32
**WT + 1 uM M2I-1**	27 ± 1.47	24.12 ~ 29.88	32
**WT + 10 uM M2I-1**	29.41 ± 1.25	26.95 ~ 31.87	32

Condition	X^2	p-value
**WT + 0 mM M2I vs WT + 0.1 mM M2I**	1.04	0.3087
**WT + 0 mM M2I vs WT + 1 mM M2I**	4.22	0.0399
**WT + 0 mM M2I vs WT + 10 mM M2I**	9.67	0.0019

Supplemental Table 2. Data for **Figure 2D** using the log rank test as part of the OASIS2 stats package.

2% Glucose	Mean Survival (generations)	95% confidence interval	# subjects scored
***apc5* + 0 uM M2I-1**	17.81 ± 0.73	16.37 ~ 19.25	32
***apc5* + 0.1 uM M2I-1**	20.47 ± 0.89	18.72 ~ 22.22	32
***apc5* + 1 uM M2I-1**	19.69 ± 0.87	17.98 ~ 21.40	32
***apc5* + 10 uM M2I-1**	21.44 ± 1.31	18.87 ~ 24.00	32

Condition	X^2	p-value
***apc5* + 0 µM M2I vs WT + 0 µM M2I**	8.69	0.0032
***apc5* + 0 µM M2I vs *apc5* + 0.1 µM M2I**	6.81	0.009
***apc5* + 0 µM M2I vs *apc5* + 1 µM M2I**	3.83	0.0502
***apc5* + 0 µM M2I vs *apc5* + 10 µM M2I**	11.1	0.0009

Supplemental Table 3. Data for **Figure 5A** using a log rank test as part of the OASIS2 stats package.

Trp- 2% Glucose	Mean Survival (generations)	95% confidence interval	# subjects scored
WT + EV	10.14 ± 0.68	8.79 ~ 11.48	51
WT + C3-1B	10.42 ± 0.67	9.11 ~ 11.73	57
WT + C2-4B	15.47 ± 0.72	14.07 ~ 16.88	57
WT + C3-1	9.36 ± 0.49	8.39 ~ 10.33	50
WT + C4-3	10.56 ± 0.8	8.98 ~ 12.13	54
WT + C9-5	9.47 ± 0.39	8.70 ~ 12.13	53
WT + C13-3	15.56 ± 0.95	13.70 ~ 17.42	41
WT+ C43-3	10.91 ± 0.78	9.38 ~ 12.45	46

Condition	X^2	p-value
WT + EV vs WT + C3.1B	0.21	0.647
WT + EV vs WT + C2-4B	19.62	0.0000094
WT + EV vs WT + C3-1	1.32	0.2511
WT + EV vs WT + C4-3	0.23	0.6279
WT + EV vs WT + C9-6	1.32	0.2499
WT + EV vs WT + C13-3	17.74	0.000025
WT + EV vs WT + C43-4	0.29	0.578

Supplemental Table 4. Data for **Figure 5B** using a log rank test as part of the OASIS2 stats package.

2% Glucose	Mean Survival (generations)	95% confidence interval	# subjects scored
WT + EV	20.87 ± 1.45	18.03 ~ 23.72	31
WT + C2.4B	25.66 ± 1.56	22.61 ~ 28.71	32
WT + C13.3	19.63 ± 1.53	16.63 ~ 22.62	32
WT + C43.4	21.9 ± 1.61	18.76 ~ 25.05	31

Condition	X^2	p-value
WT + EV vs WT + C2-4B	3.29	0.0696
WT + EV vs WT + C13-3	0.14	0.713
WT + EV vs WT + C43-4	0.28	0.5975

Supplemental Table 5. Data for **Figure 5C** using a log rank test as part of the OASIS2 stats package.

	Mean Survival (generations)	95% confidence interval	# subjects scored
WT + 2% Glu	22 ± 1.74	18.58 ~ 25.42	32
WT + 0.5% Gal/1.5% Suc	24.47 ± 1.88	20.79 ~ 28.15	32
WT + C43.4 2% Glu	23.63 ± 1.45	20.77 ~ 26.48	32
WT + C43.4 0.5%Gal/1.5% Suc	29.66 ± 1.97	25.79 ~ 33.53	32

Condition	X^2	p-value
WT 2% Glu vs WT + 0.5% Gal	0.96	0.3261
WT 2% Glu vs C43.4 + 2% Glu	0.11	0.7446
WT 2% Glu vs C43.4 + 0.5% Gal	7.66	0.0057

Supplemental Table 6. Mean and max lifespan, and 95% confidence intervals for CLS curves shown in **Figures 6A-E**.

Strain	Mean Survival (Days)	95% Confidence Interval	Max survival (Days)
EV 2% Suc	5.065 ± 0.3029	4.906 to 5.225	9
EV 1% Gal	5.177 ± 0.1609	5.095 to 5.259	9
C2-4B 2% Suc	5.189 ± 0.3483	5.005 to 5.375	9
C2-4B 1% Gal	5.160 ± 0.2871	5.015 to 5.307	9
C9-5 2% Suc	5.169 ± 0.2938	5.014 to 5.324	9
C9-5 1% Gal	5.060 ± 0.2778	4.922 to 5.199	9
C13-3 2% Suc	5.216 ± 0.2339	5.092 to 5.341	9
C13-3 1% Gal	5.102 ± 0.3385	4.935 to 5.270	9
C43-4 2% Suc	5.243 ± 0.2187	5.128 to 5.359	9
C43-4 1% Gal	6.549 ± 0.3936	6.134 to 6.954	13
EV 2% Suc (Ura+)	6.318 ± 0.4558	5.871 to 6.765	13
EV 1% Gal (Ura+)	6.365 ± 0.5360	5.847 to 6.882	13
C43-4 2% Suc (Ura+)	6.533 ± 0.4193	6.132 to 6.936	13
C43-4 1% Gal (Ura+)	8.736 ± 0.3029	8.035 to 9.423	19

Supplemental Table 7. Statistical analysis performed using OASIS for lifespan of N2 wild type worms in the presence or absence of C43-4. Linked to **Figure 11C**.

	Mean survival (Days)	95% confidence interval	# of subjects scored	# of censors	P - value vs corresponding N2
**Lifespan (Trial 1)**					1.2×10^{-6}
**N2**	19.95 ± 0.48	19.00 ~ 20.90	106	3	
**C43.4**	23.45 ± 0.45	22.56 ~ 24.33	148	17	
**Lifespan (Trial 2)**					0.0002
**N2**	19.07 ± 0.44	18.20 ~ 19.94	120	18	
**C43.4**	23.22 ± 0.45	22.33 ~ 24.11	98	26	
**Lifespan (Trial 3)**					0.0002
**N2**	20.61 ± 0.53	19.58 ~ 21.65	98	13	
**C43.4**	23.96 ± 0.47	23.04 ~ 24.89	124	32	

Supplemental Table 8. **(A)** Statistical analysis performed using OASIS showing mean lifespans of N2 wild type and *daf-16* worms in the presence or absence of C43-4. **(B)** P - values comparing lifespan curves for the different combinations used. Linked to **Figure 11D**.

A

	Mean survival (Days)	95% confidence interval	# of subjects scored	# of censors
**Lifespan (Trial 1)**				
**N2**	17.52 ± 0.26	17.01 ~ 18.03	186	2
**C43-4**	19.37 ± 0.35	18.69 ~ 20.06	159	7
***daf-16*(mu86)**	14.05 ± 0.24	13.59 ~ 14.51	129	5
**C43.4; *daf-16*(mu86)**	15.73 ± 0.28	15.18 ~ 16.27	146	5
**Lifespan (Trial 2)**				
**N2**	17.76 ± 0.3	17.17 ~ 18.36	176	1
**C43-4**	19.48 ± 0.31	18.86 ~ 20.09	164	7
***daf-16*(mu86)**	14.65 ± 0.25	14.15 ~ 15.14	155	5
**C43.4; *daf-16*(mu86)**	16.35 ± 0.27	15.82 ~ 16.89	153	7
**Lifespan (Trial 3)**				
**N2**	19.35 ± 0.38	18.60 ~ 20.10	133	2
**C43-4**	21.49 ± 0.41	20.69 ~ 22.29	132	8
***daf-16*(mu86)**	15.26 ± 0.23	14.81 ~ 15.71	148	4
**C43.4; *daf-16*(mu86)**	17.5 ± 0.26	16.99~ 18.02	159	4

B

**Trial 1 condition**	**X^2**	**P – value**
**N2 v.s. C43-4**	23.54	0.0000012
**N2 v.s. *daf-16*(mu86)**	83.08	0
**N2 v.s. C43-4;*daf-16*(mu86)**	19	0.000013
**C43-4 v.s. *daf-16*(mu86)**	130.76	0
**C43-4 v.s. C43-4;*daf-16*(mu86)**	65.74	0
***daf-16*(mu86) v.s. C43-4;*daf-16*(mu86)**	24.05	9.40E-07
**Trial 2 condition**	**X^2**	**P – value**
**N2 v.s. C43-4**	13.98	0.0002
**N2 v.s. *daf-16*(mu86)**	59.39	0
**N2 v.s. C43-4;*daf-16*(mu86)**	13.02	0.0003
**C43-4 v.s. *daf-16*(mu86)**	117.1	0
**C43-4 v.s. C43-4;*daf-16*(mu86)**	52.78	0
***daf-16*(mu86) v.s. C43-4;*daf-16*(mu86)**	20.86	0.0000049
**Trial 3 condition**	**X^2**	**P – value**
**N2 v.s. C43-4**	13.6	0.0002
**N2 v.s. *daf-16*(mu86)**	80.58	0
**N2 v.s. C43-4;*daf-16*(mu86)**	20.27	0.0000067
**C43-4 v.s. *daf-16*(mu86)**	134.31	0
**C43-4 v.s. C43-4;*daf-16*(mu86)**	63.77	0
***daf-16*(mu86) v.s. C43-4;*daf-16*(mu86)**	41.06	0

Supplemental Table 9. **(A)** Statistical analysis performed using OASIS showing mean lifespans of N2 wild type and *aat-2* worms in the presence or absence of C43-4. **(B)** P - values comparing lifespan curves for the different combinations used. Linked to **Figure 11E**.

A

	Mean survival (Days)	95% confidence interval	# of subjects scored	# of censors
**Lifespan (Trial 1)**				
**N2**	13.92 ± 0.16	13.60 ~ 14.23	191	4
**C43.4**	14.92 ± 0.19	14.54 ~ 15.30	197	3
**aak-2(ok524)**	12.43 ± 0.17	12.10 ~ 12.76	164	7
**C43.4; aak-2(ok524)**	13.61 ± 0.17	13.28 ~ 13.94	160	3
**Lifespan (Trial 2)**				
**N2**	16.3 ± 0.3	15.72 ~ 16.88	162	3
**C43.4**	17.95 ± 0.31	17.35 ~ 18.55	166	2
**aak-2(ok524)**	14.96 ± 0.19	14.58 ~ 15.34	175	8
**C43.4; aak-2(ok524)**	15.23 ± 0.23	14.78 ~ 15.67	146	21
**Lifespan (Trial 3)**				
**N2**	17.76 ± 0.3	17.17 ~ 18.36	176	1
**C43.4**	19.62 ± 0.32	18.98 ~ 20.25	164	7
**aak-2(ok524)**	15.6 ± 0.21	15.18 ~ 16.01	189	16
**C43.4; aak-2(ok524)**	15.55 ± 0.28	14.99 ~ 16.10	140	10

B

Trial 1 condition	X^2	P – value
**N2 v.s. C43-4**	18.93	0.000014
**N2 v.s. *aak-2*(ok524)**	34.05	5.30E-09
**N2 v.s. C43-4;*aak-2*(ok524)**	0.98	0.3218
**C43-4 v.s. *aak-2*(ok524)**	82.52	0
**C43-4 v.s. C43-4;*aak-2*(ok524)**	28.74	8.30E-08
***aak-2*(ok524) v.s. C43-4;*aak-2*(ok524)**	22.61	0.000002
**Trial 2 condition**	**X^2**	**P – value**
**N2 v.s. C43-4**	13.66	0.0002
**N2 v.s. *aak-2*(ok524)**	16.9	0.000039
**N2 v.s. C43-4;*aak-2*(ok524)**	8.63	0.0033
**C43-4 v.s. *aak-2*(ok524)**	75.5	0
**C43-4 v.s. C43-4;*aak-2*(ok524)**	52.38	0
***aak-2*(ok524) v.s. C43-4;*aak-2*(ok524)**	1.32	0.2511
**Trial 3 condition**	**X^2**	**P – value**
**N2 v.s. C43-4**	13.98	0.0002
**N2 v.s. *aak-2*(ok524)**	40.44	0
**N2 v.s. C43-4;*aak-2*(ok524)**	28.12	1.10E-07
**C43-4 v.s. *aak-2*(ok524)**	100.01	0
**C43-4 v.s. C43-4;*aak-2*(ok524)**	74.26	0
***aak-2*(ok524) v.s. C43-4;*aak-2*(ok524)**	0.02	0.8792

Supplemental Table 10. (A) Statistical analysis performed using OASIS showing mean lifespans of N2 wild type and *akt-1* worms in the presence or absence of C43-4. **(B)** P - values comparing lifespan curves for the different combinations used. Linked with **Figure 11F**.

A

	Mean survival (Days)	95% confidence interval	# of subjects scored	# of censors
**Lifespan (Trial 1)**				
**N2**	19.12 ± 0.36	18.41 ~ 19.83	133	2
**C43.4**	21.55 ± 0.4	20.76 ~ 22.34	135	8
***akt-1*(ok525)**	23.02 ± 0.55	21.94 ~ 24.10	184	4
**C43.4; *akt-1*(ok525)**	23.79 ± 0.71	22.40 ~ 25.18	146	7
**Lifespan (Trial 2)**				
**N2**	19.83 ± 0.32	19.20 ~ 20.46	162	7
**C43.4**	20.51 ± 0.4	19.73 ~ 21.28	134	7
***akt-1*(ok525)**	22.65 ± 0.56	21.54 ~ 23.75	131	1
**C43.4; *akt-1*(ok525)**	22.97 ± 0.58	21.82 ~ 24.11	126	15
**Lifespan (Trial 3)**				
**N2**	17.86 ± 0.29	17.28 ~ 18.43	156	4
**C43.4**	20.28 ± 0.39	19.51 ~ 21.05	124	11
***akt-1*(ok525)**	22.75 ± 0.44	21.90 ~ 23.60	159	9
**C43.4; *akt-1*(ok525)**	23.74 ± 0.6	22.56 ~ 24.91	119	6

B

Trial 1 condition	X^2	P – value
**N2 v.s. C43-4**	21.03	0.0000045
**N2 v.s. *akt-1*(ok525)**	41.21	0
**N2 v.s. C43-4;*akt-1*(ok525)**	42.45	0
**C43-4 v.s. *akt-1*(ok525)**	13.89	0.0002
**C43-4 v.s. C43-4;*akt-1*(ok525)**	19.52	0.00001
***akt-1*(ok525) v.s. C43-4;*akt-1*(ok525)**	3.27	0.0708
**Trial 2 condition**	**X^2**	**P – value**
**N2 v.s. C43-4**	3.59	0.0581
**N2 v.s. *akt-1*(ok525)**	34.23	4.90E-09
**N2 v.s. C43-4;*akt-1*(ok525)**	38.03	0
**C43-4 v.s. *akt-1*(ok525)**	17.2	0.000034
**C43-4 v.s. C43-4;*akt-1*(ok525)**	20.77	0.0000052
***akt-1*(ok525) v.s. C43-4;*akt-1*(ok525)**	0.21	0.6454
**Trial 3 condition**	**X^2**	**P – value**
**N2 v.s. C43-4**	26.94	2.10E-07
**N2 v.s. *akt-1*(ok525)**	84.84	0
**N2 v.s. C43-4;*akt-1*(ok525)**	88.73	0
**C43-4 v.s. *akt-1*(ok525)**	22.2	0.0000025
**C43-4 v.s. C43-4;*akt-1*(ok525)**	33.04	9.00E-09
***akt-1*(ok525) v.s. C43-4;*akt-1*(ok525)**	3.63	0.0567
